# Supplementary material for: Ethnic differences in guideline-indicated statin initiation for people with type 2 diabetes in UK primary care, 2006–2019: A cohort study
Source: PLoS Med. 2021 Jun 29;18(6):e1003672. doi: 10.1371/journal.pmed.1003672 (PMC8241069; doi:10.1371/journal.pmed.1003672)
Supplement: S1 Fig — (DOCX) [file pmed.1003672.s002.docx]

**Figure S1. Associations between ethnicity and guideline-indicated statin initiation after type 2 diabetes diagnosis: sub-group analyses by a) age group at type 2 diabetes diagnosis, b) country and c) gender.** Data are hazard ratios (HRs, marker) and 95% CI (capped lines) adjusted for age, gender, deprivation, smoking, healthcare usage, TC/HDL(total to high-density lipoprotein cholesterol ratio), BMI (body mass index), prevalent comorbidity, medication usage and antihypertensive usage, from multi-level models accounting for intra-practice clustering. European ethnicity = referent category, i.e. HR for people of European ethnicity=1.
