# Supplementary material for: Ethnic differences in guideline-indicated statin initiation for people with type 2 diabetes in UK primary care, 2006–2019: A cohort study
Source: PLoS Med. 2021 Jun 29;18(6):e1003672. doi: 10.1371/journal.pmed.1003672 (PMC8241069; doi:10.1371/journal.pmed.1003672)
Supplement: S2 Fig — (DOCX) [file pmed.1003672.s003.docx]

**Figure S2. Associations between ethnicity and guideline-indicated statin initiation after type 2 diabetes diagnosis: sensitivity analyses, adjusting for a) HbA_1c_, b) patient-level Index of Multiple Deprivation quintile, c) non-HDL cholesterol instead of TC/ HDL ratio and d) BMI as a categorical instead of a continuous variable, and excluding e) people with recorded reasons for statin declinature and f) people exception-reported from diabetes Quality Outcome Framework indicators.** Data are HRs (marker) and 95% CI (capped lines) adjusted for age, gender, deprivation, smoking, healthcare usage, TC/HDL, BMI, prevalent comorbidity, medication usage and antihypertensive usage, from multi-level models accounting for intra-practice clustering. European ethnicity = referent category, i.e. HR for people of European ethnicity=1.

*HbA_1c_= glycosylated haemoglobin A_1c_, IMD= index of multiple deprivation, HDL=high density lipoprotein, TC/ HDL= total cholesterol to HDL-cholesterol ratio, BMI=body mass index, T2DM=type 2 diabetes mellitus, QOF= quality outcomes framework*
