# Supplementary material for: Ethnic differences in guideline-indicated statin initiation for people with type 2 diabetes in UK primary care, 2006–2019: A cohort study
Source: PLoS Med. 2021 Jun 29;18(6):e1003672. doi: 10.1371/journal.pmed.1003672 (PMC8241069; doi:10.1371/journal.pmed.1003672)
Supplement: S3 Fig — (DOCX) [file pmed.1003672.s004.docx]

**Figure S3****. Log-log plot of “survival”, i.e. statin initiation, by ethnicity.**
