# Supplementary material for: Ethnic differences in guideline-indicated statin initiation for people with type 2 diabetes in UK primary care, 2006–2019: A cohort study
Source: PLoS Med. 2021 Jun 29;18(6):e1003672. doi: 10.1371/journal.pmed.1003672 (PMC8241069; doi:10.1371/journal.pmed.1003672)
Supplement: S1 Protocol — (DOCX) [file pmed.1003672.s006.docx]

INDEPENDENT SCIENTIFIC ADVISORY COMMITTEE (ISAC) PROTOCOL APPLICATION FORM

PART 1: APPLICATION FORM

***IMPORTANT***

**Both parts of this application must be completed in accordance with the guidance note ‘Completion of the ISAC Protocol Application Form’, which can be found on the CPRD website** [**cprd.com/research-applications**](https://cprd.com/research-applications)

| FOR ISAC USE ONLY | |
| --- | --- |
| **Protocol No. -** | **Submission date -** |

| GENERAL INFORMATION ABOUT THE PROPOSED RESEARCH STUDY |
| --- |
| Study Title (Max. 255 characters) Ethnic differences in the prescribing of anti-diabetic, antihypertensive and lipid-lowering medication for people with and without type 2 diabetes. |
| **Research Area** (place ‘X’ in all boxes that apply) |
| \| Drug Safety \|  \| Economics \|  \| \| --- \| --- \| --- \| --- \| \| Drug Utilisation \| X \| Pharmacoeconomics \|  \| \| Drug Effectiveness \|  \| Pharmacoepidemiology \| X \| \| Disease Epidemiology \|  \| Methodological \|  \| \| Health Services Delivery \| X \|  \|  \| |
| Chief Investigator  \| Title: \| Professor \| \| --- \| --- \| \| Full name: \| Krishnan Bhaskaran \| \| Job title: \| Professor of Statistical Epidemiology and Sir Henry Dale Fellow \| \| Affiliation/organisation: \| London School of Hygiene and Tropical Medicine \| \| Email address: \| krishnan.bhaskaran@lshtm.ac.uk \| \| CV Number (if applicable): \| 156_15CESL \| |
| Corresponding Applicant  \| Title: \| Dr \| \| --- \| --- \| \| Full name: \| Sophie Eastwood \| \| Job title: \| Diabetes UK Sir George Alberti Clinical Research Training Fellow \| \| Affiliation/organisation: \| University College London \| \| Email address: \| sophie.eastwood@ucl.ac.uk \| \| CV Number (if applicable): \| 221_17 \| |
| List of all investigators/collaborators  \| Title: \| Professor \| \| --- \| --- \| \| Full name: \| Nish Chaturvedi \| \| Job title: \| Professor of Clinical Epidemiology \| \| Affiliation/organisation: \| University College London \| \| Email address: \| n.chaturvedi@ucl.ac.uk \| \| CV Number (if applicable): \| 220_17 \| \| Will this person be analysing the data? (Y/N) \| N \|  \| Title: \| Professor \| \| --- \| --- \| \| Full name: \| Liam Smeeth \| \| Job title: \| Professor of Clinical Epidemiology \| \| Affiliation/organisation: \| London School of Hygiene and Tropical Medicine \| \| Email address: \| liam.smeeth@lshtm.ac.uk \| \| CV Number (if applicable): \| 045_15CEPSL \| \| Will this person be analysing the data? (Y/N) \| N \| \| Title: \| Dr \| \| Full name: \| Rohini Mathur \| \| Job title: \| Assistant Professor of Epidemiology and Sir Henry Wellcome Postdoctoral Fellow \| \| Affiliation/organisation: \| London School of Hygiene and Tropical Medicine \| \| Email address: \| rohini.mathur@lshtm.ac.uk \| \| CV Number (if applicable): \| 316_15CESL \| \| Will this person be analysing the data? (Y/N) \| N \| \| Title: \| Dr \| \| Full name: \| Ruth Farmer \| \| Job title: \| Research Fellow in Statistical Epidemiology \| \| Affiliation/organisation: \| London School of Hygiene and Tropical Medicine \| \| Email address: \| ruth.farmer@lshtm.ac.uk \| \| CV Number (if applicable): \| 222_17 \| \| Will this person be analysing the data? (Y/N) \| N \| \| Title: \| Professor \| \| Full name: \| Naveed Sattar \| \| Job title: \| Professor of Metabolic Medicine \| \| Affiliation/organisation: \| University of Glasgow, University of Oxford \| \| Email address: \| naveed.sattar@glasgow.ac.uk \| \| CV Number (if applicable): \| - \| \| Will this person be analysing the data? (Y/N) \| N \| \| Title: \| Professor \| \| Full name: \| Andrew Hattersley \| \| Job title: \| Professor of Molecular Medicine \| \| Affiliation/organisation: \| University of Exeter \| \| Email address: \| a.t.hattersley@exeter.ac.uk \| \| CV Number (if applicable): \| 345_15C \| \| Will this person be analysing the data? (Y/N) \| N \|   [Add more investigators/collaborators as necessary by copy and pasting a new table for each investigator/collaborator] |
| Experience/expertise available List below the member(s) of the research team who have experience with CPRD data.   \| **Name:** \| **Protocol Number/s:** \| \| --- \| --- \| \| Krishnan Bhaskaran \| 16_174, 17_087, 12_027RA \| \| Sophie Eastwood \| 17_087 \| \| Nish Chaturvedi \| 17_087, 12_027RA \| \| Liam Smeeth \| 16_174, 17_087, 12_027RA \| \| Rohini Mathur \| 17_087 \| \| Ruth Farmer \| 12_027RA \| \| Andrew Hattersley \| 13_177R \|   List below the member(s) of the research team who have statistical expertise.   \| **Name(s):** \|  \| \| --- \| --- \| \| Krishnan Bhaskaran \| \| \| Ruth Farmer \| \|   List below the member(s) of the research team who have experience of handling large datasets (greater than 1 million records).   \| **Name(s):** \|  \| \| --- \| --- \| \| Krishnan Bhaskaran \| \| \| Liam Smeeth \| \| \| Rohini Mathur \| \| \| Ruth Farmer \| \|   List below the member(s) of the research team, or supporting the research team, who have experience of practicing in UK primary care.   \| **Name(s):** \|  \| \| --- \| --- \| \| Liam Smeeth \| \| \| Sophie Eastwood \| \| |
| ACCESS TO THE DATA |
| Sponsor of the study  \| Institution/Organisation: \| London School of Hygiene and Tropical Medicine \| \| --- \| --- \| \| Address: \| Keppel Street, London, United Kingdom, WC1E 7HT \| |
| Funding source for the study  \| Same as Sponsor? \| Yes \|  \| No \| X \|  \| \| --- \| --- \| --- \| --- \| --- \| --- \| \| Institution/Organisation: \| Diabetes UK \| \| \| \| \| \| Address: \| Wells Lawrence House, 126 Back Church Lane, London, E1 1FH \| \| \| \| \| |
| Institution conducting the research  \| Same as Sponsor? \| Yes \| X \| No \|  \|  \| \| --- \| --- \| --- \| --- \| --- \| --- \| \| Institution/Organisation: \|  \| \| \| \| \| \| Address: \|  \| \| \| \| \| |
| Data Access Arrangements Indicate with an ‘**X**’ the method that will be used to access the data for this study:   \| Study-specific Dataset Agreement \|  \| \| --- \| --- \|  \| Institutional Multi-study Licence \| X \|  \| \| --- \| --- \| --- \| \| Institution Name \| London School of Hygiene and Tropical Medicine \| \| \| Institution Address \| Keppel Street, London, United Kingdom, WC1E 7HT \| \|   Will the dataset be extracted by CPRD?   \| Yes \|  \| No \| X \| \| --- \| --- \| --- \| --- \|   If yes, provide the reference number: |
| 1. **Data Processor(s):**  \| Processing \| X \|  \| \| --- \| --- \| --- \| \| Accessing \| X \| \| Storing \| X \| \| Processing area (UK/EEA/Worldwide) \| \| UK \| \| Organisation name \| \| London School of Hygiene and Tropical Medicine \| \| Organisation address \| \| Keppel Street, London, United Kingdom, WC1E 7HT \|   [Add more processors as necessary by copy and pasting a new table for each processor] |
| INFORMATION ON DATA |
| Primary care data (place ‘X’ in all boxes that apply)  \| CPRD GOLD \| X \| CPRD Aurum \|  \| \| --- \| --- \| --- \| --- \|   **X** |
| Please select any linked data or data products being requested **Patient Level Data** (place ‘**X**’ in all boxes that apply) |
| \| ONS Death Registration Data \|  \| CPRD Mother Baby Link \|  \| \| --- \| --- \| --- \| --- \| \| HES Admitted Patient Care \| X \| Pregnancy Register \|  \| \| HES Outpatient \|  \| NCRAS (National Cancer Registration and Analysis Service) Cancer Registration Data \|  \| \| HES Accident and Emergency \|  \| NCRAS Cancer Patient Experience Survey (CPES) data \|  \| \| HES Diagnostic Imaging Dataset \|  \| NCRAS Systemic Anti-Cancer Treatment (SACT) data \|  \| \| HES PROMS (Patient Reported Outcomes Measure) \|  \| NCRAS National Radiotherapy Dataset (RTDS) data \|  \| \|  \|  \| Mental Health Services Data Set (MHDS) \|  \| |
| **Area Level Data** (place ‘**X**’ in all boxes that apply)   \| **Practice level (UK)** \|  \| **Patient level (England only)** \|  \| \| --- \| --- \| --- \| --- \| \| Practice Level Index of Multiple Deprivation (Standard) \| X \| Patient Level Index of Multiple Deprivation \| X \| \| Practice Level Index of Multiple Deprivation (Non-standard) \|  \| Patient Level Townsend Score \|  \| \| Practice Level Index of Multiple Deprivation Domains (Non-standard) \|  \|  \|  \| \| Practice Level Carstairs Index for 2011 Census (Excluding Northern Ireland) (Standard) \|  \|  \|  \| \| 2011 Rural-Urban Classification at LSOA level (Non-standard) \|  \|  \|  \|   Reference number (where applicable): |
| Are you requesting linkage to a dataset not listed above?  \| Yes \| **X** \| No \|  \| \| --- \| --- \| --- \| --- \|   If yes, provide the reference number: |
| Does any person named in this application already have access to any of these data in a patient identifiable form, or associated with an identifiable patient index?  \| Yes \|  \| No \| **X** \| \| --- \| --- \| --- \| --- \|   If yes, provide further details: |
| VALIDATION/VERIFICATION |
| Does this protocol describe an observational study using purely CPRD data?  \| Yes \| **X** \| No \|  \| \| --- \| --- \| --- \| --- \| |
| Does this protocol involve requesting any additional information from GPs, or contact with patients?  \| Yes \|  \| No \| **X** \| \| --- \| --- \| --- \| --- \|   If yes, provide the reference number: |

**PART 2: PROTOCOL INFORMATION**

| **Applicants must complete all sections listed below**  **Sections which do not apply should be completed as ‘*Not Applicable’* and justification provided** |
| --- |
| Study Title (Max. 255 characters) Ethnic differences in the prescribing of anti-diabetic, antihypertensive and lipid-lowering medication for people with type 2 diabetes. |
| Lay Summary (Max. 250 words) The UK’s South Asian and African Caribbean populations experience far higher rates of type 2 diabetes and its cardiovascular disease complications, e.g. heart disease and strokes, than European-origin (white) groups. Control of blood glucose, high blood pressure and high cholesterol helps to reduce cardiovascular disease complications from diabetes. Despite this, we know little about ethnic differences in the use of medications which do this. Limited existing research suggests that both choice and effective use of diabetes, blood pressure or cholesterol-lowering medication may differ by ethnicity.  This study aims to compare use of these medications for people with diabetes of European, South Asian and African Caribbean origin. Using computerised medical records, the commencement, type, dose, monitoring and adjustment of medication will be studied, and reasons for differences sought.  By identifying ethnic disparities in the use of medications to control diabetes, high blood pressure and high cholesterol, we may highlight reasons for the excess of diabetes complications seen in UK South Asian or African Caribbean groups. These findings may influence prescribing policies, and thus ultimately reduce ethnic differences in the cardiovascular complications of type 2 diabetes. |
| Technical Summary (Max. 300 words) UK South Asian and African Caribbean people with type 2 diabetes experience worse diabetic control and more cardiovascular complications than the European-origin population. Explanations are unclear. Blood pressure and lipid control, crucial to cardiovascular risk reduction in diabetes, also differ by ethnicity. However, ethnic differences in prescribing for diabetes, hypertension and hyperlipidaemia remain understudied.  Ethnic differences in prescribing anti-diabetic, antihypertensive and lipid-lowering medication for people with type 2 diabetes will be sought, including: i) time to commencement ii) choice of medication, iii) dosage, iv) monitoring and iv) time to intensification (either medication up-titration, addition or class switching) after detection of sub-optimal control.  Primary care electronic medical records will be used. Established algorithms and code lists will define ethnicity, diagnoses and medication use. Data will be analysed using Kaplan-Meier time-to-event methods and logistic, Poisson and Cox regression models (according to outcome), with South Asian or African Caribbean ethnicity as the exposure (baseline category=European). These methods will allow for differences in follow-up time. The potentially confounding or mediating influences of age, sex, smoking, BMI, deprivation, polypharmacy, multi-morbidity, drug adherence, patient engagement and diabetes duration/ HbA1c will be explored. |
| Outcomes to be Measured Time to first anti-diabetic prescription; Choice of anti-diabetic/ antihypertensive/ lipid-lowering medication; Dose of anti-diabetic/ antihypertensive/ lipid-lowering medication; Monitoring of anti-diabetic/ antihypertensive/ lipid-lowering medication; Time to anti-diabetic/ antihypertensive/ lipid-lowering treatment intensification. |
| Objectives, Specific Aims and Rationale Overall aim  To investigate ethnic differences in prescribing for type 2 diabetes in UK primary care.  Objectives  For people of European, South Asian and African Caribbean origin with type 2 diabetes, ethnic differences in the following will be investigated:  i) Time to initiation of first-line anti-diabetic medication  ii) Choice of anti-diabetic (first- to third-line), antihypertensive and lipid-lowering medication  iii) Doses used for each anti-diabetic, antihypertensive and lipid-lowering medication  iv) Monitoring rate after medication commencement  v) Time to medication intensification (up-titration, addition or switching) after detection of sub-optimal control of diabetes, hypertension or blood lipids  Additionally, we will examine potential confounding or mediating role/s of:  vi) Smoking, BMI, deprivation, polypharmacy, multi-morbidity, drug adherence (for objectives iv) and v)), patient engagement with healthcare, diabetes duration and HbA1c.  Null hypotheses by objective  i) No ethnic difference in time to initiation of first-line anti-diabetic will be detected.  ii) Regarding choice of medication:  a) No ethnic difference in the proportions of people prescribed metformin as first-line, or sulphonylureas, DPP4-inhibitors, thiazolidinediones, SGLT2 inhibitors, GLP1 agonists or insulin as second- or third-line diabetes treatment will be observed.  b) No ethnic difference in the proportions of people prescribed ACE inhibitors/ angiotensin-receptor blockers, calcium-channel blockers or thiazide diuretics for hypertension will be observed.  c) No ethnic difference in the proportions of people with ≥10% cardiovascular disease risk who are prescribed lipid-lowering medication will be observed.  iii) No ethnic difference in the proportions prescribed the maximum dose of anti-diabetic, antihypertensive or lipid-lowering medication will be observed, and the modal dose for each class will not differ by ethnicity.  iv) No ethnic difference in the monitoring rates of anti-diabetic, antihypertensive or lipid-lowering medication will be observed.  v) No ethnic difference in time to treatment intensification (after the detection of sub-optimal control) of anti-diabetic, antihypertensive or lipid-lowering medication will be observed.  vi) Associations between ethnicity and prescribing will not be confounded or mediated by smoking, BMI, deprivation, polypharmacy, multi-morbidity, drug adherence, patient engagement, diabetes duration of HbA1c.  Rationale  Research indicates that control of diabetes and its allied cardiovascular risk factors is poorer in UK’s minority ethnic groups than in the background white population. Crucially, these discrepancies are likely to contribute to higher rates of serious diabetes complications, such as heart disease and stroke, seen in these groups. By examining the effect sizes of the associations above, this study seeks to determine whether ethnic differences in prescribing for diabetes are present, and explanations for them. If ethnic inequalities in prescribing are present, their identification may influence future prescribing recommendations and thus eventually lessen the personal and societal burden of diabetes complications in ethnic minority groups. |
| Study Background The UK’s largest ethnic minority groups, people of South Asian and African Caribbean descent, are disproportionately affected by type 2 diabetes (1). UKPDS (UK Prospective Study of Diabetes) established the importance of tight blood glucose control in reducing CVD complications (2), but research suggests that glucose control is poorer in UK South Asians and African Caribbeans than Europeans (3, 4), despite their greater prevalence of complications(5). Yet the use of anti-diabetic medication in these groups remains understudied. Blood pressure and lipid control is key in reducing CVD risk in people with diabetes (6), therefore we will also examine prescribing patterns for antihypertensives and lipid-lowering medication.  UK guidelines recommend metformin as first-line anti-diabetic therapy, with several choices of further agents before insulin use (7). Recent studies have reported on the choice of second-line treatments in CPRD (8 and 9; ISAC protocol number 16_267), with some indication that people from ethnic minority groups are less likely to receive newer anti-diabetic drugs (9). However, the influence of key factors such as diabetes duration and adherence to treatment were not accounted for, and the related questions of monitoring frequency and expediency of appropriate treatment intensification were not studied. Moreover, evidence from smaller primary care databases suggests South Asians and African Caribbeans are less likely than Europeans to receive insulin, and more likely to be on a higher number of oral diabetes drugs (4, 10). UK hypertension guidelines advocate calcium-channel blockers (CCBs) as first-line treatment for African Caribbeans, and ethnic differences in monotherapy are established (11), but little examination of ethnic differences in prescribing exists beyond this. Studies from aggregated primary care data indicate that statins may be under-prescribed in areas with large ethnic minority populations (12, 13), but were limited by analysis at area level.  Delay in anti-diabetic treatment intensification is linked to poorer subsequent control (14), but in spite of the poorer diabetic control in ethnic minority groups, ethnic differences in time to intensification have never been studied. Equally, an absence of research on ethnic differences in intensification of antihypertensives exists (15). However, previous research indicates that African Caribbean people are less likely than their white counterparts to be prescribed statins (16), though it is unclear if this is the case for people with diabetes. Additionally, whilst evidence from CPRD indicates that hypercholesterolaemia with high cardiovascular risk is inadequately treated in people with diabetes (17), scrutiny by ethnicity is lacking. |
| Study Type Hypothesis testing |
| Study Design Observational cohort study using UK primary care electronic health records |
| Feasibility counts The primary analysis will be using data from CPRD alone (see section M for sensitivity analyses). Ethnicity recording for active patients is approximately 50% (18, 19). The ethnic breakdown is similar to that of the UK census 2011(20).  From 2000-2017, ≈280K patients initiated first-line and ≈80K second-line treatment for type 2 diabetes in CPRD (8). If 50% of these people had ethnicity codes present in their record, this would correspond to ≈140K starting first-line and ≈40K starting second line treatment. Using the 2011 census to calculate proportions, this would equate to ≈120.4K Europeans, ≈10.5K South Asians and ≈4.6K African Caribbeans initiating first-line treatment and 34.4K, 3.0K and 1.3K respectively starting second-line treatment. These are conservative estimates given the over-representation of ethnic minorities in the diabetic population.  Approximately 77% of the people initiating first-line treatments are likely to be on antihypertensives (21), giving numbers for antihypertensive analyses of ≈92.7K Europeans, ≈8.1K South Asians, ≈3.5K African Caribbeans.  Previous work in CPRD shows that ≈64% of people with type 2 diabetes receive statins (21), which will yield numbers for the lipid-lowering medication analyses of ≈77.1K Europeans, ≈6.7K South Asians and ≈2.9K African Caribbeans. |
| Sample size considerations Anti-diabetic analyses  Sample sizes as per “feasibility counts” section, see table 1(below) for detectable differences in ethnic difference effect sizes. Corresponding to objective i), the proportion prescribed an anti-diabetic at 1 year post-diagnosis (42%) is taken from Sinclair et al (22) (detectable differences calculated using stpower cox command in Stata). Corresponding to objective ii), proportions prescribed metformin as first-line (73%) and a sulphonylurea as second line treatment (87%) are taken from Wilkinson et al (8) (power twoproportions command). Corresponding to objectives iv) and v), mean monitoring rate (2.45 times per year [extrapolated], assuming a SD of 2) (power twomeans command) and probability of treatment intensification at 1 year (24%) are taken from Khunti et al (23) (stpower cox command).  Antihypertensive analyses  Sample sizes as per “feasibility counts” section. Little prior data exists in this area, but we can assume a mean time to measurement of 1 month (with an assumed SD of 1 month), as per guidelines for re-measurement after intensification (24). This would give a detectable ethnic difference in time until first monitoring as 0.04 months for South Asians vs. Europeans (using the power twomeans command in Stata), and 0.06 months for African Caribbeans vs. Europeans.  Lipid-lowering medication analyses  Sample sizes as per “feasibility counts” section. Assuming a mean time to monitoring of 3 months (25) and an SD of 1 month, the detectable ethnic difference in time until first monitoring would be 0.04 months for South Asians vs. Europeans, and 0.06 months for African Caribbeans vs. Europeans (using the power twomeans command in Stata).  All detectable differences fall below what is likely to be considered clinically significant.  **Table 1. Detectable ethnic differences in outcomes.**   \| **Outcome** \| **Exposure (ethnicity): N vs. N** \| **Detectable ethnic difference effect sizes at α=0.05, β=0.90** \| \| --- \| --- \| --- \| \| Time from diagnosis to first anti-diabetic prescription \| South Asians vs. Europeans:  10.5K vs. 120.4K \| HR: 1.03 \| \| African Caribbeans vs. Europeans:  4.6K vs. 120.4K \| HR: 1.03 \| \| % prescribed metformin first-line \| South Asians vs. Europeans:  10.5K vs. 120.4K \| Difference in proportions: 1.4% \| \| African Caribbeans vs. Europeans:  4.6K vs. 120.4K \| Difference in proportions: 2.1% \| \| % prescribed sulphonylurea second-line \| South Asians vs. Europeans:  3.0K vs. 34.4K \| Difference in proportions: 2.0% \| \| African Caribbeans vs. Europeans:  1.3K vs. 34.4K \| Difference in proportions: 2.9% \| \| Mean monitoring rate, measurements per person year at risk \| South Asians vs. Europeans:  10.5K vs. 120.4K \| Rate difference: 0.07 \| \| African Caribbeans vs. Europeans:  4.6K vs. 120.4K \| Rate difference: 0.10 \| \| Time from sub-optimal diabetes control to treatment intensification \| South Asians vs. Europeans:  10.5K vs. 120.4K \| HR: 1.04 \| \| African Caribbeans vs. Europeans:  4.6K vs. 120.4K \| HR: 1.04 \| |
| Planned use of linked data (if applicable): Linkage to practice- and patient-level IMD scores is requested to investigate the potential influence of deprivation on any associations found. Both measures are requested to enable sensitivity analyses on the subset with linked patient-level IMD data. Linkage to Hospital Episode Statistics data is requested for a sensitivity analysis using only the 60% of participants with linked data (19), with ethnicity ascertained from HES. Given the higher levels of ethnicity recording in HES than CPRD, this will boost ascertainment of our key exposure up to 80% (18). |
| Definition of the Study population All permanently-registered patients with research-standard data and incident type 2 diabetes Read codes from at least 1 year after 1^st^ January 2006 (start of follow-up; coinciding with when ethnicity was recorded for ≈80% of new registrations (18)) to 31^st^ December 2018 in the CPRD. The study will be restricted to incident cases to enable more precise measurement of treatment stage; people with prevalent type 2 diabetes Read codes prior to 2006 will be excluded.  Type 2 diabetes will be defined by a previously tested algorithm that uses Read codes, medication, blood test results and diabetes-specific process of care Read codes to adjudicate diabetes status (26) – see appendix. Specific exclusions will include: type 1 diabetes, gestational diabetes, non-specified diabetes diagnosed at ≤40 years in Europeans or ≤35 years in South Asians and African Caribbeans (reducing misclassified type 1 diabetes), and secondary diabetes (e.g. drug-induced). |
| Selection of comparison group(s) or controls The UK’s 3 main ethnic groups will be compared, with the following comparisons undertaken for each study sub-question: South Asians versus Europeans, African Caribbeans versus Europeans. Ethnicity will be designated by patient self-report using the 9i% or 9s% Read code hierarchies in CPRD – see appendix for code list. The range of codes will be mapped to the ethnicity codes present in the 2011 UK census (20), with discrepancies or multiple codes being resolved using an algorithm which identifies the most common or most recent codes available (18). For the sensitivity analysis where ethnicity is designated from HES data, usable entries under the “ethnos” code will be collapsed into the 16 ethnicity codes of the UK census. |
| Exposures, outcomes and covariates *Data sources are listed in square parentheses*  Exposures:  For all objectives, the exposure is either South Asian or African Caribbean ethnicity (defined as above), with European ethnicity as the baseline category. *[Primary care clinical records]*  Health Outcomes by study objective:   1. Time to first anti-diabetic prescription. *[Primary care clinical records, prescription drug files.]* 2. Choice of a) anti-diabetic (first- to third-line), b) antihypertensive and c) lipid-lowering medications, see appendix for code lists for each (derived from chapters of the British National Formulary). *[Prescription drug files.]* 3. Doses used for each a) anti-diabetic, b) antihypertensive and c) lipid-lowering medication. *[Prescription drug files.]* 4. Monitoring rate, stratified by the time since treatment initiation, e.g. the number of a) HbA1c, b) BP or c) lipid measures divided by the amount of person time, within strata of time since initiation (i.e. first year, second year since initiation etc). *[Prescription drug files, test records.]* 5. Time to treatment intensification after detection of sub-optimal risk factor control. *[Primary care clinical records, prescription drug files, test records.]* Sub-optimal control will be classified as follows:    1. Diabetes; HbA1c≥53mmol/mol (7.5%) (7)    2. Hypertension; blood pressure≥140/80 mmHg, or 130/80 mmHg in those with neuropathy/ nephropathy or retinopathy (24)    3. Lipids; <40% reduction in non-HDL cholesterol (25) 6. Influence of the following on associations between ethnicity and prescribing: smoking, BMI, deprivation, polypharmacy, multi-morbidity, drug adherence (for outcomes iv) and v)), patient engagement with healthcare, diabetes duration and HbA1c. *[Linked IMD data, primary care clinical records, prescription drug files, test records.]*   Covariates (all taken from last entry in medical notes prior to follow-up time commencing)  Age *[Primary care clinical records]*  Sex *[Primary care clinical records]*  Smoking status *[Primary care clinical records]*  BMI *[Primary care clinical records]*  Deprivation (using patient-level IMD scores, with sensitivity analyses using practice-level IMD scores) *[Primary care clinical records, linked deprivation data]*  Polypharmacy (number of medications) *[Prescription drug files]*  Multimorbidity (defined by presence or absence of the following key co-morbidities: coronary heart disease, cerebrovascular disease, heart failure, atrial fibrilliation, hypertension, chronic kidney disease, peripheral arterial disease, retinopathy, neuropathy, cancer [any], COPD/ asthma, severe mental illness) *[Primary care clinical records]*  Patient engagement/ access (using the number of consultations in the year preceding diabetes diagnosis as a proxy) *[Primary care clinical records]*  Patient adherence (using the proportion of days covered: PDC=days of medication supplied per x day period/ x (27)). This will be calculated separately for all medications issued over the follow-up time of interest, and then a mean PDC for all medications will be derived for each patient. Both measures are important as adherence may vary by drug. *[Prescription drug files]*  Disease parameters: baseline HbA1c/ blood pressure and number of years since diabetes diagnosis *[Primary care clinical records, test records.]* |
| Data/ Statistical Analysis For all parts of the study, the population will be people with new-onset type 2 diabetes in CPRD from 2006 (see section K, above) and the exposure will be ethnicity (either South Asian vs. European [baseline] or African Caribbean vs. European [baseline], as detailed in section L, above). Covariates in base models will include age and sex, and further covariates will be considered individually, as outlined in section vi) below. Statistical analysis is further considered by objective:   1. **Outcome**: time to initiation of first-line anti-diabetic medication, probability of receiving anti-diabetic 1 year post-diagnosis   **Entry**: date of type 2 diabetes diagnosis  **Exit**: date of first anti-diabetic prescription (defined by the absence of a prescription for the medication in the previous 6 months’ records), or end of study/ leaving CPRD practice/ death  **Analysis**: summary statistics will be compared by ethnicity using Kaplan-Meier time-to-event analysis (cumulative failure probability in a given time frame by ethnicity, comparison of medians by log-rank test), Cox proportional hazards regression models will be used to adjust for confounding/ investigate potential mediators  **Example effect measure**: hazards ratio (HR) for anti-diabetic prescription   1. **Outcome:** proportions of patients prescribed medication from each anti-diabetic (stratified by first- to third-line treatment), antihypertensive and lipid-lowering class (defined by the absence of a prescription for the medication in the previous 6 months’ records)   **Sub-population:** a) all participants starting first- to third-line anti-diabetic treatment, b) all patients on antihypertensives, or c) all patients on lipid-lowering medication  **Analysis:** proportions receiving each drug will be compared by ethnicity, then logistic regression models will be used to adjust for confounding/ investigate potential mediators  **Example effect measures**: odds ratio (OR) of being prescribed metformin (=1) vs. non-metformin medication (=0) at first stage of treatment, OR of being prescribed sulphonylurea (=1) vs. non- sulphonylurea medication (=0) at second stage of treatment, OR of calcium-channel blocking antihypertensive prescription vs. non- calcium-channel blocking antihypertensive in those on antihypertensive treatment, OR of statin prescription vs. non-statin lipid-lowering medication prescription in those with >10% 10 year cardiovascular risk   1. **Outcome:** proportions of patients prescribed each dose of each anti-diabetic, antihypertensive and lipid-lowering medication, stratified by treatment stage (defined by the absence of a prescription for the medication in the previous 6 months’ records)   **Sub-population:** a) all participants on first- to third-line anti-diabetic treatment, b) all patients on antihypertensives, or c) all patients on lipid-lowering medication  **Analysis: :** proportions receiving each dose of each drug will be compared by ethnicity, then logistic regression models will be used to adjust for confounding/ investigate potential mediators  **Example effect measures**: OR of being prescribed maximum dose (2g daily) vs. sub-maximum dose of metformin, OR of being prescribed atorvastatin 10mg per day vs. higher doses (20mg, 40mg or 80mg)   1. **Outcome:** yearly monitoring rate post commencement of medication from each anti-diabetic (first- to third-line), antihypertensive and lipid-lowering class – i.e. number of times HbA1c/ blood pressure/ blood lipids measured per person-year of follow-up, stratified by year after commencement; may be multiple entry dates per person for different medications   **Entry:** first use of any medications of interest from 2006 onwards  **Exit:** first of; end of strata (e.g. 1 year after medication commencement), end of study/ leaving CPRD practice/ death  **Analysis:** summary statistics will be compared by ethnicity, Poisson regression modelling used to adjust for confounding/ investigate potential mediators  **Example effect measures**: yearly monitoring rate ratios (RRs)   1. **Outcome:** time to medication intensification (up-titration, addition or switching) after detection of sub-optimal control of diabetes, hypertension or blood lipids, or probability of intensification 1 year post-detection of sub-optimal control; may be multiple entry dates per person for different medications   **Entry:** date when sub-optimal risk factor control detected (see section M for how this is defined for each risk factor)  **Exit:** first of; date of anti-diabetic, antihypertensive or lipid-lowering treatment intensification, end of study/ leaving CPRD practice/ death  **Analysis:** summary statistics will be compared by ethnicity using Kaplan-Meier time-to-event analysis (cumulative failure probability in a given time frame by ethnicity, comparison by log-rank test), Cox proportional hazards regression models will be used to adjust for confounding/ investigate potential mediators  **Example effect measures**: cumulative failure probability for each ethnic group 1 year post detection of sub-optimal value, comparison of median survival times using the log-rank test by ethnicity, HRs of intensification stratified by year   1. **Outcome:** influence of deprivation, polypharmacy, multi-morbidity, drug adherence (iv) and v) only), patient engagement with healthcare, diabetes duration and HbA1c on any ethnic differences found in i) to v).   **Entry:** as above for i), iv) and v)  **Exit:** as above for i), iv) and v)  **Analysis:** as above for i) to v)  **Covariates:** smoking status, BMI, deprivation, polypharmacy, multi-morbidity, drug adherence, patient engagement with healthcare, diabetes duration and HbA1c  **Effect measures:** we will observe the degree of attenuation of the ethnic difference effect measure for each outcome when each covariate is inserted in turn to models, and thus evaluate the likelihood of confounding or mediation  Sub-group/ sensitivity analyses  Analyses will be stratified by age group, and changes in guidelines and temporal trends in drug usage will be accounted for by stratifying by time.  Three sensitivity analyses will be performed – all to boost ethnicity ascertainment. Firstly, on the 58% of CPRD participants with linked secondary care data from Hospital Episode Statistics (HES) (19), using ethnicity codes from HES - present in 78% (18). Secondly, on new registrants since 2006, 78% of whom have ethnicity codes recorded (19). Thirdly, on patients from practices in the London area; where previous research indicates ethnicity recording to be approaching 60% (28).  Plans for addressing misclassification and bias  ­Plans for addressing misclassification of diabetes status and ethnicity are given above (see sections K and L respectively).  Selection bias may arise in ethnicity recording, i.e. greater recording for patients from ethnic minorities, and may also vary across time (with greater recording after the introduction of incentives in 2004) and geographically (greater in urban areas with more ethnic diversity (28) than rural ones with less). We plan to address this in three ways, as detailed above under the “sub-group/ sensitivity analyses” heading. NB/ we do acknowledge these approaches may in themselves introduce bias, and thus we will carefully compare baseline characteristics between the sensitivity and primary analysis populations.  Though primary care prescription data are almost entirely computerised, bias may be introduced into the descriptive arms of the study from unrecorded prescriptions issued in secondary care; equally medication started in secondary care and continued in primary care may falsely lengthen times to up-titration. We anticipate little bias in the monitoring outcome, where blood tests and clinical data such as blood pressure (including those recorded in secondary care) are generally automatically entered into notes via specific templates. Additionally, some bias may be present regarding the ascertainment of treatment intensification, where the intervention is non-pharmacological or constitutes referral to another service. Therefore we will also search for codes indicating lifestyle modification advice or referral which are chronologically close to the medication exposure period.  We plan to examine the influence of drug adherence in explaining any associations between ethnicity and prescribing, but this will only be measured by proxy using the proportion of days covered method; we are unable to verify whether prescriptions have been obtained from pharmacies or whether medication has been taken.  Multiple comparisons  As the study will involve multiple comparisons, findings will be interpreted with caution, and attention paid to clinically meaningful effect sizes rather than p values per se. |
| Plan for addressing confounding Age and sex will be adjusted for in all analyses as potential confounders of associations between ethnicity and prescribing. The potential mediating influences of smoking, BMI, polypharmacy, multimorbidity, patient adherence, patient engagement, diabetes duration and HbA1c will be quantified by adjusting regression models for each of these variables in turn. |
| Plans for addressing missing data Monitoring and treatment of type 2 diabetes is incentivised by the UK’s primary care Quality Outcomes Framework (QOF), therefore recording of cardiovascular risk factors and some other of the covariates mentioned above is likely to be more complete than for other disease areas. Where these factors are missing, we will use complete case analysis as data may not be missing at random.  Missing ethnicity data is the chief concern. Given that ethnicity data are unlikely to be missing at random, multiple imputation methods are not appropriate, and we will perform complete case analysis instead. Complete case analysis will be unbiased if missingness is independent of the outcome, conditional on model covariates. One of the main reasons that missingness would be expected to be associated with prescribing patterns is that people who are poorly engaged with their GP may be less likely to have ethnicity recorded (whatever their ethnicity), or be eligible to be prescribed medication. However, as discussed above we plan to adjust for number of consultations in the year prior to diagnosis. Within those with similar levels of GP contact prior to diabetes diagnosis, it is less conceivable that missingness of ethnicity will be strongly associated with prescribing patterns. Additionally, three sensitivity analyses (see section N) will be performed to boost ethnicity recording. |
| Patient or user group involvement (if applicable) A study steering group will be convened consisting of the collaborators and several people with type 2 diabetes, ideally representing each ethnic group. These individuals will be recruited from the pool of participants from the SABRE study (Southall and Brent Revisited, PI: NC) and will meet annually to discuss the study progress, results and dissemination of findings. Key results will be reported via the media, Diabetes UK website, Living with Diabetes events and “Balance” magazine (for Diabetes UK members), to make people with diabetes aware of the findings. |
| Plans for disseminating and communicating study results, including the presence or absence of any restrictions on the extent and timing of publication Results of this study will be communicated and disseminated through publication in peer-reviewed journals, conference proceedings (e.g. the corresponding author (SE) has funding for attendance at the Diabetes UK, European Association for the Study of Diabetes and International Society for Pharmaco-epidemiology annual meetings) and informal presentations in the applicants’ home institutions. Additionally, this work is being undertaken as part of the corresponding author’s (SE) PhD, so findings will be communicated via her thesis. There are no anticipated restrictions on the extent or timing of the publication of findings.  **Conflict of interest statement:** There are no conflicts of interest to declare. |
| Limitations of the study design, data sources, and analytic methods The main limitation is missingness of ethnicity data, though as explained above we do not anticipate that using a complete case analysis will induce serious bias, and we have planned sensitivity analyses to investigate this.  Other than age, it may be difficult to establish factors that might cause HbA1c, blood pressure and lipid targets to be laxer, e.g. frailty, patient preference. Furthermore, patient records are unlikely to contain information on factors such as language, education, health beliefs which may influence willingness to intensify or engage with treatment. |
| References 1.Tillin T, Hughes AD, Godsland IF, Whincup P, Forouhi NG, Welsh P, et al. Insulin resistance  and truncal obesity as important determinants of the greater incidence of diabetes in Indian  Asians and African Caribbeans compared with Europeans: the Southall And Brent REvisited  (SABRE) cohort. Diabetes care 2013; 36(2):383-93.  2. UK Prospective Diabetes Study (UKPDS) Group. Intensive blood-glucose control with  sulphonylureas or insulin compared with conventional treatment and risk of complications in  patients with type 2 diabetes (UKPDS 33). Lancet 1998; 352(9131):837-53.  3. Millett C, Gray J, Saxena S, Netuveli G, Khunti K, Majeed A. Ethnic disparities in diabetes  Management and pay-for-performance in the UK: the Wandsworth Prospective Diabetes Study.  PLoS medicine. 2007;4(6):e191.Epub 2007/06/15.  4. Alshamsan R, Majeed A, Vamos EP, Khunti K, Curcin V, Rawaf S, Millett C. Ethnic  differences in diabetes management in patients with and without comorbid medical conditions: a cross-sectional study. Diabetes Care 2011; 34(3):655-7.  5. Tillin T, Hughes AD, Mayet J, Whincup P, Sattar N, Forouhi NG, McKeigue PM, Chaturvedi N. The relationship between metabolic risk factors and incident cardiovascular disease in Europeans, South Asians, and African Caribbeans: SABRE (Southall and Brent Revisited) -- a prospective population-based study. J Am Coll Cardiol 2013; 61(17):1777-86.  6. Tight blood pressure control and risk of macrovascular and microvascular complications in  type 2 diabetes: UKPDS 38. UK Prospective Diabetes Study Group. BMJ (Clinical research ed).  1998;317(7160):703-13. Epub1998/09/11.  7. National Institute for Health and Clinical Excellence. Type 2 diabetes: The management of  type 2 diabetes. 2015 [cited 2018 15th November]; Clinical guideline 87].  8. Wilkinson S, Douglas I, Stirnadel-Farrant H, Fogarty D, Pokrajac A, Smeeth L, Tomlinson L.  Changing use of antidiabetic drugs in the UK: trends in prescribing 2000-2017.BMJ Open 2018;  8(7): e022768:doi:10.1136/bmjopen-2018-022768.  9. Wilkinson S, Douglas IJ, Williamson European, Stirnadel-Farrant HA, Fogarty D, Pokrajac A, Smeeth L, Tomlinson LA. Factors associated with choice of intensification treatment for type 2 diabetes after metformin monotherapy: a cohort study in UK primary care. Clinical epidemiology 2018; 10; 1639-1648.  10. James GD, Baker P, Badrick E, Mathur R, Hull S, Robson J. Type 2 diabetes: a cohort study  of treatment, ethnic and social group influences on glycated haemoglobin. BMJ open.2012;2(5).  Epub 2012/10/23.  11. Barrera L, Leaper C, Pape UJ, Majeed A, Blangiardo M, Millett C. Impact of ethnic-specific guidelines for anti-hypertensive prescribing in primary care in England: a longitudinal study.  BMC health services research. 2014;14:87. Epub 2014/02/27.  12. Fleetcroft R, Schofield P, Ashworth M. Variations in statin prescribing for primary  cardiovascular disease prevention: cross-sectional analysis. BMC health services research.  2014;14:414. Epub 2014/09/23.  13. Ashworth M, Lloyd D, Smith RS, Wagner A, Rowlands G. Social deprivation and statin  prescribing: a cross sectional analysis using data from the new UK general practitioner 'Quality  and Outcomes Framework'. Journal of public health (Oxford, England). 2007;29(1):40-7. Epub  2006/10/31.  14. Desai U, Kirson NY, Kim J, Khunti K, King S, Trieschman E, Hellstern M, Hunt PR,  Mukherjee J. Time to Treatment Intensification After Monotherapy Failure and Its Association  With Subsequent Glycemic Control Among 93,515 Patients With Type 2 Diabetes. Diabetes  Care. 2018; 41(10):2096-2104.  15. Sinnott SJ, Tomlinson LA, Root AA, Mathur R, Mansfield KE, Smeeth L, Douglas IJ.  Comparative effectiveness of fourth-line anti-hypertensive agents in resistant hypertension: A  systematic review and meta-analysis. Eur J Prev Cardiol 2017 Feb; 24(3):228-238.  16. Millett C, Gray J, Wall M, Majeed A. Ethnic disparities in coronary heart disease  management and pay for performance in the UK. J Gen Intern Med. 2009 Jan;24(1):8-13.  17. Danese MD, Gleeson M, Kutikova L, Griffiths RI, Khunti K, Seshasai SRK, Ray KK.  Management of lipid-lowering therapy in patients with cardiovascular events in the UK: a  retrospective cohort study. BMJ Open 2017; 7(5):e013851. doi: 10.1136/bmjopen-2016-013851.  18. Mathur R, Bhaskaran K, Chaturvedi N, Leon DA, vanStaa T, Grundy E, Smeeth L.  Completeness and usability of ethnicity data in UK-based primary care and hospital databases.  J Public Health (Oxf). 2014 Dec;36(4):684-92.  19. Herrett E, Gallagher AM, Bhaskaran K, Forbes H, Mathur R, van Staa T, Smeeth L. Data  Resource Profile: Clinical Practice Research Datalink (CPRD).Int J Epidemiol 2015;44(3):827-36  20. Office of National Statistics. 2011 census: Population estimates for the United Kingdom,  March 2011. 2012[cited 16th November 2018]; Available from:  http://www.ons.gov.uk/peoplepopulationandcommunity/populationandmigration/populationestimates/bulletins/2011 censuspopulationestimatesfortheunitedkingdom/2012-12-17.  21. Hamada S, Gulliford MC. Antidiabetic and cardiovascular drug utilisation in patients  diagnosed with type 2 diabetes mellitus over the age of 80 years: a population-based cohort  study. Age Ageing. 2015; 44(4):566-73.  22. Sinclair AJ, Alexander CM, Davies MJ, Zhao C, Mavros P. Factors associated with initiation of antihyperglycaemic medication in UK patients with newly diagnosed type 2 diabetes. BMC Endocr Disord 2012;12:1.  23. Khunti K, Wolden M, Thorsted B, Andersen M, Davies MJ. Clinical inertia in people with type  2 diabetes; a retrospective cohort study of more than 80,000 people. Diabetes care 2013; 36;  3411-3417.  24. National Institute for Health and Clinical Excellence. Hypertension in adults: diagnosis and  management. Clinical guideline [CG127]. Published August 2012. Cited 23/11/2018:  https://www.nice.org.uk/guidance/cg127/chapter/1-guidance  25. National Institute for Health and Clinical Excellence. Cardiovascular disease: risk  assessment and reduction, including lipid modification. Clinical guideline [CG181]. Published  July 2014. Cited 23/11/2018: https://www.nice.org.uk/guidance/cg181  26. Eastwood SV, Mathur R, Atkinson M, Brophy S, Sudlow C, Flaig R, de Lusignan S, Allen N, Chaturvedi N. Algorithms for the Capture and Adjudication of Prevalent and Incident Diabetes in UK Biobank. Published: September 15, 2016https://doi.org/10.1371/journal.pone.0162388  27. Choudhry NK, Shrank WH, Levin RL, Lee JL, Jan SA, Brookhart MA, et al. Measuring  Concurrent adherence to multiple related medications. The American journal of managed care.  2009;15(7):457-64. Epub 2009/07/11.  28. Tippu Z, Correa A, Liyanage H, Burleigh D, McGovern A, Van Vlymen J, Jones S, De Lusignan S. Ethnicity Recording in Primary Care Computerised Medical Record Systems: An Ontological approach. J Innov Health Inform. 2017 Mar 14;23(4):920. |
| List of Appendices Peer review process and outcome: Diabetes UK Sir George Alberti Clinical Training Fellowship award panel  Peer review process and outcome: LSHTM ethics board  Diagram of type 2 diabetes diagnosis adjudication algorithm for primary care records  Diabetes code list  Ethnicity code list  Medication for type 2 diabetes code list  Antihypertensive medication code list  Lipid-lowering medication code list  Covariates code lists:  coronary heart disease  cerebrovascular disease  heart failure  atrial fibrillation  hypertension  chronic kidney disease  peripheral arterial disease  retinopathy  neuropathy  cancer  COPD/ asthma  severe mental illness |

| **AMENDMENT – 9^TH^ DECEMBER 2019**  **(Highlighted text indicates new material)** |
| --- |
| Study Title (Max. 255 characters) Ethnic differences in the prescribing of anti-diabetic, antihypertensive and lipid-lowering medication for people with and without type 2 diabetes. |
| 1. **Lay summary (Max. 250 words)**     The UK’s South Asian and African Caribbean populations experience far higher rates of type 2 diabetes and its cardiovascular disease complications, e.g. heart disease and strokes, than European-origin (white) groups. Ethnic minority populations without diabetes also experience an excess of most types of cardiovascular disease. Control of blood glucose, high blood pressure and high cholesterol helps to reduce cardiovascular disease complications from diabetes. Despite this, we know little about ethnic differences in the use of medications which do this. Limited existing research suggests that both choice and effective use of diabetes, blood pressure or cholesterol-lowering medication may differ by ethnicity.  This study aims to compare use of these medications for people with and without diabetes of European, South Asian and African Caribbean origin. Using computerised medical records, the commencement, type, dose, monitoring and adjustment of medication will be studied, and reasons for differences sought.  By identifying ethnic disparities in the use of medications to control diabetes, high blood pressure and high cholesterol, we may highlight reasons for the excess of diabetes complications and cardiovascular disease seen in UK South Asian or African Caribbean groups. These findings may influence prescribing policies, and thus ultimately reduce ethnic differences in cardiovascular disease and complications of type 2 diabetes. |
| Technical Summary (Max. 300 words) UK South Asian and African Caribbean people with type 2 diabetes experience worse diabetic control and more cardiovascular complications than the European-origin population; ethnic minority populations without diabetes also experience an excess of most types of cardiovascular disease. Explanations are unclear. Blood pressure and lipid control, crucial to cardiovascular risk reduction for people with and without diabetes, also differ by ethnicity. However, ethnic differences in prescribing for diabetes, hypertension and hyperlipidaemia remain understudied.  Ethnic differences in prescribing anti-diabetic (in people with diabetes), antihypertensive and lipid-lowering medication (in people with and without type 2 diabetes) will be sought, including: i) time to commencement ii) choice of medication, iii) dosage, iv) monitoring and iv) time to intensification (either medication up-titration, addition or class switching) after detection of sub-optimal control.  Primary care electronic medical records will be used. Established algorithms and code lists will define ethnicity, diagnoses and medication use. Data will be analysed using Kaplan-Meier time-to-event methods and logistic, Poisson and Cox regression models (according to outcome), with South Asian or African Caribbean ethnicity as the exposure (baseline category=European). These methods will allow for differences in follow-up time. The potentially confounding or mediating influences of age, sex, smoking, BMI, deprivation, polypharmacy, multi-morbidity, drug adherence, patient engagement and diabetes duration/ HbA1c will be explored. |
| D. Outcomes to be Measured Proportion of people eligible for antihypertensive or lipid-lowering medication who are prescribed the respective medication; Time to first anti-diabetic/ antihypertensive/ lipid-lowering medication prescription; Choice of anti-diabetic/ antihypertensive/ lipid-lowering medication; Dose of anti-diabetic/ antihypertensive/ lipid-lowering medication; Monitoring of anti-diabetic/ antihypertensive/ lipid-lowering medication; Time to anti-diabetic/ antihypertensive/ lipid-lowering treatment intensification. |
| Objectives, Specific Aims and Rationale Overall aim  To investigate ethnic differences in prescribing for type 2 diabetes in UK primary care.  Objectives  For people of European, South Asian and African Caribbean origin, ethnic differences in the following will be investigated:   1. Proportions of people with: a) incident hypertension started on antihypertensives and b) an elevated 10 year CVD risk score (according to QRISK, ASSIGN, JBS2, Framingham or unspecified risk-estimating equations (26)) being commenced on statin treatment. 2. Time to initiation of first-line anti-diabetic/ antihypertensive/lipid-lowering medication 3. Choice of anti-diabetic (first- to third-line), antihypertensive and lipid-lowering medication 4. Doses used for each anti-diabetic, antihypertensive and lipid-lowering medication 5. Monitoring rate after medication commencement 6. Time to medication intensification (up-titration, addition or switching) after detection of sub-optimal control of diabetes, hypertension or blood lipids   Additionally, we will examine potential confounding or mediating role/s of:   1. Smoking, BMI, deprivation, polypharmacy, multi-morbidity, drug adherence (for objectives v) and vi)), patient engagement with healthcare, diabetes duration and HbA1c (in people with diabetes).   Null hypotheses   1. No ethnic difference in proportions of people started on a) antihypertensives or b) statin treatment will be detected. 2. No ethnic difference in time to initiation of first-line anti-diabetic, antihypertensive or lipid-lowering medication will be detected. 3. Regarding choice of medication: 4. No ethnic difference in the proportions of people prescribed metformin as first-line, or sulphonylureas, DPP4-inhibitors, thiazolidinediones, SGLT2 inhibitors, GLP1 agonists or insulin as second- or third-line diabetes treatment will be observed. 5. No ethnic difference in the proportions of people prescribed ACE inhibitors/ angiotensin-receptor blockers, calcium-channel blockers or thiazide diuretics for hypertension will be observed. 6. No ethnic difference in the proportions of people with ≥10% cardiovascular disease risk who are prescribed lipid-lowering medication will be observed. 7. No ethnic difference in the proportions prescribed the maximum dose of anti-diabetic, antihypertensive or lipid-lowering medication will be observed, and the modal dose for each class will not differ by ethnicity. 8. No ethnic difference in the monitoring rates of anti-diabetic, antihypertensive or lipid-lowering medication will be observed. 9. No ethnic difference in time to treatment intensification (after the detection of sub-optimal control) of anti-diabetic, antihypertensive or lipid-lowering medication will be observed. 10. Associations between ethnicity and prescribing will not be confounded or mediated by smoking, BMI, deprivation, polypharmacy, multi-morbidity, drug adherence, patient engagement, diabetes duration or HbA1c (in people with diabetes).   Rationale  Research indicates that control of diabetes and its allied cardiovascular risk factors is poorer in UK’s minority ethnic groups than in the background white population. Crucially, these discrepancies are likely to contribute to higher rates of heart disease and stroke, in people with and without diabetes, seen in these groups. By examining the associations above, this study seeks to determine whether ethnic differences in prescribing for people with and without diabetes are present, and explanations for them.  It is important to compare prescribing in people with and without diabetes for each ethnic group. Our previous work suggests that diabetes is a much more potent CVD risk factor in people of South Asian and African Caribbean-origin than those of European-origin, and differential prescribing of statins and anti-hypertensives in those with vs. without diabetes is a possible explanation for this.  If ethnic inequalities in prescribing are present, their identification may influence future prescribing recommendations and thus eventually lessen the personal and societal burden of diabetes and cardiovascular disease complications in ethnic minority groups. |
| Study Background The UK’s largest ethnic minority groups, people of South Asian and African Caribbean descent, are disproportionately affected by type 2 diabetes (1), and most types of cardiovascular disease (2). UKPDS (UK Prospective Study of Diabetes) established the importance of tight blood glucose control in reducing CVD complications (3), but research suggests that glucose control is poorer in UK South Asians and African Caribbeans than Europeans (4, 5), despite their greater prevalence of complications (2). Yet the use of anti-diabetic medication in these groups remains understudied. Blood pressure and lipid control is key in reducing CVD risk in people with and without diabetes (6), therefore we will also examine prescribing patterns for antihypertensives and lipid-lowering medication.  UK guidelines recommend metformin as first-line anti-diabetic therapy, with several choices of further agents before insulin use (7). A recent study has reported on the choice of second-line treatments in CPRD (8; ISAC protocol number 16_267), with some indication that people from ethnic minority groups are less likely to receive newer anti-diabetic drugs. However, the influence of key factors such as diabetes duration and adherence to treatment were not accounted for, and the related questions of monitoring frequency and expediency of appropriate treatment intensification were not studied. Moreover, evidence from smaller primary care databases suggests South Asians and African Caribbeans are less likely than Europeans to receive insulin, and more likely to be on a higher number of oral diabetes drugs (5, 9). UK hypertension guidelines advocate calcium-channel blockers (CCBs) as first-line treatment for African Caribbeans, and ethnic differences in monotherapy are established (10), but little examination of ethnic differences in prescribing exists beyond this. Studies from aggregated primary care data indicate that statins may be under-prescribed in areas with large ethnic minority populations (11, 12), but were limited by analysis at area level.  Delay in anti-diabetic treatment intensification is linked to poorer subsequent control (13), but in spite of the poorer diabetic control in ethnic minority groups, ethnic differences in time to intensification have never been studied. Equally, an absence of research on ethnic differences in intensification of antihypertensives exists (14). However, previous research indicates that African Caribbean people are less likely than their white counterparts to be prescribed statins (15), though it is unclear if this is the case for people with diabetes. Additionally, whilst evidence from CPRD indicates that hypercholesterolaemia with high cardiovascular risk is inadequately treated in people with diabetes (16), scrutiny by ethnicity is lacking. |
| Feasibility counts The primary analysis will be using data from CPRD alone (see section M for sensitivity analyses). Ethnicity recording for active patients is approximately 50% (17, 18). The ethnic breakdown is similar to that of the UK census 2011(20).  From 2000-2017, ≈280K patients initiated first-line and ≈80K second-line treatment for type 2 diabetes in CPRD (8). If 50% of these people had ethnicity codes present in their record, this would correspond to ≈140K starting first-line and ≈40K starting second line treatment. Using the 2011 census to calculate proportions, this would equate to ≈120.4K Europeans, ≈10.5K South Asians and ≈4.6K African Caribbeans initiating first-line treatment and 34.4K, 3.0K and 1.3K respectively starting second-line treatment. These are conservative estimates given the over-representation of ethnic minorities in the diabetic population.  Approximately 77% of the people with diabetes initiating first-line treatments are likely to be on antihypertensives (19), giving numbers for antihypertensive analyses of ≈92.7K Europeans, ≈8.1K South Asians, ≈3.5K African Caribbeans. Previous work in CPRD shows that ≈64% of people with type 2 diabetes receive statins (19), which will yield numbers for the lipid-lowering medication analyses of ≈77.1K Europeans, ≈6.7K South Asians and ≈2.9K African Caribbeans.  For people without diabetes, we expect approximately 219K people to initiate antihypertensive treatment over a similar study period to our own (20), equating to at least 94K Europeans, 6K South Asians and 3K African Caribbeans, once multipliers for ethnicity recording and the 2011 Census ethnicity breakdown are applied. From Chidwick et al (21), 113,035 people over 40 without a prior history of CVD had a high CVD risk score recorded between 2005-2013. Assuming 50% of these people had ethnicity recorded, and proportions by ethnicity are as per the 2011 census, this equates to approximately 49K Europeans, 3K South Asians and 2K African Caribbeans. |
| Sample size considerations Anti-diabetic analyses  Sample sizes as per “feasibility counts” section, see table 1(below) for detectable differences in ethnic difference effect sizes. Corresponding to objective i), the proportion prescribed an anti-diabetic at 1 year post-diagnosis (42%) is taken from Sinclair et al (22) (detectable differences calculated using stpower cox command in Stata). Corresponding to objective ii), proportions prescribed metformin as first-line (73%) and a sulphonylurea as second line treatment (87%) are taken from Wilkinson et al (8) (power twoproportions command). Corresponding to objectives iv) and v), mean monitoring rate (2.45 times per year [extrapolated], assuming a SD of 2) (power twomeans command) and probability of treatment intensification at 1 year (24%) are taken from Khunti et al (23) (stpower cox command).  **Table 1. Detectable ethnic differences in outcomes.**   \| **Outcome** \| **Exposure (ethnicity): N vs. N** \| **Detectable ethnic difference effect sizes at α=0.05, β=0.90** \| \| --- \| --- \| --- \| \| Time from diagnosis to first anti-diabetic prescription \| South Asians vs. Europeans:  10.5K vs. 120.4K \| HR: 1.03 \| \| African Caribbeans vs. Europeans:  4.6K vs. 120.4K \| HR: 1.03 \| \| % prescribed metformin first-line \| South Asians vs. Europeans:  10.5K vs. 120.4K \| Difference in proportions: 1.4% \| \| African Caribbeans vs. Europeans:  4.6K vs. 120.4K \| Difference in proportions: 2.1% \| \| % prescribed sulphonylurea second-line \| South Asians vs. Europeans:  3.0K vs. 34.4K \| Difference in proportions: 2.0% \| \| African Caribbeans vs. Europeans:  1.3K vs. 34.4K \| Difference in proportions: 2.9% \| \| Mean monitoring rate, measurements per person year at risk \| South Asians vs. Europeans:  10.5K vs. 120.4K \| Rate difference: 0.07 \| \| African Caribbeans vs. Europeans:  4.6K vs. 120.4K \| Rate difference: 0.10 \| \| Time from sub-optimal diabetes control to treatment intensification \| South Asians vs. Europeans:  10.5K vs. 120.4K \| HR: 1.04 \| \| African Caribbeans vs. Europeans:  4.6K vs. 120.4K \| HR: 1.04 \| \| Time to antihypertensive initiation \| South Asians vs. Europeans:  7.6K vs. 119.4K \| HR: 1.02 \| \| African Caribbeans vs. Europeans:  3.8K vs. 119.4K \| HR: 1.02 \| \| Proportions of eligible people at high recorded CVD risk receiving statins \| South Asians vs. Europeans:  3K vs. 49K \| HR: 1.15 \| \| African Caribbeans vs. Europeans:  2K vs. 49K \| HR: 1.18 \|   Antihypertensive analyses  Sample sizes as per “feasibility counts” section. Little prior data exists in this area, but we can assume a mean time to measurement of 1 month (with an assumed SD of 1 month), as per guidelines for re-measurement after intensification (24). This would give a detectable ethnic difference in time until first monitoring as 0.04 months for South Asians vs. Europeans (using the power twomeans command in Stata), and 0.06 months for African Caribbeans vs. Europeans. Data from Petersen et al (25) suggest that 21% of those eligible for treatment for hypertension in the UK remain untreated. Adding this proportion to the expected numbers commencing antihypertensives by ethnicity (see feasibility count section), we would expect 119.4K Europeans, 7.6K South Asians and 3.8K African Caribbeans to be eligible for time to antihypertensive initiation analyses.  Lipid-lowering medication analyses  Sample sizes as per “feasibility counts” section. Assuming a mean time to monitoring of 3 months (26) and an SD of 1 month, the detectable ethnic difference in time until first monitoring would be 0.04 months for South Asians vs. Europeans, and 0.06 months for African Caribbeans vs. Europeans (using the power twomeans command in Stata). Chidwick et al reported that 24% of the high risk population received statins (21), equating to detectable ethnic differences in proportions receiving statins of HRs 1.18 and 1.15.  All detectable differences fall below what is likely to be considered clinically significant. |
| L. Definition of the Study population All permanently-registered patients with research-standard data and incident type 2 diabetes Read codes from at least 1 year after 1^st^ January 2006 (start of follow-up; coinciding with when ethnicity was recorded for ≈80% of new registrations (17)) to 31^st^ December 2018 in the CPRD. The study will be restricted to incident cases to enable more precise measurement of treatment stage; people with prevalent type 2 diabetes Read codes prior to 2006 will be excluded.  Type 2 diabetes will be defined by a previously tested algorithm that uses Read codes, medication, blood test results and diabetes-specific process of care Read codes to adjudicate diabetes status (27) – see appendix. Specific exclusions will include: type 1 diabetes, gestational diabetes, non-specified diabetes diagnosed at ≤40 years in Europeans or ≤35 years in South Asians and African Caribbeans (reducing misclassified type 1 diabetes), and secondary diabetes (e.g. drug-induced).  In people without diabetes, those eligible for antihypertensive treatment will include people ≥40 years of age with incident: Read codes for hypertension, clinic BP of ≥160/100, ambulatory BP of ≥150/95 or clinic BP of ≥140/90 or ambulatory BP of ≥135/85 **plus either** 10 year CVD risk score exceeding 20% or 10% (for calendar years 2006-2013 and 2014-2019 respectively) **or** evidence of target organ disease (i.e. Read codes for left ventricular hypertrophy, chronic kidney disease, micro/ macroalbuminuria, or hypertensive retinopathy) (24). Those eligible for lipid-lowering medication will include people ≥40 years of age with an incident recording of CVD risk score ≥20% (if recorded between 2006 and 2013), or ≥10% (if recorded between 2014 and 2019), to account for guidelines changes (26)) during the study period. |
| N. Exposures, outcomes and covariates *Data sources are listed in square parentheses*  Exposures:  For all objectives, the exposure is either South Asian or African Caribbean ethnicity (defined as above), with European ethnicity as the baseline category. *[Primary care clinical records]*  Health Outcomes by study objective:   1. Proportion of those eligible for primary prevention who are prescribed a) antihypertensives and b) lipid-lowering medication *[Primary care clinical records, prescription drug files]* 2. Time to first anti-diabetic/ antihypertensive/ lipid-lowering medication prescription. *[Primary care clinical records, prescription drug files.]* 3. Choice of a) anti-diabetic (first- to third-line), b) antihypertensive and c) lipid-lowering medications, see appendix for code lists for each (derived from chapters of the British National Formulary). *[Prescription drug files.]* 4. Doses used for each a) anti-diabetic, b) antihypertensive and c) lipid-lowering medication. *[Prescription drug files.]* 5. Monitoring rate, stratified by the time since treatment initiation, e.g. the number of a) HbA1c, b) BP or c) lipid measures divided by the amount of person time, within strata of time since initiation (i.e. first year, second year since initiation etc). *[Prescription drug files, test records.]* 6. Time to treatment intensification after detection of sub-optimal risk factor control. *[Primary care clinical records, prescription drug files, test records.]* Sub-optimal control will be classified as follows:    1. Diabetes; HbA1c≥53mmol/mol (7.5%) (7)    2. Hypertension; blood pressure≥140/80 mmHg, or 130/80 mmHg in those with neuropathy/ nephropathy or retinopathy (24)    3. Lipids; <40% reduction in non-HDL cholesterol (25) 7. Influence of the following on associations between ethnicity and prescribing: smoking, BMI, deprivation, polypharmacy, multi-morbidity, drug adherence (for outcomes iv) and v)), patient engagement with healthcare, diabetes duration and HbA1c. *[Linked IMD data, primary care clinical records, prescription drug files, test records.]*   Covariates (all taken from last entry in medical notes prior to follow-up time commencing)  Age *[Primary care clinical records]*  Sex *[Primary care clinical records]*  Smoking status *[Primary care clinical records]*  BMI *[Primary care clinical records]*  Deprivation (using patient-level IMD scores, with sensitivity analyses using practice-level IMD scores) *[Primary care clinical records, linked deprivation data]*  Polypharmacy (number of medications) *[Prescription drug files]*  Multimorbidity (defined by presence or absence of the following key co-morbidities: coronary heart disease, cerebrovascular disease, heart failure, atrial fibrilliation, hypertension, chronic kidney disease, peripheral arterial disease, retinopathy, neuropathy, cancer [any], COPD/ asthma, severe mental illness) *[Primary care clinical records]*  Patient engagement/ access (using the number of consultations in the year preceding diabetes diagnosis as a proxy) *[Primary care clinical records]*  Patient adherence (using the proportion of days covered: PDC=days of medication supplied per x day period/ x (27)). This will be calculated separately for all medications issued over the follow-up time of interest, and then a mean PDC for all medications will be derived for each patient. Both measures are important as adherence may vary by drug. *[Prescription drug files]*  Disease parameters: baseline HbA1c/ blood pressure and number of years since diabetes diagnosis *[Primary care clinical records, test records.]* |
| O. Data/ Statistical Analysis For all parts of the study, the population will be people with and without new-onset type 2 diabetes active in CPRD from 2006 (see section K, above) and the exposure will be ethnicity (either South Asian vs. European [baseline] or African Caribbean vs. European [baseline], as detailed in section L, above). Covariates in base models will include age and sex, and further covariates will be considered individually, as outlined in section vi) below. Statistical analysis is further considered by objective:   1. **Outcome:** Proportion of those eligible for primary prevention who are prescribed a) antihypertensives and b) lipid-lowering medication   **Entry:** Date of incident a) hypertension Read code/ raised BP reading or b) elevated 10 year CVD risk score, at least 12 months after study start date of 1^st^ January 2005  **Exit:** First of; date of first antihypertensive/ lipid-lowering medication prescription (defined by the absence of a prescription for the medication in the previous 6 months’ records), end of study, leaving CPRD practice or death  **Analysis:** Proportions receiving antihypertensives/ statin will be compared by ethnicity, then logistic regression models will be used to adjust for confounding/ investigate potential mediators  **Example effect measure:** Odds ratio (OR) of receiving an antihypertensive/ lipid-lowering medication vs. not for each ethnic group of interest, stratified by time period since identification of hypertension/ elevated CVD risk   1. **Outcome**: time to initiation of first-line anti-diabetic/ antihypertensive/ statin, probability of receiving anti-diabetic/ antihypertensive/ statin at one year post-diagnosis/ identification of high CVD risk   **Entry**: date of type 2 diabetes diagnosis/ incident hypertension Read code or raised BP reading/ incident high 10 year CVD risk score recording  **Exit**: date of first anti-diabetic/ antihypertensive/ statin prescription (defined by the absence of a prescription for the medication in the previous 6 months’ records), or end of study/ leaving CPRD practice/ death  **Analysis**: summary statistics will be compared by ethnicity using Kaplan-Meier time-to-event analysis (cumulative failure probability in a given time frame by ethnicity, comparison of medians by log-rank test), Cox proportional hazards regression models will be used to adjust for confounding/ investigate potential mediators  **Example effect measure**: hazards ratio (HR) for anti-diabetic/ antihypertensive/ statin prescription   1. **Outcome:** proportions of patients prescribed medication from each anti-diabetic (stratified by first- to third-line treatment), antihypertensive and lipid-lowering class (defined by the absence of a prescription for the medication in the previous 6 months’ records)   **Sub-population:** a) all participants starting first- to third-line anti-diabetic treatment, b) all patients on antihypertensives, or c) all patients on lipid-lowering medication  **Analysis:** proportions receiving each drug will be compared by ethnicity, then logistic regression models will be used to adjust for confounding/ investigate potential mediators  **Example effect measures**: odds ratio (OR) of being prescribed metformin (=1) vs. non-metformin medication (=0) at first stage of treatment, OR of being prescribed sulphonylurea (=1) vs. non- sulphonylurea medication (=0) at second stage of treatment, OR of calcium-channel blocking antihypertensive prescription vs. non- calcium-channel blocking antihypertensive in those on antihypertensive treatment, OR of statin prescription vs. non-statin lipid-lowering medication prescription in those with >10% 10 year cardiovascular risk   1. **Outcome:** proportions of patients prescribed each dose of each anti-diabetic, antihypertensive and lipid-lowering medication, stratified by treatment stage (defined by the absence of a prescription for the medication in the previous 6 months’ records)   **Sub-population:** a) all participants on first- to third-line anti-diabetic treatment, b) all patients on antihypertensives, or c) all patients on lipid-lowering medication  **Analysis: :** proportions receiving each dose of each drug will be compared by ethnicity, then logistic regression models will be used to adjust for confounding/ investigate potential mediators  **Example effect measures**: OR of being prescribed maximum dose (2g daily) vs. sub-maximum dose of metformin, OR of being prescribed atorvastatin 10mg per day vs. higher doses (20mg, 40mg or 80mg)   1. **Outcome:** yearly monitoring rate post commencement of medication from each anti-diabetic (first- to third-line), antihypertensive and lipid-lowering class – i.e. number of times HbA1c/ blood pressure/ blood lipids measured per person-year of follow-up, stratified by year after commencement; may be multiple entry dates per person for different medications   **Entry:** first use of any medications of interest from 2006 onwards  **Exit:** first of; end of strata (e.g. 1 year after medication commencement), end of study/ leaving CPRD practice/ death  **Analysis:** summary statistics will be compared by ethnicity, Poisson regression modelling used to adjust for confounding/ investigate potential mediators  **Example effect measures**: yearly monitoring rate ratios (RRs)   1. **Outcome:** time to medication intensification (up-titration, addition or switching) after detection of sub-optimal control of diabetes, hypertension or blood lipids, or probability of intensification 1 year post-detection of sub-optimal control; may be multiple entry dates per person for different medications   **Entry:** date when sub-optimal risk factor control detected (see section M for how this is defined for each risk factor)  **Exit:** first of; date of anti-diabetic, antihypertensive or lipid-lowering treatment intensification, end of study/ leaving CPRD practice/ death  **Analysis:** summary statistics will be compared by ethnicity using Kaplan-Meier time-to-event analysis (cumulative failure probability in a given time frame by ethnicity, comparison by log-rank test), Cox proportional hazards regression models will be used to adjust for confounding/ investigate potential mediators  **Example effect measures**: cumulative failure probability for each ethnic group 1 year post detection of sub-optimal value, comparison of median survival times using the log-rank test by ethnicity, HRs of intensification stratified by year   1. **Outcome:** influence of deprivation, polypharmacy, multi-morbidity, drug adherence (iv) and v) only), patient engagement with healthcare, diabetes duration and HbA1c on any ethnic differences found in i) to v).   **Entry:** as above for i), iv) and v)  **Exit:** as above for i), iv) and v)  **Analysis:** as above for i) to v)  **Covariates:** smoking status, BMI, deprivation, polypharmacy, multi-morbidity, drug adherence, patient engagement with healthcare, diabetes duration and HbA1c  **Effect measures:** we will observe the degree of attenuation of the ethnic difference effect measure for each outcome when each covariate is inserted in turn to models, and thus evaluate the likelihood of confounding or mediation  Sub-group/ sensitivity analyses  See section O of original application  Plans for addressing misclassification and bias  See section O of original application  Multiple comparisons  See section O of original application |
| U. References  1. Tillin T, Hughes AD, Godsland IF, Whincup P, Forouhi NG, Welsh P, et al. Insulin resistance and truncal obesity as important determinants of the greater incidence of diabetes in Indian Asians and African Caribbeans compared with Europeans: the Southall And Brent REvisited (SABRE) cohort. Diabetes care 2013; 36(2):383-93. 2. Tillin T, Hughes AD, Mayet J, Whincup P, Sattar N, Forouhi NG, McKeigue PM, Chaturvedi N. The relationship between metabolic risk factors and incident cardiovascular disease in Europeans, South Asians, and African Caribbeans: SABRE (Southall and Brent Revisited) -- a prospective population-based study. *J Am Coll Cardiol* 2013; 61(17):1777-86. 3. UK Prospective Diabetes Study Group. Tight blood pressure control and risk of macrovascular and microvascular complications in type 2 diabetes: UKPDS 38. *BMJ* (Clinical research ed). 1998;317(7160):703-13. 4. Millett C, Gray J, Saxena S, Netuveli G, Khunti K, Majeed A. Ethnic disparities in diabetes management and pay-for-performance in the UK: the Wandsworth Prospective Diabetes Study. PLoS medicine 2007;4(6):e191.Epub 2007/06/15. 5. Alshamsan R, Majeed A, Vamos EP, Khunti K, Curcin V, Rawaf S, Millett C. Ethnic differences in diabetes management in patients with and without comorbid medical conditions: a cross-sectional study. Diabetes Care 2011; 34(3):655-7. 6. UK Prospective Diabetes Study Group. Tight blood pressure control and risk of macrovascular and microvascular complications in type 2 diabetes: UKPDS 38. BMJ (Clinical research ed). 1998;317(7160):703-13. 7. National Institute for Health and Clinical Excellence. Type 2 diabetes: The management of type 2 diabetes. 2015 [cited 2018 15th November]; Clinical guideline 87]. 8. Wilkinson S, Douglas IJ, Williamson European, Stirnadel-Farrant HA, Fogarty D, Pokrajac A, Smeeth L, Tomlinson LA. Factors associated with choice of intensification treatment for type 2 diabetes after metformin monotherapy: a cohort study in UK primary care. Clinical epidemiology 2018; 10; 1639-1648. 9. James GD, Baker P, Badrick E, Mathur R, Hull S, Robson J. Type 2 diabetes: a cohort study of treatment, ethnic and social group influences on glycated haemoglobin. BMJ open.2012;2(5). Epub 2012/10/23. 10. Barrera L, Leaper C, Pape UJ, Majeed A, Blangiardo M, Millett C. Impact of ethnic-specific guidelines for anti-hypertensive prescribing in primary care in England: a longitudinal study. BMC health services research. 2014;14:87. 11. Fleetcroft R, Schofield P, Ashworth M. Variations in statin prescribing for primary cardiovascular disease prevention: cross-sectional analysis. *BMC health services research* 2014;14:414. 12. Ashworth M, Lloyd D, Smith RS, Wagner A, Rowlands G. Social deprivation and statin prescribing: a cross sectional analysis using data from the new UK general practitioner 'Quality and Outcomes Framework'. *Journal of public health* (Oxford, England). 2007;29(1):40-7. 13. Desai U, Kirson NY, Kim J, Khunti K, King S, Trieschman E, Hellstern M, Hunt PR, Mukherjee J. Time to Treatment Intensification After Monotherapy Failure and Its Association With Subsequent Glycemic Control Among 93,515 Patients With Type 2 Diabetes. Diabetes Care. 2018; 41(10):2096-2104. 14. Sinnott SJ, Tomlinson LA, Root AA, Mathur R, Mansfield KE, Smeeth L, Douglas IJ. Comparative effectiveness of fourth-line anti-hypertensive agents in resistant hypertension: A systematic review and meta-analysis. Eur J Prev Cardiol 2017 Feb; 24(3):228-238. 15. Millett C, Gray J, Wall M, Majeed A. Ethnic disparities in coronary heart disease management and pay for performance in the UK. J Gen Intern Med. 2009 Jan;24(1):8-13. 16. Danese MD, Gleeson M, Kutikova L, Griffiths RI, Khunti K, Seshasai SRK, Ray KK. Management of lipid-lowering therapy in patients with cardiovascular events in the UK: a retrospective cohort study. BMJ Open 2017; 7(5):e013851. doi: 10.1136/bmjopen-2016-013851. 17. Mathur R, Bhaskaran K, Chaturvedi N, Leon DA, vanStaa T, Grundy E, Smeeth L. Completeness and usability of ethnicity data in UK-based primary care and hospital databases. *J Public Health* (Oxf). 2014 Dec;36(4):684-92. 18. Herrett E, Gallagher AM, Bhaskaran K, Forbes H, Mathur R, van Staa T, Smeeth L. Data Resource Profile: Clinical Practice Research Datalink (CPRD). *Int J Epidemiol* 2015;44(3):827-36. 19. Hamada S, Gulliford MC. Antidiabetic and cardiovascular drug utilisation in patients diagnosed with type 2 diabetes mellitus over the age of 80 years: a population-based cohort study. Age Ageing. 2015; 44(4):566-73. 20. Mahmoudpour SH, Asselbergs FW, Souverein PC, de Boer A, Maitland-van der Zee AH. Prescription patterns of angiotensin-converting enzyme inhibitors for various indications: A UK population-based study. *Br J Clin Pharmacol* 2018;84(10):2365-2372. 21. Chidwick K, Strongman H, Matthews A, Stanway S, Lyon AR, Smeeth L, Bhaskaran K. Statin use in cancer survivors versus the general population: cohort study using primary care data from the UK clinical practice research datalink. *BMC Cancer* 2018 Oct 22;18(1):1018. 22. Sinclair AJ, Alexander CM, Davies MJ, Zhao C, Mavros P. Factors associated with initiation of antihyperglycaemic medication in UK patients with newly diagnosed type 2 diabetes. BMC Endocr Disord 2012;12:1. 23. Khunti K, Wolden M, Thorsted B, Andersen M, Davies MJ. Clinical inertia in people with type 2 diabetes; a retrospective cohort study of more than 80,000 people. Diabetes care 2013; 36; 3411-3417. 24. National Institute for Health and Clinical Excellence. Hypertension in adults: diagnosis and management. NICE guideline [NG136]. Published August 2019. Cited 5/11/2019: <https://www.nice.org.uk/guidance/ng136>. 25. Petersen J, Benzeval M. Untreated hypertension in the UK household population - Who are missed by the general health checks? *Prev Med Rep* 2016;4:81-6. 26. National Institute for Health and Clinical Excellence. Cardiovascular disease: risk assessment and reduction, including lipid modification. Clinical guideline [CG181]. Published July 2014. Cited 23/11/2018: <https://www.nice.org.uk/guidance/cg181> 27. Eastwood SV, Mathur R, Atkinson M, Brophy S, Sudlow C, Flaig R, de Lusignan S, Allen N, Chaturvedi N. Algorithms for the Capture and Adjudication of Prevalent and Incident Diabetes in UK Biobank. Published: September 15, 2016https://doi.org/10.1371/journal.pone.0162388 |
