# Supplementary material for: Ethnic differences in guideline-indicated statin initiation for people with type 2 diabetes in UK primary care, 2006–2019: A cohort study
Source: PLoS Med. 2021 Jun 29;18(6):e1003672. doi: 10.1371/journal.pmed.1003672 (PMC8241069; doi:10.1371/journal.pmed.1003672)
Supplement: S1 Table — (DOCX) [file pmed.1003672.s007.docx]

**Table S1. Comparison of baseline characteristics of people included in the complete case analysis vs. those excluded from analyses due to missing data.** Data are n (%), means±SD or median (IQR).

|  | **Included in complete case analysis** | **Excluded from complete case analysis** |
| --- | --- | --- |
| **N** | 31,039 (67) | 15,256 (33) |
| **Ethnicity** | *European*: 27,511 (88.6)  *South Asian:* 2,386 (7.7)  *African Caribbean:* 1,142 (3.7) | *European:* 13,780 (90.3)  *South Asian:* 1,059 (6.9)  *African Caribbean:* 417 (2.7) |
| **Age, yrs** | 58±13 | 57±15 |
| **Gender**  Male  Female | 17,145 (55)  13,894 (45) | 8,244 (54)  7,012 (46) |
| **Practice location**  Non-London  London | 26,768 (86)  4,271 (14) | 13,587 (89)  1,669 (11) |
| **Deprivation, practice IMD quintile**  1 (least deprived)  2  3  4  5 (most deprived) | 4,015 (13)  5,108 (16)  5,817 (19)  7,178 (23)  8,921 (29) | 1,773 (12)  2,327 (15)  2,967 (19)  3,774 (25)  4,415 (29) |
| **Smoking**  Never  Ex  Current | 12,004 (39)  13,638 (44)  5,397 (17) | 6,436 (42)  5,705 (37)  3,094 (20) |
| **Number of consultations in previous year** | 7 (4-12) | 6 (3-11) |
| **CKD Read code** | 1,149 (4) | 445 (3) |
| **Cancer** | 2,006 (6) | 1,123 (7) |
| **Asthma/ COPD** | 4,613 (15) | 1,914 (13) |
| **Serious mental illness** | 1,542 (5) | 590 (4) |
| **Number of medications prescribed in previous year** | 6 (3-10) | 5 (2-9) |
| **Antihypertensive use** | 13,613 (44) | 4,433 (29) |
| **If not on statin, recorded reason for declinature*** | 3,515 (11) | 1,624 (11) |
| **Exception reported from diabetes QOF** | 1,342 (4) | 1,070 (7) |
| **Time period of index date, by NICE guideline**  1^st^ Jan 2006 to 31^st^ May 2008  1^st^ Jun 2008 to 31^st^ Jul 2014  1^st^ Aug 2014 to 30^th^ Jun 2019 | 6,089 (20)  20,496 (66)  4,454 (14) | 4,664 (31)  9,702 (64)  890 (6) |
| **Incident statin use** | 20,897 (67) | 10,396 (68) |

*IMD= Index of Multiple Deprivation, CKD=chronic kidney disease, COPD=chronic obstructive pulmonary disease, QOF=quality outcomes framework, NICE=National Institute of Health and Clinical Excellence,*

**Reasons included: contraindicated, not tolerated, declined, or patient buying from pharmacy.*
