# Supplementary material for: Ethnic differences in guideline-indicated statin initiation for people with type 2 diabetes in UK primary care, 2006–2019: A cohort study
Source: PLoS Med. 2021 Jun 29;18(6):e1003672. doi: 10.1371/journal.pmed.1003672 (PMC8241069; doi:10.1371/journal.pmed.1003672)
Supplement: S2 Table — (DOCX) [file pmed.1003672.s008.docx]

**Table S2. Baseline characteristics by TC/ HDL ratio tertile for people of African/ African Caribbean ethnicity.** Data are mean±SD, n(%) or median (IQR).

|  | **Tertile 1**  **TC/HDL≤3.74** | **Tertile 2**  **TC/HDL>3.74 &≤4.76** | **Tertile 3**  **TC/HDL>4.76** |
| --- | --- | --- | --- |
| **N** | 378 | 381 | 383 |
| **Age** | 55±11 | 53±11 | 50±10 |
| **Male gender** | 166 (44) | 191 (50) | 232 (61) |
| **London practice** | 241 (64) | 245 (64) | 216 (56) |
| **Most deprived quintile of practice IMD** | 148 (39) | 158 (41) | 140 (37) |
| **Current smoking** | 37 (10) | 37 (10) | 53 (14) |
| **Number of consultations in previous year** | 7 (4-11) | 6 (3-10) | 6 (3-10) |
| **BMI** | 31±6 | 32±6 | 33±6 |
| **1+ comorbidity of CKD/ asthma or COPD/ cancer/ serious mental illness** | 88 (23) | 91 (24) | 72 (19) |
| **Number of different medications prescribed in previous year** | 6 (3-10) | 5 (3-9) | 4 (2-8) |
| **On antihypertensive** | 213 (56) | 170 (45) | 133 (35) |

*TC/ HDL ratio= total cholesterol to high density lipoprotein cholesterol ratio, IMD=Index of Multiple Deprivation, BMI=body mass index, CKD=chronic kidney disease, COPD=chronic obstructive pulmonary disease*
