# Supplementary material for: Ethnic differences in guideline-indicated statin initiation for people with type 2 diabetes in UK primary care, 2006–2019: A cohort study
Source: PLoS Med. 2021 Jun 29;18(6):e1003672. doi: 10.1371/journal.pmed.1003672 (PMC8241069; doi:10.1371/journal.pmed.1003672)
Supplement: S3 Table — (DOCX) [file pmed.1003672.s009.docx]

**Table S3. Baseline characteristics by ethnic sub-group.** Data are mean±SD, n(%) or median (IQR).

| **European ethnicity** | | | | |
| --- | --- | --- | --- | --- |
|  | **British** | **Irish** | | **Other/ unspecified White** |
| **N** | 26,238 | 257 | | 1,016 |
| **Age** | 56±13 | 51±11 | | 49±13 |
| **Male gender** | 14,492 (55) | 153 (60) | | 604 (59) |
| **London practice** | 2,226 (8) | 78 (30) | | 346 (34) |
| **Most deprived quintile of practice IMD** | 7,405 (28) | 79 (31) | | 301 (30) |
| **Current smoking** | 4,666 (18) | 53 (21) | | 242 (24) |
| **Number of consultations in previous year** | 7 (4-12) | 7 (4-12) | | 6 (4-10) |
| **TC/ HDL** | 4.93±1.55 | 4.91±1.45 | | 5.01±1.69 |
| **BMI** | 34±7 | 33±7 | | 33±7 |
| **1+ comorbidity of CKD/ asthma or COPD/ cancer/ serious mental illness** | 10,562 (27) | 108 (31) | | 307 (22) |
| **Number of different medications prescribed in previous year** | 6 (3-10) | 6 (3-11) | | 5 (3-9) |
| **On antihypertensive** | 111,968 (46) | 109 (42) | | 400 (39) |
| **South Asian ethnicity** | | | | |
|  | **Indian** | **Pakistani** | **Bangladeshi** | **Other/ unspecified South Asian** |
| **N** | 932 | 628 | 213 | 612 |
| **Age** | 51±11 | 49±11 | 45±10 | 50±11 |
| **Male gender** | 532 (57) | 341 (54) | 117 (55) | 316 (52) |
| **London practice** | 430 (46) | 111(18) | 50 (23) | 327 (53) |
| **Most deprived quintile of practice IMD** | 177 (19) | 316 (50) | 99 (47) | 98 (16) |
| **Current smoking** | 84 (9) | 111(18) | 45 (21) | 69 (11) |
| **Number of consultations in previous year** | 6 (3-10) | 7 (4-12) | 7 (4-12) | 6 (4-10) |
| **TC/ HDL** | 4.84±1.39 | 5.08±1.49 | 5.34±1.48 | 4.85±1.35 |
| **BMI** | 30±5 | 31±6 | 28±4 | 29±5 |
| **1+ comorbidity of CKD/ asthma or COPD/ cancer/ serious mental illness** | 222 (16) | 168 (19) | 49 (15) | 135 (15) |
| **Number of different medications prescribed in previous year** | 5 (2-8) | 7 (3-12) | 6 (4-12) | 5 (2-8) |
| **On antihypertensive** | 251 (27) | 137 (22) | 48 (22) | 184 (30) |
| **African/ African Caribbean ethnicity** | | | | |
|  | **Caribbean** | **African** | | **Other/ unspecified Black** |
| **N** | 420 | 553 | | 170 |
| **Age** | 56±11 | 50±10 | | 50±10 |
| **Male gender** | 187 (45) | 308 (56) | | 95 (56) |
| **London practice** | 259 (62) | 348 (63) | | 96 (56) |
| **Most deprived quintile of practice IMD** | 155 (37) | 232 (42) | | 59 (35) |
| **Current smoking** | 55 (13) | 33 (6) | | 39 (23) |
| **Number of consultations in previous year** | 7 (4-12) | 6 (3-9) | | 7 (3-11) |
| **TC/ HDL** | 4.29±1.36 | 4.43±1.35 | | 4.55±1.25 |
| **BMI** | 32±6 | 32±6 | | 33±7 |
| **1+ comorbidity of CKD/ asthma or COPD/ cancer/ serious mental illness** | 151 (27) | 110 (14) | | 63 (26) |
| **Number of different medications prescribed in previous year** | 5 (3-10) | 4 (2-8) | | 6 (3-9) |
| **On antihypertensive** | 207 (49) | 245 (44) | | 64 (38) |

*IMD= Index of Multiple Deprivation, TC/HDL= total cholesterol/ high density lipoprotein cholesterol ratio, BMI=body mass index, CKD=chronic kidney disease COPD=chronic obstructive pulmonary disease,*
