# Supplementary material for: Ethnic differences in guideline-indicated statin initiation for people with type 2 diabetes in UK primary care, 2006–2019: A cohort study
Source: PLoS Med. 2021 Jun 29;18(6):e1003672. doi: 10.1371/journal.pmed.1003672 (PMC8241069; doi:10.1371/journal.pmed.1003672)
Supplement: S4 Table — (DOCX) [file pmed.1003672.s010.docx]

**Table S4. Baseline characteristics of people with incident type 2 diabetes after 2006 with no prior statin use or ASCVD, by ethnicity, including missing ethnicity (complete case analysis).** Data are n (age-standardised %), age-adjusted means±SD or median (IQR).

|  | **European ethnicity** | **South Asian ethnicity** | **African/ African Caribbean**  **ethnicity** | **Missing ethnicity** |
| --- | --- | --- | --- | --- |
| **N** | 27,511 (48) | 2,386 (4) | 1,142 (2) | 25,847 (45) |
| **Ethnic sub-group** | British: 26,238 (95)  Irish: 257 (1)  Other/ not stated white: 1,016 (4) | Indian: 932 (39)  Pakistani: 628 (26)  Bangladeshi: 213 (9)  Other/ not stated South Asian: 612(26) | Caribbean: 420 (37)  African: 553 (48)  Other/ not stated black: 170 (15) | - |
| **Age, yrs** | 59±12 | 50±11 | 52±11 | 59±12 |
| **Age group**  ≤45 years  >45 to ≤65 years  >65 years | 4,009 (15)  15,505 (56)  7,997 (29) | 888 (37)  1,276 (53)  222 (9) | 307 (27)  679 (59)  156 (14) | 3,959 (15)  14,362 (56)  7,526 (29) |
| **Gender**  Male  Female | 15,249 (53)  12,262 (47) | 1,307 (53)  1,079 (47) | 589 (51)  553 (49) | 14,172 (53)  11,675 (47) |
| **Practice location**  Non-London  London | 24,861 (91)  2,650 (9) | 1,467 (66)  919 (34) | 440 (44)  702 (56) | 24,733 (96)  1,114 (4) |
| **Country**  England  Scotland  Wales  Northern Ireland | 19,205 (68)  5,464 (20)  2,540 (10)  302 (2) | 2,163 (90)  128(4)  93(5)  2 (0.01) | 1,093 (95)  25 (2)  21 (3)  3 (0.01) | 15,688 (60)  2,615 (9)  5.725 (22)  1,819 (8) |
| **Deprivation, practice IMD quintile**  1 (least deprived)  2  3  4  5 (most deprived) | 3,766 (12)  4,713 (15)  5,130 (18)  6,118 (22)  7,784 (32) | 203 (7)  287 (11)  505 (19)  700 (26)  691 (37) | 46 (3)  108 (10)  182 (15)  360 (30)  446 (42) | 4,854 (17)  3,985 (15)  5,311 (20)  5,351 (21)  6,346 (27) |
| **Smoking**  Never  Ex  Current | 9,829 (38)  12,721 (39)  4,961 (23) | 1,510 (61)  567 (23)  309 (16) | 665 (58)  350 (29)  127 (13) | 10,379 (42)  11,206 (37)  4,262 (22) |
| **Number of consultations in previous year:**  **Median (IQR)**  **Quartiles**  1 (0 to 3)  2 (4 to 7)  3 (8 to 12)  4 (13 +) | 7 (4-12)  5,901 (21)  8,540 (29)  6,702 (24)  6,368 (25) | 6 (3-10)  607 (27)  772 (29)  577 (29)  430 (16) | 6 (3-10)  309 (24)  373 (28)  250 (19)  210 (29) | 6 (3-11)  5,674 (22)  6,723 (25)  7,240 (28)  6,210 (26) |
| **Total: HDL cholesterol** | 4.96±1.59 | 4.67±1.45 | 4.21±1.43 | 4.93±1.52 |
| **Non-HDL cholesterol, mmol/l** | 4.38±1.18 | 4.16±1.04 | 3.97±1.12 | 4.36±1.19 |
| **HbA_1c_:**  %  mmol/mol | 7.8±2.1  61±23 | 7.6±2.0  59±23 | 7.8±2.3  60±25 | 7.9±2.1  62±24 |
| **BMI, kg/m^2^**  **Underweight/ Normal**  **Overweight**  **Obese** | 34±7  2,262 (9)  7,074 (21)  18,175 (71) | 28±6  183 (12)  679 (25)  1,524 (63) | 31±6  34 (5)  205 (17)  903 (78) | 33±7  2,279 (8)  6,735 (22)  16,833 (70) |
| **CKD Read code** | 1,058 (4) | 41 (3) | 50 (6) | 1, 334 (5) |
| **Cancer** | 1,902 (6) | 48 (2) | 56 (6) | 1,794 (5) |
| **Asthma/ COPD** | 4,175 (17) | 307 (17) | 131 (12) | 5,721 (24) |
| **Serious mental illness** | 1,405 (7) | 74 (4) | 63 (7) | 1,181 (6) |
| **Number of different medications prescribed in previous year:**  **Median (IQR)**  **Quartiles**  1 (0 to 2 medications)  2 (3 to 5 medications)  3 (6 to 9 medications)  4 (10 + medications) | 6 (3-10)  5,527 (20)  7,607 (27)  6,929 (25)  7,448 (28) | 5 (2-9)  605 (22)  620 (22)  552 (29)  609 (28) | 5 (2-9)  287 (23)  333 (25)  267 (21)  255 (31) | 5(3-10)  7,970 (31)  6,761 (26)  6,943 (27)  4,173 (17) |
| **Antihypertensive use** | 12,477 (36) | 620 (29) | 516 (41) | 12,339 (37) |
| **Recorded reason for declinature (if statin not prescribed)** | 1,181/8,708 (10) | 66/897 (6) | 30/537 (4) | 960 (9) |
| **Exception reported from diabetes Quality Outcomes Framework** | 1,186 (6) | 102 (7) | 54 (5) | 2,899 (12) |
| **Time period of index date, by NICE guideline**  1^st^ Jan 2006 to 31^st^ May 2008  1^st^ Jun 2008 to 31^st^ Jul 2014  1^st^ Aug 2014 to 30^th^ Jun 2019 | 5,583 (20)  17,849 (65)  4,079 (15) | 339 (14)  1,791 (75)  256 (11) | 167 (15)  856 (75)  119 (10) | 7,435 (29)  16,913 (65)  1,499 (6) |

*ASCVD=atherosclerotic cardiovascular disease, IMD=index of multiple deprivation, TC/HDL= total cholesterol/ high density lipoprotein cholesterol ratio, HbA_1c_= glycosylated haemoglobin A_1c_, BMI=body mass index, CKD=chronic kidney disease, COPD=chronic obstructive pulmonary disease, NICE=National Institute of Health and Clinical Excellence*
