# Supplementary material for: Ethnic differences in guideline-indicated statin initiation for people with type 2 diabetes in UK primary care, 2006–2019: A cohort study
Source: PLoS Med. 2021 Jun 29;18(6):e1003672. doi: 10.1371/journal.pmed.1003672 (PMC8241069; doi:10.1371/journal.pmed.1003672)
Supplement: S5 Table — (DOCX) [file pmed.1003672.s011.docx]

**Table S5. Associations between ethnicity and guideline-indicated statin initiation after type 2 diabetes diagnosis: missing vs. European ethnicity.** Data are HRs (95% CI), from multi-level models accounting for intra-practice clustering.

| **Model factors** | **European ethnicity** | **Missing ethnicity** |
| --- | --- | --- |
| Age + gender | 1 | 0.99 (0.94,1.03) |
| Age + gender + IMD | 1 | 0.99 (0.95,1.03) |
| Age + gender + smoking | 1 | 0.99 (0.95,1.03) |
| Age + gender + healthcare usage | 1 | 0.99 (0.95,1.03) |
| Age + gender + TC/ HDL | 1 | 0.99 (0.94,1.03) |
| Age + gender + BMI | 1 | 0.99 (0.94,1.03) |
| Age + gender + comorbidity | 1 | 0.99 (0.95,1.03) |
| Age + gender + polypharmacy + antihypertensive use | 1 | 0.99 (0.95,1.03) |
| All above covariates | 1 | 1.00 (0.95,1.04) |

*IMD=index of multiple deprivation, TC/HDL= total cholesterol/ high density lipoprotein cholesterol ratio, BMI=body mass index*
