# Supplementary material for: Ethnic differences in guideline-indicated statin initiation for people with type 2 diabetes in UK primary care, 2006–2019: A cohort study
Source: PLoS Med. 2021 Jun 29;18(6):e1003672. doi: 10.1371/journal.pmed.1003672 (PMC8241069; doi:10.1371/journal.pmed.1003672)
Supplement: S1 Text — (DOCX) [file pmed.1003672.s012.docx]

**Text S1. List of ethnicity Read codes.**

| **Read code** | **Read term** | **Ethnicity:**  **3 main groups** | **Ethnicity:  10 sub-groups** | **N** |
| --- | --- | --- | --- | --- |
| 9t00.00 | White:eng/welsh/scot/ni/brit - england and wales 2011 census | European | British | 11 |
| 9i00.00 | White british - ethnic category 2001 census | European | British | 2425 |
| 9i24.00 | Northern irish - ethnic category 2001 census | European | British | 138 |
| 9i0..00 | British or mixed british - ethnic category 2001 census | European | British | 8042 |
| 9i21.00 | Scottish - ethnic category 2001 census | European | British | 335 |
| 9S13.00 | White scottish | European | British | 3585 |
| 9i23.00 | Cornish - ethnic category 2001 census | European | British | 1 |
| 9S1..00 | White | European | British | 1837 |
| 9i22.00 | Welsh - ethnic category 2001 census | European | British | 254 |
| 9i25.00 | Ulster scots - ethnic category 2001 census | European | British | 1 |
| 9S10.00 | White british | European | British | 8702 |
| 9i20.00 | English - ethnic category 2001 census | European | British | 827 |
| 134B.00 | Race: caucasian | European | British | 25 |
| 9t20.00 | White: scottish - scotland ethnic category 2011 census | European | British | 2 |
| 9S14.00 | Other white british ethnic group | European | British | 53 |
| 9i1..00 | Irish - ethnic category 2001 census | European | Irish | 127 |
| 9S11.00 | White irish | European | Irish | 115 |
| 9i10.00 | White irish - ethnic category 2001 census | European | Irish | 15 |
| 9i2L.00 | Bosnian - ethnic category 2001 census | European | Other white | 1 |
| 9i2R.00 | Oth white european/european unsp/mixed european 2001 census | European | Other white | 59 |
| 9i2D.00 | Traveller - ethnic category 2001 census | European | Other white | 1 |
| 9i2H.00 | Commonwealth (russian) indep states - ethn categ 2001 census | European | Other white | 1 |
| 9i2F.00 | Polish - ethnic category 2001 census | European | Other white | 57 |
| 9i27.00 | Greek - ethnic category 2001 census | European | Other white | 8 |
| 9i2B.00 | Italian - ethnic category 2001 census | European | Other white | 29 |
| 9SAB.11 | Turkish (nmo) | European | Other white | 15 |
| 9i2G.00 | Baltic estonian/latvian/lithuanian - ethn categ 2001 census | European | Other white | 7 |
| 9SAA.00 | Greek/greek cypriot (nmo) | European | Other white | 1 |
| 9SAB.12 | Turkish cypriot (nmo) | European | Other white | 2 |
| 9i26.00 | Cypriot (part not stated) - ethnic category 2001 census | European | Other white | 3 |
| 9i2Q.00 | Mixed irish and other white - ethnic category 2001 census | European | Other white | 3 |
| 9SAA.12 | Greek cypriot (nmo) | European | Other white | 4 |
| 1341.00 | European origin | European | Other white | 21 |
| 9i2A.00 | Turkish cypriot - ethnic category 2001 census | European | Other white | 21 |
| 9i28.00 | Greek cypriot - ethnic category 2001 census | European | Other white | 20 |
| 9SAC.00 | Other european (nmo) | European | Other white | 6 |
| 9i2E.00 | Gypsy/romany - ethnic category 2001 census | European | Other white | 4 |
| 134N.00 | Race: white | European | Other white | 63 |
| 9i2K.00 | Albanian - ethnic category 2001 census | European | Other white | 3 |
| 9i2..00 | Other white background - ethnic category 2001 census | European | Other white | 365 |
| 9S12.00 | Other white ethnic group | European | Other white | 217 |
| 9SAA.11 | Greek (nmo) | European | Other white | 3 |
| 9i2J.00 | Kosovan - ethnic category 2001 census | European | Other white | 4 |
| 9i2S.00 | Other mixed white - ethnic category 2001 census | European | Other white | 5 |
| 9i29.00 | Turkish - ethnic category 2001 census | European | Other white | 64 |
| 9i2P.00 | Other republics former yugoslavia - ethnic categ 2001 census | European | Other white | 3 |
| 9i2T.00 | Other white or white unspecified ethnic category 2001 census | European | Other white | 20 |
| 9i2C.00 | Irish traveller - ethnic category 2001 census | European | Other white | 1 |
| 9SAB.00 | Turkish/turkish cypriot (nmo) | European | Other white | 5 |
| 9S43.11 | Black north african | European | Other black | 3 |
| 9i7..00 | Indian or british indian - ethnic category 2001 census | South Asian | Indian | 526 |
| 9S6..00 | Indian | South Asian | Indian | 378 |
| 1347.00 | Indian origin | South Asian | Indian | 7 |
| 9T1D.00 | Indian | South Asian | Indian | 21 |
| 9S7..00 | Pakistani | South Asian | Pakistani | 220 |
| 9i8..00 | Pakistani or british pakistani - ethnic category 2001 census | South Asian | Pakistani | 404 |
| 134M.00 | Race: pakistani | South Asian | Pakistani | 4 |
| 9i9..00 | Bangladeshi or british bangladeshi - ethn categ 2001 census | South Asian | Bangladeshi | 140 |
| 134I.00 | Race: bangladeshi | South Asian | Bangladeshi | 3 |
| 9S8..00 | Bangladeshi | South Asian | Bangladeshi | 70 |
| 9iA7.00 | Caribbean asian - ethnic category 2001 census | South Asian | Other South Asian | 4 |
| 9iA4.00 | Sri lankan - ethnic category 2001 census | South Asian | Other South Asian | 58 |
| 9SA6.11 | East african asian (nmo) | South Asian | Other South Asian | 3 |
| 9SA7.00 | Indian sub-continent (nmo) | South Asian | Other South Asian | 4 |
| 9iA1.00 | Punjabi - ethnic category 2001 census | South Asian | Other South Asian | 34 |
| 9iA..00 | Other asian background - ethnic category 2001 census | South Asian | Other South Asian | 215 |
| 9iA8.00 | British asian - ethnic category 2001 census | South Asian | Other South Asian | 37 |
| 9SH..00 | Other asian ethnic group | South Asian | Other South Asian | 143 |
| 9SA8.00 | Other asian (nmo) | South Asian | Other South Asian | 46 |
| 9iA2.00 | Kashmiri - ethnic category 2001 census | South Asian | Other South Asian | 1 |
| 9iAA.00 | Other asian or asian unspecified ethnic category 2001 census | South Asian | Other South Asian | 43 |
| 9SA6.12 | Indo-caribbean (nmo) | South Asian | Other South Asian | 1 |
| 9iA5.00 | Tamil - ethnic category 2001 census | South Asian | Other South Asian | 7 |
| 9iA3.00 | East african asian - ethnic category 2001 census | South Asian | Other South Asian | 16 |
| 9S2..00 | Black caribbean | African/ African Caribbean | Caribbean | 183 |
| 9S42.11 | Black caribbean | African/ African Caribbean | Caribbean | 11 |
| 134K.00 | Race: west indian | African/ African Caribbean | Caribbean | 3 |
| 134H.00 | Race: afro-caribbean | African/ African Caribbean | Caribbean | 6 |
| 9S42.00 | Black caribbean/w.i./guyana | African/ African Caribbean | Caribbean | 1 |
| 9iB..00 | Caribbean - ethnic category 2001 census | African/ African Caribbean | Caribbean | 212 |
| 9S42.12 | Black west indian | African/ African Caribbean | Caribbean | 4 |
| 9iD0.00 | Somali - ethnic category 2001 census | African/ African Caribbean | African | 22 |
| 9S3..00 | Black african | African/ African Caribbean | African | 217 |
| 9SA5.00 | Other african countries (nmo) | African/ African Caribbean | African | 5 |
| 9S44.00 | Black - other african country | African/ African Caribbean | African | 6 |
| 1342.00 | African origin | African/ African Caribbean | African | 7 |
| 9iC..00 | African - ethnic category 2001 census | African/ African Caribbean | African | 257 |
| 9iD1.00 | Nigerian - ethnic category 2001 census | African/ African Caribbean | African | 39 |
| 9S4..00 | Black, other, non-mixed origin | African/ African Caribbean | Other black | 6 |
| 9S43.12 | Black arab | African/ African Caribbean | Other black | 1 |
| 9S48.00 | Black black - other | African/ African Caribbean | Other black | 1 |
| 9SA3.00 | Caribbean i./w.i./guyana (nmo) | African/ African Caribbean | Other black | 1 |
| 9iD4.00 | Other black or black unspecified ethnic category 2001 census | African/ African Caribbean | Other black | 2 |
| 9iD2.00 | Black british - ethnic category 2001 census | African/ African Caribbean | Other black | 39 |
| 9S5..00 | Black - other, mixed | African/ African Caribbean | Other black | 5 |
| 9S47.00 | Black - other asian | African/ African Caribbean | Other black | 3 |
| 9iD..00 | Other black background - ethnic category 2001 census | African/ African Caribbean | Other black | 45 |
| 9SG..00 | Other black ethnic group | African/ African Caribbean | Other black | 13 |
| 9S46.00 | Black indian sub-continent | African/ African Caribbean | Other black | 1 |
| 9S41.00 | Black british | African/ African Caribbean | Other black | 43 |
| 9iD3.00 | Mixed black - ethnic category 2001 census | African/ African Caribbean | Other black | 3 |
| 9S43.00 | Black n african/arab/iranian | African/ African Caribbean | Other black | 1 |
| 9S45.11 | Black east african asian | African/ African Caribbean | Other black | 3 |
