# Supplementary material for: Ethnic differences in guideline-indicated statin initiation for people with type 2 diabetes in UK primary care, 2006–2019: A cohort study
Source: PLoS Med. 2021 Jun 29;18(6):e1003672. doi: 10.1371/journal.pmed.1003672 (PMC8241069; doi:10.1371/journal.pmed.1003672)
Supplement: Attachment — Submitted filename: Response_to_reviewers.docx [file pmed.1003672.s013.docx]

***Dear Dr. Eastwood,***

***Thank you very much for submitting your manuscript "Ethnic differences in guideline-indicated statin initiation for people with type 2 diabetes: a longitudinal cohort study using the Clinical Practice Research Datalink, 2006-2019." (PMEDICINE-D-21-00613R1) for consideration at PLOS Medicine.***

***Your paper was evaluated by a senior editor and discussed among all the editors here. It was also sent to independent reviewers, including a statistical reviewer. The reviews are appended at the bottom of this email and any accompanying reviewer attachments can be seen via the link below:***

***In light of these reviews, I am afraid that we will not be able to accept the manuscript for publication in the journal in its current form, but we would like to consider a revised version that addresses the reviewers' and editors' comments. Obviously we cannot make any decision about publication until we have seen the revised manuscript and your response, and we plan to seek re-review by one or more of the reviewers.***

***In revising the manuscript for further consideration, your revisions should address the specific points made by each reviewer and the editors. Please also check the guidelines for revised papers at*** [***https://eur01.safelinks.protection.outlook.com/?url=http%3A%2F%2Fjournals.plos.org%2Fplosmedicine%2Fs%2Frevising-your-manuscript&amp;data=04%7C01%7C%7C80624884120849429ea008d8efb7134e%7C1faf88fea9984c5b93c9210a11d9a5c2%7C0%7C0%7C637522916894615243%7CUnknown%7CTWFpbGZsb3d8eyJWIjoiMC4wLjAwMDAiLCJQIjoiV2luMzIiLCJBTiI6Ik1haWwiLCJXVCI6Mn0%3D%7C1000&amp;sdata=4%2BTwK02whXhtTyGwLH2ZiqN1YDSmnHdvAIk1DdVXKnA%3D&amp;reserved=0***](https://eur01.safelinks.protection.outlook.com/?url=http%3A%2F%2Fjournals.plos.org%2Fplosmedicine%2Fs%2Frevising-your-manuscript&amp;data=04%7C01%7C%7C80624884120849429ea008d8efb7134e%7C1faf88fea9984c5b93c9210a11d9a5c2%7C0%7C0%7C637522916894615243%7CUnknown%7CTWFpbGZsb3d8eyJWIjoiMC4wLjAwMDAiLCJQIjoiV2luMzIiLCJBTiI6Ik1haWwiLCJXVCI6Mn0%3D%7C1000&amp;sdata=4%2BTwK02whXhtTyGwLH2ZiqN1YDSmnHdvAIk1DdVXKnA%3D&amp;reserved=0) ***for any that apply to your paper. In your rebuttal letter you should indicate your response to the reviewers' and editors' comments, the changes you have made in the manuscript, and include either an excerpt of the revised text or the location (eg: page and line number) where each change can be found. Please submit a clean version of the paper as the main article file; a version with changes marked should be uploaded as a marked up manuscript.***

***In addition, we request that you upload any figures associated with your paper as individual TIF or EPS files with 300dpi resolution at resubmission; please read our figure guidelines for more information on our requirements:*** [***https://eur01.safelinks.protection.outlook.com/?url=http%3A%2F%2Fjournals.plos.org%2Fplosmedicine%2Fs%2Ffigures&amp;data=04%7C01%7C%7C80624884120849429ea008d8efb7134e%7C1faf88fea9984c5b93c9210a11d9a5c2%7C0%7C0%7C637522916894625238%7CUnknown%7CTWFpbGZsb3d8eyJWIjoiMC4wLjAwMDAiLCJQIjoiV2luMzIiLCJBTiI6Ik1haWwiLCJXVCI6Mn0%3D%7C1000&amp;sdata=Qa%2BxG7n1XLcrlhjqaFDZ3f9oz9YAvxS4Q7g3nQ3lMY8%3D&amp;reserved=0***](https://eur01.safelinks.protection.outlook.com/?url=http%3A%2F%2Fjournals.plos.org%2Fplosmedicine%2Fs%2Ffigures&amp;data=04%7C01%7C%7C80624884120849429ea008d8efb7134e%7C1faf88fea9984c5b93c9210a11d9a5c2%7C0%7C0%7C637522916894625238%7CUnknown%7CTWFpbGZsb3d8eyJWIjoiMC4wLjAwMDAiLCJQIjoiV2luMzIiLCJBTiI6Ik1haWwiLCJXVCI6Mn0%3D%7C1000&amp;sdata=Qa%2BxG7n1XLcrlhjqaFDZ3f9oz9YAvxS4Q7g3nQ3lMY8%3D&amp;reserved=0)***. While revising your submission, please upload your figure files to the PACE digital diagnostic tool,*** [***https://eur01.safelinks.protection.outlook.com/?url=https%3A%2F%2Fpacev2.apexcovantage.com%2F&amp;data=04%7C01%7C%7C80624884120849429ea008d8efb7134e%7C1faf88fea9984c5b93c9210a11d9a5c2%7C0%7C0%7C637522916894625238%7CUnknown%7CTWFpbGZsb3d8eyJWIjoiMC4wLjAwMDAiLCJQIjoiV2luMzIiLCJBTiI6Ik1haWwiLCJXVCI6Mn0%3D%7C1000&amp;sdata=HhmlmJchnNNYDUYvLkEvVV9UXfGo7CsymNK9OMdxZ7c%3D&amp;reserved=0***](https://eur01.safelinks.protection.outlook.com/?url=https%3A%2F%2Fpacev2.apexcovantage.com%2F&amp;data=04%7C01%7C%7C80624884120849429ea008d8efb7134e%7C1faf88fea9984c5b93c9210a11d9a5c2%7C0%7C0%7C637522916894625238%7CUnknown%7CTWFpbGZsb3d8eyJWIjoiMC4wLjAwMDAiLCJQIjoiV2luMzIiLCJBTiI6Ik1haWwiLCJXVCI6Mn0%3D%7C1000&amp;sdata=HhmlmJchnNNYDUYvLkEvVV9UXfGo7CsymNK9OMdxZ7c%3D&amp;reserved=0)***. PACE helps ensure that figures meet PLOS requirements. To use PACE, you must first register as a user. Then, login and navigate to the UPLOAD tab, where you will find detailed instructions on how to use the tool. If you encounter any issues or have any questions when using PACE, please email us at*** [***PLOSMedicine@plos.org***](mailto:PLOSMedicine@plos.org)***.***

***We expect to receive your revised manuscript by Apr 15 2021 11:59PM. Please email us (***[***plosmedicine@plos.org***](mailto:plosmedicine@plos.org)***) if you have any questions or concerns.***

******Please note while forming your response, if your article is accepted, you may have the opportunity to make the peer review history publicly available. The record will include editor decision letters (with reviews) and your responses to reviewer comments. If eligible, we will contact you to opt in or out.******

***We ask every co-author listed on the manuscript to fill in a contributing author statement, making sure to declare all competing interests. If any of the co-authors have not filled in the statement, we will remind them to do so when the paper is revised. If all statements are not completed in a timely fashion this could hold up the re-review process. If new competing interests are declared later in the revision process, this may also hold up the submission. Should there be a problem getting one of your co-authors to fill in a statement we will be in contact. YOU MUST NOT ADD OR REMOVE AUTHORS UNLESS YOU HAVE ALERTED THE EDITOR HANDLING THE MANUSCRIPT TO THE CHANGE AND THEY SPECIFICALLY HAVE AGREED TO IT. You can see our competing interests policy here:*** [***https://eur01.safelinks.protection.outlook.com/?url=http%3A%2F%2Fjournals.plos.org%2Fplosmedicine%2Fs%2Fcompeting-interests&amp;data=04%7C01%7C%7C80624884120849429ea008d8efb7134e%7C1faf88fea9984c5b93c9210a11d9a5c2%7C0%7C0%7C637522916894625238%7CUnknown%7CTWFpbGZsb3d8eyJWIjoiMC4wLjAwMDAiLCJQIjoiV2luMzIiLCJBTiI6Ik1haWwiLCJXVCI6Mn0%3D%7C1000&amp;sdata=Fiv2La50C4ljdMU8KFUU9XkcyJk%2FXmjJQhawim0hErI%3D&amp;reserved=0***](https://eur01.safelinks.protection.outlook.com/?url=http%3A%2F%2Fjournals.plos.org%2Fplosmedicine%2Fs%2Fcompeting-interests&amp;data=04%7C01%7C%7C80624884120849429ea008d8efb7134e%7C1faf88fea9984c5b93c9210a11d9a5c2%7C0%7C0%7C637522916894625238%7CUnknown%7CTWFpbGZsb3d8eyJWIjoiMC4wLjAwMDAiLCJQIjoiV2luMzIiLCJBTiI6Ik1haWwiLCJXVCI6Mn0%3D%7C1000&amp;sdata=Fiv2La50C4ljdMU8KFUU9XkcyJk%2FXmjJQhawim0hErI%3D&amp;reserved=0)***.***

***Please use the following link to submit the revised manuscript:***

[***https://eur01.safelinks.protection.outlook.com/?url=https%3A%2F%2Fwww.editorialmanager.com%2Fpmedicine%2F&amp;data=04%7C01%7C%7C80624884120849429ea008d8efb7134e%7C1faf88fea9984c5b93c9210a11d9a5c2%7C0%7C0%7C637522916894625238%7CUnknown%7CTWFpbGZsb3d8eyJWIjoiMC4wLjAwMDAiLCJQIjoiV2luMzIiLCJBTiI6Ik1haWwiLCJXVCI6Mn0%3D%7C1000&amp;sdata=l7VxTY%2B3vJB5ZmpF0Ak7aQD43S%2BcFDGT01tp429pUn8%3D&amp;reserved=0***](https://eur01.safelinks.protection.outlook.com/?url=https%3A%2F%2Fwww.editorialmanager.com%2Fpmedicine%2F&amp;data=04%7C01%7C%7C80624884120849429ea008d8efb7134e%7C1faf88fea9984c5b93c9210a11d9a5c2%7C0%7C0%7C637522916894625238%7CUnknown%7CTWFpbGZsb3d8eyJWIjoiMC4wLjAwMDAiLCJQIjoiV2luMzIiLCJBTiI6Ik1haWwiLCJXVCI6Mn0%3D%7C1000&amp;sdata=l7VxTY%2B3vJB5ZmpF0Ak7aQD43S%2BcFDGT01tp429pUn8%3D&amp;reserved=0)

***Your article can be found in the "Submissions Needing Revision" folder.***

***To enhance the reproducibility of your results, we recommend that you deposit your laboratory protocols in protocols.io, where a protocol can be assigned its own identifier (DOI) such that it can be cited independently in the future. Additionally, PLOS ONE offers an option to publish peer-reviewed clinical study protocols. Read more information on sharing protocols at*** [***https://eur01.safelinks.protection.outlook.com/?url=https%3A%2F%2Fplos.org%2Fprotocols%3Futm_medium%3Deditorial-email%26utm_source%3Dauthorletters%26utm_campaign%3Dprotocols&amp;data=04%7C01%7C%7C80624884120849429ea008d8efb7134e%7C1faf88fea9984c5b93c9210a11d9a5c2%7C0%7C0%7C637522916894625238%7CUnknown%7CTWFpbGZsb3d8eyJWIjoiMC4wLjAwMDAiLCJQIjoiV2luMzIiLCJBTiI6Ik1haWwiLCJXVCI6Mn0%3D%7C1000&amp;sdata=h6JPNseS%2BiSVX431%2FDKYioo49D7B0ml9vxkumb4nssE%3D&amp;reserved=0***](https://eur01.safelinks.protection.outlook.com/?url=https%3A%2F%2Fplos.org%2Fprotocols%3Futm_medium%3Deditorial-email%26utm_source%3Dauthorletters%26utm_campaign%3Dprotocols&amp;data=04%7C01%7C%7C80624884120849429ea008d8efb7134e%7C1faf88fea9984c5b93c9210a11d9a5c2%7C0%7C0%7C637522916894625238%7CUnknown%7CTWFpbGZsb3d8eyJWIjoiMC4wLjAwMDAiLCJQIjoiV2luMzIiLCJBTiI6Ik1haWwiLCJXVCI6Mn0%3D%7C1000&amp;sdata=h6JPNseS%2BiSVX431%2FDKYioo49D7B0ml9vxkumb4nssE%3D&amp;reserved=0)

***Please ensure that the paper adheres to the PLOS Data Availability Policy (see*** [***https://eur01.safelinks.protection.outlook.com/?url=http%3A%2F%2Fjournals.plos.org%2Fplosmedicine%2Fs%2Fdata-availability&amp;data=04%7C01%7C%7C80624884120849429ea008d8efb7134e%7C1faf88fea9984c5b93c9210a11d9a5c2%7C0%7C0%7C637522916894625238%7CUnknown%7CTWFpbGZsb3d8eyJWIjoiMC4wLjAwMDAiLCJQIjoiV2luMzIiLCJBTiI6Ik1haWwiLCJXVCI6Mn0%3D%7C1000&amp;sdata=98g7oT8MpMjkCB%2BGM9WC5ZmE%2FtzdHR6uyhq4csaCVyk%3D&amp;reserved=0***](https://eur01.safelinks.protection.outlook.com/?url=http%3A%2F%2Fjournals.plos.org%2Fplosmedicine%2Fs%2Fdata-availability&amp;data=04%7C01%7C%7C80624884120849429ea008d8efb7134e%7C1faf88fea9984c5b93c9210a11d9a5c2%7C0%7C0%7C637522916894625238%7CUnknown%7CTWFpbGZsb3d8eyJWIjoiMC4wLjAwMDAiLCJQIjoiV2luMzIiLCJBTiI6Ik1haWwiLCJXVCI6Mn0%3D%7C1000&amp;sdata=98g7oT8MpMjkCB%2BGM9WC5ZmE%2FtzdHR6uyhq4csaCVyk%3D&amp;reserved=0)***), which requires that all data underlying the study's findings be provided in a repository or as Supporting Information. For data residing with a third party, authors are required to provide instructions with contact information for obtaining the data. PLOS journals do not allow statements supported by "data not shown" or "unpublished results." For such statements, authors must provide supporting data or cite public sources that include it.***

***We look forward to receiving your revised manuscript.***

***Sincerely,***

***Beryne Odeny,***

***PLOS Medicine***

***plosmedicine.org***

***-----------------------------------------------------------***

***Requests from the editors:***

***-	Please revise your title according to PLOS Medicine's style. Your title must be nondeclarative and not a question. It should begin with main concept if possible. Please place the study design ("A retrospective cohort study,") in the subtitle (i.e., after a colon).***

We have changed the title from “Ethnic differences in guideline-indicated statin initiation for people with type 2 diabetes: a longitudinal cohort study using the Clinical Practice Research Datalink, 2006-2019” to “Ethnic differences in guideline-indicated statin initiation for people with type 2 diabetes: a **retrospective** cohort study using the Clinical Practice Research Datalink, 2006-2019”.

***-	Abstract summary - At this stage, we ask that you reformat your non-technical Author Summary. The Author Summary should immediately follow the Abstract in your revised manuscript. This text is subject to editorial change and should be distinct from the scientific abstract. The summary should be accessible to a wide audience that includes both scientists and non-scientists. Please see our author guidelines for more information:*** [***https://eur01.safelinks.protection.outlook.com/?url=https%3A%2F%2Fjournals.plos.org%2Fplosmedicine%2Fs%2Frevising-your-manuscript%23loc-author-summary&amp;data=04%7C01%7C%7C80624884120849429ea008d8efb7134e%7C1faf88fea9984c5b93c9210a11d9a5c2%7C0%7C0%7C637522916894625238%7CUnknown%7CTWFpbGZsb3d8eyJWIjoiMC4wLjAwMDAiLCJQIjoiV2luMzIiLCJBTiI6Ik1haWwiLCJXVCI6Mn0%3D%7C1000&amp;sdata=mjbAYjGoCPkhTDbX73u8Is6KwjmKR0jzWhWmH5Lu1Ek%3D&amp;reserved=0***](https://eur01.safelinks.protection.outlook.com/?url=https%3A%2F%2Fjournals.plos.org%2Fplosmedicine%2Fs%2Frevising-your-manuscript%23loc-author-summary&amp;data=04%7C01%7C%7C80624884120849429ea008d8efb7134e%7C1faf88fea9984c5b93c9210a11d9a5c2%7C0%7C0%7C637522916894625238%7CUnknown%7CTWFpbGZsb3d8eyJWIjoiMC4wLjAwMDAiLCJQIjoiV2luMzIiLCJBTiI6Ik1haWwiLCJXVCI6Mn0%3D%7C1000&amp;sdata=mjbAYjGoCPkhTDbX73u8Is6KwjmKR0jzWhWmH5Lu1Ek%3D&amp;reserved=0)***.***

We have added the following after the abstract:

**“Author summary**

**Why was this study done?**

-	People of South Asian and African/ African Caribbean ethnicity living in the UK are more likely to have type 2 diabetes than people of European ethnicity, and have higher rates of the cardiovascular complications of diabetes, e.g. heart attacks and strokes.

-	Lowering blood cholesterol with statin treatment reduces cardiovascular complications for people with type 2 diabetes.

-	Previous studies have suggested there may be ethnic differences in statin use for people with diabetes, however, no study has sought potential explanations for ethnic differences in statin prescribing or accounted for contemporary prescribing guidelines.

-	Therefore, we aimed to provide a timely, nationally representative assessment of ethnic differences in guideline-indicated statin prescribing in type 2 diabetes, and explanations for them.

**What did the researchers do and find?**

-	Using routinely recorded primary care records from 834 UK general practices, we identified people with newly diagnosed type 2 diabetes who were eligible for statin treatment for primary prevention of cardiovascular disease. We then compared rates of statin initiation between the UK’s three largest ethnic groups; people of European, South Asian and African/ African Caribbean ethnicity.

-	We found that people of African/ African Caribbean ethnicity with type 2 diabetes were 24% less likely to receive guideline-indicated statin treatment than people of European ethnicity, and people of South Asian ethnicity were 9% less likely.

-	Ethnic differences remained after allowing for differences in cholesterol levels, other cardiovascular risk factors, demographic factors, deprivation, healthcare usage, comorbidity and polypharmacy.

-	We estimated that equalising statin prescribing rates across these three ethnic groups would prevent up to 12,600 atherosclerotic cardiovascular disease events (such as heart attacks and strokes) in people with type 2 diabetes.

**What do these findings mean?**

-	People with type 2 diabetes of African/ African Caribbean and South Asian ethnicities are less likely to be prescribed a statin for primary prevention of cardiovascular disease in the UK than people of European ethnicity.

-	Targeted policies to increase the use of statins among people of African/ African Caribbean or South Asian ethnicity with type 2 diabetes could substantially reduce the excess burden of future cardiovascular events in these groups.”

***-	Abstract:***

***1.	Please structure your abstract using the PLOS Medicine headings (Background, Methods and Findings, Conclusions).***

***2.	Please combine the Methods and Findings sections into one section, “Methods and findings”. Please ensure that all numbers presented in the abstract are present and identical to numbers presented in the main manuscript text.***

***3.	Please include the actual amounts or percentages of relevant outcomes, not just hazard ratios or relative risks.***

***4.	Please quantify the main results (with 95% CIs and p values).***

***5.	Please include a summary of adverse events if these were assessed in the study.***

***6.	In the last sentence of the Abstract Methods and Findings section, please describe the main limitation(s) of the study's methodology.***

***7.	Abstract Conclusions: Please address the study implications without overreaching what can be concluded from the data; the phrase "In this study, we observed ..." may be useful. Please interpret the study based on the results presented in the abstract, emphasizing what is new.***

Thanks for these comments, we have addressed them all, except for number 5. (since adverse events were not part of the study question). The new version of the abstract is below:

**“Abstract**

**Background:** Type 2 diabetes is 2-3 times more prevalent in people of South Asian and African/ African Caribbean ethnicity than people of European ethnicity living in the UK. The former two groups also experience excess atherosclerotic cardiovascular disease (ASCVD) complications of diabetes. We aimed to study ethnic differences in statin initiation, a cornerstone of ASCVD primary prevention, for people with type 2 diabetes.

**Methods and findings:** Observational cohort study of UK primary care records, from 1^st^ January 2006 to 30^th^ June 2019. Data were studied from 27,511 (88%) people of European ethnicity, 2,386 (8%) people of South Asian ethnicity and 1,142 (4%) people of African/ African Caribbean ethnicity with: incident type 2 diabetes, no previous atherosclerotic cardiovascular disease (ASCVD) and statin use indicated by guidelines. Statin initiation rates were contrasted by ethnicity and the number of ASCVD events prevented by equalising prescribing rates across ethnic groups was also estimated. Median times to statin initiation were 79, 109 and 84 days for people of European, South Asian and African/ African Caribbean ethnicity respectively. People of African/ African Caribbean ethnicity were a third less likely to receive guideline-indicated statins than European people (n/N [%]: 605/1,142 [53%] and 18,803/27,511 [68%] respectively, age- and gender-adjusted HR 0.67 [95% CI 0.60 to 0.76], p<0.001). HRs attenuated marginally in models adjusting for TC/HDL (0.77 [0.69 to 0.85], p<0.001), with no further diminution when deprivation, ASCVD risk factors, comorbidity, polypharmacy and healthcare usage were accounted for (fully adjusted HR 0.76 [0.68, 0.85], p<0.001). People of South Asian ethnicity were 9% less likely to receive a statin than European groups (1,489/2,386 [62%] and 18,803/27,511 [68%] respectively, fully adjusted HR 0.91 [0.85 to 0.98], p<0.001, adjusting for all covariates). We estimated that up to 12,600 ASCVD events could be prevented in people with diabetes by equalising statin prescribing across ethnic groups. Limitations included completeness of recording of routinely collected data.

**Conclusions:** In this study we observed that people of African/ African Caribbean ethnicity with type 2 diabetes were substantially less likely and people of South Asian ethnicity marginally less likely to receive guideline-indicated statins than people of European ethnicity, even after accounting for socio-demographics, healthcare usage, ASCVD risk factors or co-morbidity. Under-use of statins in people of African/ African Caribbean or South Asian ethnicity with type 2 diabetes is a missed opportunity to prevent cardiovascular events.”

***-	Please conclude the Introduction with a clear description of the study question or hypothesis.***

We have replaced the final paragraph of the introduction with a more detailed version, as below (line 132 of marked-up copy, line 119 of clean copy):

“Therefore, using a nationally representative database of prescribing records, we aimed to study ethnic differences in statin initiation for the statin eligible (according to guidelines^10^) population with type 2 diabetes. Specifically, we: i) contrasted statin initiation rates for people of European, South Asian and African/ African Caribbean ethnicity, ii) sought explanations for differences found and iii) estimated the population impact of equalising statin prescribing for the UK’s three main ethnic groups.”

***-	For this observational study, in the manuscript text, please indicate: (1) the analytical methods by which you planned to test your hypothesis, (2) the analyses you actually performed, and (3) when reported analyses differ from those that were planned, transparent explanations for differences that affect the reliability of the study's results. If a reported analysis was performed based on an interesting but unanticipated pattern in the data, please be clear that the analysis was data-driven.***

The analytical methods are to be found in the “statistical analysis” section of the methods and were identical to those pre-specified. We have now clarified this (see excerpt below).

***-	Did your study have a prospective protocol or analysis plan? Please state this (either way) early in the Methods section.***

a) ***If a prospective analysis plan (from your funding proposal, IRB or other ethics committee submission, study protocol, or other planning document written before analyzing the data) was used in designing the study, please include the relevant prospectively written document with your revised manuscript as a Supporting Information file to be published alongside your study, and cite it in the Methods section. A legend for this file should be included at the end of your manuscript.***
b) ***If no such document exists, please make sure that the Methods section transparently describes when analyses were planned, and when/why any data-driven changes to analyses took place.***
c) ***In either case, changes in the analysis-- including those made in response to peer review comments-- should be identified as such in the Methods section of the paper, with rationale.***

The planned analyses were identical to those performed; we have added a sentence on this at the start of the “statistical analysis” section of the methods, which reads as follows (line 244 of marked-up copy, line 223 of clean copy):

“All analytic methods were pre-specified in a scientific protocol (see protocol of S1 Protocol).”

The protocol for ethics approval is now added to the supporting information, and a legend for it inserted at the end of the manuscript.

***-	Please ensure that the study is reported according to the STROBE guideline, and include the completed STROBE checklist as Supporting Information. Please add the following statement, or similar, to the Methods: "This study is reported as per the Strengthening the Reporting of Observational Studies in Epidemiology (STROBE) guideline (S1 Checklist)." The STROBE guideline can be found here:*** [***https://eur01.safelinks.protection.outlook.com/?url=http%3A%2F%2Fwww.equator-network.org%2Freporting-guidelines%2Fstrobe%2F&amp;data=04%7C01%7C%7C80624884120849429ea008d8efb7134e%7C1faf88fea9984c5b93c9210a11d9a5c2%7C0%7C0%7C637522916894625238%7CUnknown%7CTWFpbGZsb3d8eyJWIjoiMC4wLjAwMDAiLCJQIjoiV2luMzIiLCJBTiI6Ik1haWwiLCJXVCI6Mn0%3D%7C1000&amp;sdata=7%2BxSRU8oIZjNaf5%2BrXSE1mTINBsGVCOb0aKbiv36Yk4%3D&amp;reserved=0***](https://eur01.safelinks.protection.outlook.com/?url=http%3A%2F%2Fwww.equator-network.org%2Freporting-guidelines%2Fstrobe%2F&amp;data=04%7C01%7C%7C80624884120849429ea008d8efb7134e%7C1faf88fea9984c5b93c9210a11d9a5c2%7C0%7C0%7C637522916894625238%7CUnknown%7CTWFpbGZsb3d8eyJWIjoiMC4wLjAwMDAiLCJQIjoiV2luMzIiLCJBTiI6Ik1haWwiLCJXVCI6Mn0%3D%7C1000&amp;sdata=7%2BxSRU8oIZjNaf5%2BrXSE1mTINBsGVCOb0aKbiv36Yk4%3D&amp;reserved=0)

Thanks, we have now added the completed STROBE checklist to the supporting information.

***-	Your study is observational and therefore causality cannot be inferred. Please remove language that implies causality, such as - predict, risk, effect, etc. Instead, refer to associations consistently throughout the text.***

Thanks, we have done this.

***-	How was race/ethnicity defined and by whom? Why was race/ethnicity considered important in this study and what it is believed to represent e.g., are SES or genetic differences being attributed to race/ethnicity?***

Ethnicity was defined by the patients themselves, as previously stated in the first sentence of the “covariates” section of the methods: “The main exposure in this study, **self-reported** European, South Asian or African/ African Caribbean ethnicity, was designated using ethnicity Read codes, usually recorded by primary care staff during a patient’s initial registration with a practice.” We have now changed this sentence, for clarification, to (line 191 of marked-up copy, line 179 of clean copy): “The main exposure in this study was **self-reported (i.e. assigned by the patient themselves)** European, South Asian or African/ African Caribbean ethnicity.”

We used a modified version of a previously published code list (Mathur R et al 2014) to search for ethnicity codes within the CPRD database and (as stated in the paper) “A previously published algorithm^16^ adjudicated on cases of multiple or differing codes per patient.”. The ethnicity code list was given in the appendix of the initial submission for reproducibility purposes.

The paper is concerned with ethnicity rather than race – the latter uses primarily physical traits to group people. We have added to the following sentence to the manuscript to highlight this (line 192 of marked-up copy, line 180 of clean copy): “Ethnicity is a complex construct that groups people together who identify with each other through a shared culture, and encompasses factors in common such as cultural heritage, ancestry, language, history, diet or religion^15, 16^. Thus, it is most appropriately self-defined, according to the group the individual most identifies with^17^.”

Therefore, in answer to the reviewer’s question about what ethnicity represents in this study, the answer is socio-cultural factors, rather than genetic/ biological ones. As far as we know, there is no biological basis governing UK statin prescribing by ethnicity for the three ethnic groups in question. Therefore, the proximal factors that are represented by ethnicity in this study are likely to be socio-cultural ones; we presume either prescribing behaviour (at the level of the physician) or health-seeking behaviour (at the level of the patient). Unfortunately, this study has no way of unpicking those two potential explanations, as already outlined in the discussion (line 490 of marked-up copy, line 442 of clean copy):

“We could not reliably distinguish between people being offered a statin and declining, versus not being offered a statin per se, and reasons for statin declinature were infrequently recorded, especially for African/ African Caribbean people (4%), so it is impossible to pinpoint exact reasons for under-prescribing. A portion of it may be due to patient choice, which may not be adequately informed^36^. If under-prescribing is at the GP’s discretion, this may be due to inadequate knowledge of ethnic differences in ASCVD risk (e.g. that whilst African/ African Caribbean groups have lower risks of coronary disease, their risk of stroke far surpasses that of Europeans, regardless of cholesterol levels^3^); an improvement in this over time may explain the smaller ethnic differences in the latter years of the study. The broader concept of structural racism, encompassing all of the factors above, is an increasingly recognised source of health inequalities ^37^.”

***-	In statistical methods, please refer to any post-hoc corrections to correct for multiple comparisons during your statistical analyses. If these were not performed please justify the reasons. Please refer to our statistical reporting guidelines for assistance (***[***https://eur01.safelinks.protection.outlook.com/?url=https%3A%2F%2Fjournals.plos.org%2Fplosone%2Fs%2Fsubmission-guidelines.%23loc-statistical-reporting&amp;data=04%7C01%7C%7C80624884120849429ea008d8efb7134e%7C1faf88fea9984c5b93c9210a11d9a5c2%7C0%7C0%7C637522916894625238%7CUnknown%7CTWFpbGZsb3d8eyJWIjoiMC4wLjAwMDAiLCJQIjoiV2luMzIiLCJBTiI6Ik1haWwiLCJXVCI6Mn0%3D%7C1000&amp;sdata=zpCyoy3dXftgBXhWFMvY4F8C%2B0oOaIfnsnjgAaHjiNw%3D&amp;reserved=0***](https://eur01.safelinks.protection.outlook.com/?url=https%3A%2F%2Fjournals.plos.org%2Fplosone%2Fs%2Fsubmission-guidelines.%23loc-statistical-reporting&amp;data=04%7C01%7C%7C80624884120849429ea008d8efb7134e%7C1faf88fea9984c5b93c9210a11d9a5c2%7C0%7C0%7C637522916894625238%7CUnknown%7CTWFpbGZsb3d8eyJWIjoiMC4wLjAwMDAiLCJQIjoiV2luMzIiLCJBTiI6Ik1haWwiLCJXVCI6Mn0%3D%7C1000&amp;sdata=zpCyoy3dXftgBXhWFMvY4F8C%2B0oOaIfnsnjgAaHjiNw%3D&amp;reserved=0)***)***

Thank you. No post-hoc corrections for multiple comparisons were made, as the pre-specified primary analysis consisted of a single three group comparison. We presented nested models, constructed by adjusting an age and gender-adjusted model for each factor in the fully-adjusted model in turn to see which attenuated the estimates the most (See figure 1), but these do not constitute separate comparisons. We have now emphasised this in the paper (line 263 of marked-up copy, line 241 of clean copy):

“**The pre-specified primary comparison** of statin initiation for people of South Asian or African/ African Caribbean vs. European ethnicity was studied using multi-level Cox proportional hazards models with robust standard errors to account for within practice clustering of prescribing patterns.”

However, we do acknowledge that we have some sub-group and sensitivity analyses which may constitute multiple comparisons. We did not make statistical corrections for multiple testing for these, as such corrections generally rely on the manipulation of p-values to interpret results (Perneger T 1998), but our interpretation of results has not been governed by p-values (see below). Further, they are likely to be over-conservative for non-independent comparisons. In lieu of this, we have firstly edited the text to highlight that these were secondary analyses, and that they were pre-specified, e.g. in the statistical analysis section of the methods:

“**Pre-specified secondary** sub-group analyses were performed by inspecting…” (line 286 of marked-up copy, line 262 of clean copy)

“**Pre-specified secondary** sensitivity analyses explored…” (line 293 of marked-up copy, line 269 of clean copy)

In the results section:

“**Secondary** sub-group analyses showed that ethnic differences in statin initiation appeared to be…” (line 358 of marked-up copy, line 335 of clean copy)

“Results of further **secondary** sub-group … and sensitivity analyses..” (line 388 of marked-up copy, line 364 of clean copy)

Secondly, we have de-emphasised these secondary analyses throughout the paper (and they have now been removed from the abstract and the initial summary paragraph of the discussion), and thirdly, they are interpreted cautiously, e.g.:

“…..these findings should be interpreted with caution as confidence intervals were very wide due to low power, and do in fact include the larger differences present for the prior periods.” (line 370 of marked-up copy, line 346 of clean copy)

“Nevertheless, the distinctly lower statin initiation rates we show for the Bangladeshi population in our secondary analysis of ethnic sub-groups are discordant and should be interpreted with caution..” (line 469 of marked-up copy, line 423 of clean copy)

“Also in keeping with the results **suggested** by our secondary analyses…” (line 473 of marked-up copy, line 425 of clean copy)

***-	Please describe how you selected your adjustment variables.***

We have added the following sentence to the second paragraph of the “covariates” section of the methods (line 205 of marked-up copy, line 192 of clean copy):

“Covariates were selected pragmatically (i.e. governed by what was available in the dataset) to enable investigation of the following areas of potential confounding: sociodemographics (age, gender, socio-economic deprivation), ASCVD risk factors (smoking TC/HDL, BMI, antihypertensive use), healthcare usage (consultation rate), comorbidity (four chronic disease areas) and polypharmacy (number of medications), and to enable investigation of effect modification by the following: degree of hyperlipidaemia, geographical location (London vs. non-London practice and country within the UK – examined in separate sets of models), guideline time period, ethnic sub-group and gender.”

***-	We note the potential for unobservable confounding in this observational study. Please consider using robust methods such as propensity score matching to address this.***

Thanks for this, and yes, we agree residual confounding is likely, as in any observational study. We have acknowledged this in the fourth paragraph of the discussion already (line 487 of marked-up copy, line 439 of clean copy):

“Further explanatory factors, such as deprivation, may have been obscured by residual confounding…”

However, we did not wish to use a propensity score here to “equalise” the ethnic groups in terms of confounders because one of our aims was to identify explanations for the ethnic differences in prescribing. To do this we needed the freedom to be able to adjust for each potential confounder/ set of confounders separately to seek factors that may have attenuated the ethnicity-prescribing associations, rather than applying all potential confounders at once to the models. Further, propensity scores do not solve the problem of unmeasured confounding to a greater extent than standard adjustment (Kim 2016).

***-	Please specify the significance level used (eg, P<0.05, two-sided) and the statistical test used to derive a p value.***

We did not use p-values in our interpretation of the results as we were primarily interested in whether the observed observations were likely to be clinically (rather than statistically) significant at the population level, so we relied on inspection of the magnitude of the associations, coupled with inspection of 95% confidence intervals to determine whether the likely upper boundary of the true association excluded the null value (i.e. a hazard ratio of 1) (Wasserstein et al 2016, Wasserstein et al 2019). We have added the following sentence to the penultimate paragraph of the “statistical analysis” section of the methods (line 280 of marked-up copy, line 256 of clean copy):

“We evaluated the likelihood of the results indicating a true ethnic difference chiefly via inspection of the 95% confidence intervals of a given association^26^, generated using standard errors from the two-sided z test from Cox regression models.”

***-	Please include line numbers in your next draft.***

We have now done this.

***-	Please delete the word “virtually” from the 3rd paragraph of the Results section.***

Thanks, this has been done.

***************************************************************************

***Comments from the reviewers:***

***Reviewer #1: I confine my remarks to statistical aspects of this paper. While the general statistical approach is fine, I do have some issues to resolve before I can recommend publication.***

***General questions:***

***Did you distinguish between people who were offered statins and refused them vs. those who never were offered them?***

We did not identify people who were/ were not offered statins per se, as rates of recording this were extremely low and likely to be of limited usefulness in the analysis. However, we noted whether Read codes for statin declinature were present (see table 2, third line from the bottom). But, as with the codes for having had a statin offered, it is impossible to know whether the lack of codes for declinature were because people didn’t decline or because declinature was not recorded. Therefore, we cannot reliably disentangle “not offered” from “declined”. We have added a sentence to the discussion to this effect (line 490 of marked-up copy, line 442 of clean copy):

“We could not reliably distinguish between people being offered a statin and declining, versus not being offered a statin per se, and reasons for statin declinature were infrequently recorded, especially for African/ African Caribbean people (4%), so it is impossible to pinpoint exact reasons for under-prescribing.”

***Has anyone looked at any ethnic based differences in the effects of statins?***

Yes, in the UK a sub-study of the Anglo-Scandinavian Cardiac Outcomes Trial (ASCOT) trial examined ethnic differences in lipid-lowering medication; no ethnic differences were found in statin response between people of South Asian or African/ African Caribbean, compared to those of European ethnicity (Chapman et al 2011). We have added this to the introduction as follows (line 125 of marked-up copy, line 113 of clean copy):

“Evidence suggests statins are equally effective in people of European, South Asian and African/ African Caribbean ethnicity^15^, so there is no rationale for restricting their use on the basis of ethnicity.”

***Was collinearity investigated? It seems likely to be present, with this set of covariates.***

Thanks for this, when collinearity diagnostics were performed on the model adjusted for all the covariates (i.e. ethnicity, age, gender, index of multiple deprivation, BMI, TC/HDL ratio, quartiles of medication use in preceding year, prevalent cancer, prevalent asthma/ COPD, prevalent CKD, prevalent serious mental illness, quartiles of number of consultations in preceding year and prevalent antihypertensive use; as displayed in figure 1), there were high variance inflation factors (VIFs) for age (15.47), gender (10.27), BMI (17.93) and TC/ HDL ratio (10.04), with the mean VIF for the model=3.98.

We considered age and gender to be a priori variables, given their ethnic differences at baseline and influence on statin prescribing, but we removed BMI and TC/HDL from the model to assess the impact of reducing collinearity. We also contrasted the model with BMI and TC/ HDL removed to one with just age, gender and TC/HDL, see below:

| **Model number**  ***and factors*** | **South Asian vs European ethnicity** | | | | **African/ African Caribbean vs European ethnicity** | | | |
| --- | --- | --- | --- | --- | --- | --- | --- | --- |
|  | **HR** | **Robust SE** | **95% CI** | **p** | **HR** | **Robust SE** | **95% CI** | **p** |
| **1**  *Age, gender, index of multiple deprivation, BMI, TC/HDL ratio, quartiles of medication use in preceding year, prevalent cancer, prevalent asthma/ COPD, prevalent CKD, prevalent serious mental illness, quartiles of number of consultations in preceding year and prevalent antihypertensive use* | 0.91 | 0.031 | 0.85, 0.97 | 0.008 | 0.76 | 0.043 | 0.68, 0.85 | <0.001 |
| **2**  *As above, excepting BMI and TC/ HDL ratio* | 0.88 | 0.029 | 0.83, 0.94 | <0.001 | 0.68 | 0.041 | 0.61, 0.77 | <0.001 |
| **3**  *Age, gender, TC/ HDL* | 0.93 | 0.030 | 0.87, 0.99 | 0.01 | 0.77 | 0.042 | 0.69, 0.85 | <0.001 |

When BMI and TC/ HDL are removed from the model (model 2 vs. model 1), the deflation in standard errors is minimal and the increase in precision does not affect the interpretation of the findings. Furthermore, both the standard errors and the HRs in model 3 are comparable with those in model 2. Therefore, we do not believe that collinearity has affected the interpretation of the findings. We have added a sentence in the “statistical analysis” sections of the methods (line 282 of marked-up copy, line 258 of clean copy):

“ Collinearity was investigated between variables for the model containing all covariates by calculating variance inflation factors (VIFs), and variables with VIFs ≥10 were excluded in sensitivity analyses to assess the impact on standard errors.”

We also now report these findings in the results section (line 400 of marked-up copy, line 375 of clean copy):

“Collinearity diagnostics of the main model featuring all covariates (i.e. the last model shown in figure 1) revealed VIFs≥10 for BMI and TC/ HDL, as well as the forced variables of age and gender. When BMI and TC/ HDL were removed from the model, the resulting deflation in standard errors was minimal and did not affect the interpretation of the results, therefore we deemed the collinearity of the model within acceptable parameters for the purposes of results interpretation.”

***p 4 - near top "leading cause of death" depends entirely on how causes are listed. Is cancer one cause or many? Is a heart attack different from a stroke? Instead, give numbers of deaths or something like that.***

Thanks for this, we have replaced this sentence with the following (line 93 of marked-up copy, line 83 of clean copy):

“Type 2 diabetes is a potent risk factor for cardiovascular disease, itself causing 17.9 million deaths worldwide per year, 80% of which are due to myocardial infarction and stroke combined^1^.”

***p. 6 - the location variable should be ONE variable with levels something like a) London b) Other England c) Other UK, The way you have it, no one can be both "London" and "other UK"***

Location was not included in the main analysis (figure 1), but instead included in 2 separate sub-group analyses, the first by London/ non-London practice (figure 2, section b)), the second by country within the UK (supplementary figure 1 b)). At no point were the 2 location variables used in a model together. We have now clarified this in the second paragraph of the “covariates” section of the methods (line 211 of marked-up copy, line 197 of clean copy):

“Covariates were selected pragmatically ……. to enable investigation of effect modification by the following…. geographical location (London vs. non-London practice and country within the UK – **examined in separate sets of models**)…””

***The e tables were rather oddly formatted. This is more a matter of style but I found the format a bit confusing. The cases data should be in the left most column. In table e2 what is "p for"? There are some good guidelines for tables in the American Psychological Association Style Guide (but, if these tables are OK with PLOS then I am not going to prevent publication for that reason alone).***

***Figure 3 was a 3D bar chart. These aren't good (see the work of William S. Cleveland). You could use a dot plot, or maybe a mosaic plot, depending on what you want to highlight.***

***Peter Flom***

The supplement does not contain a table labelled e2. Our supplementary Table 2 does not contain “p for” or indeed any p-values, and table formatting is similar to the main paper. Also, there are no bar charts in the manuscript, and no figure 3. We wonder if it is possible that these comments and the one above were made in error, and/or the wrong supplement/display item files were assessed? We are of course happy to address any comments on our display items once this has been clarified.

***************************************************************************

***Reviewer #2: The article by Eastwood et al addresses a very relevant question regarding ethnic inequalities in health care delivery. To address this issue, the authors used the well-known real-world practice database CPRD from the UK. The final conclusion is that there is considerable underuse of statin treatment for primary cardiovascular disease prevention in people with type 2 diabetes from non-European ethnicity. The authors used sound methodology. Although the study has some limitations, including its retrospective design and other limitations associated with the design, the final message indicates that we should raise awareness among physicians and also among health care authorities. This reviewer, a non-expert in statistical analysis, did not find any methodological issues affecting the validity of the data. However, the following comments/changes should be addressed:***

***- The authors should include other non-UK studies both in the introduction and the discussion of the paper. The audience of this journal is an international one, and, as such, an international perspective should be adopted.***

Thanks very much for this point, and apologies for the omission. We have removed this sentence (which only applies to UK data) from the introduction:

“Further, at the practice level, there is evidence that fewer statins are prescribed in areas with proportionally more patients from non-White ethnic groups, even after accounting for differences in age and comorbidity.”

and have now added the following sentence to the introduction (line 120 of marked-up copy, line 108 of clean copy):

“Furthermore, several US studies of health records data indicate substantial under-prescribing of statins to African American people with diabetes in whom statins are indicated, in comparison to their white counterparts ^11-14^; though we are unaware of any similar North American data for people of South Asian ethnicity.”

Additionally, we have now modified the following sentence in the discussion (line 457 of marked-up copy, line 411 of clean copy):

“Our finding of marked statin under-prescribing for primary prevention in people with type 2 diabetes of African/ African Caribbean ethnicity is commensurate with previous work from the UK^5, 6^ **and the USA^11-14^**.”

Finally, an important point, when considering health inequity in both the USA and the UK is the differences in healthcare provision, and as such, we have added to the discussion section the following sentence (line 458 of marked-up copy, line 412 of clean copy):

“Arguably, inequalities in prescribing may be anticipated in the US fee-for-service health care system, where inter-ethnic socioeconomic disparities influence health insurance coverage, and thus may place non-white ethnic groups at a disadvantage^12^. However, it is striking that we see similar levels of inequality in the UK, where health care is free at the point of delivery.”

***- Please, give an explanation on the reason for only including subjects without cardiovascular disease. Probably, the inclusion of subjects in secondary prevention may have yielded relevant information on ethnicity issues.***

Thanks for this point, and yes, we agree a comparison of statin prescribing for secondary prevention is an important line of enquiry. However, populations receiving statins for primary and secondary prevention are not readily comparable in our dataset, since prescribing for secondary prevention is often initiated in secondary care. Dates of both the triggering event and the initial statin prescription are generally either not present in primary care records, or unlikely to be accurate. Thus, an examination of secondary prevention prescribing would necessitate a different modelling strategy and the implications of the results would also differ substantially. Therefore, we deemed it outside the scope of this manuscript. We have added the following sentence to the outcome section of the methods (line 184 of marked-up copy, line 172 of clean copy):

“We did not examine ethnic differences in statin prescribing for secondary prevention. This is an important, but different question and would have required a different approach, as initiation of statins for secondary prevention most commonly occurs in secondary care settings, making it difficult to establish index dates of statin eligibility and initial prescription.”

***- Under Method, there is no clear explanation for using as exclusion criteria those people with missing data for TC/HDL, BMI and smoking, as explained under Results. Why did they choose these variables?***

Thanks for this point, we agree the rationale for this was not clear in the original version of the paper, and have added to the following sentence to the methods section to remedy this (line 250 of marked-up copy, line 229 of clean copy):

“Of note, most covariates of interest were complete in our data: ethnicity was required for inclusion in the study population, age, sex and deprivation had no missing data, and other variables were defined by presence/ absence of specific codes in the health record (e.g. specific diagnoses or medications, healthcare usage), so could not be missing. The only variables with missing data were therefore smoking status, TC/ HDL and BMI, which relied solely on physicians’ recording, and thus were not complete for all individuals.”

***************************************************************************

***Reviewer #3:***

***This is a well researched and well written paper with some major and minor issues. I think the handling of missing values and the inclusion of three NICE time periods in one study are the major issues that need to be better justified.***

***1. 1st bullet point in the "What this study adds" section and Conclusions in the Abstract has 25%. It is not clear where this number came from.***

Thanks. It comes from the HR of statin initiation for people of African/ African Caribbean ethnicity vs those of European ethnicity in the final model with all the covariates included (0.76 [0.68,0.85], p<0.001), see the bottom line of figure 1. Strictly speaking, we found a 24% reduction. What may have been confusing is the we reported only the HR for the TC/ HDL adjusted model in the abstract, therefore we have added to HR for the full model to the results section of the abstract – see below in bold (line 39 of marked-up copy, line 28 of clean copy):

“HRs attenuated marginally in models adjusting for TC/HDL (0.77 [0.69 to 0.85], p<0.001), with no further diminution when deprivation, ASCVD risk factors, comorbidity, polypharmacy and healthcare usage were accounted for **(0.76 [0.68, 0.85], p<0.001)**.”

As stated, more accurately the reduction was 24% - we have now changed to 25% to 24% throughout the paper. We have also changed the 10% quoted for the South Asian vs European comparison to the more accurate 9%. Apologies, the rounding up was purely to increase the simplicity of the message to the casual reader.

***2. 3rd bullet point in the "What this study adds" section reads more like a conjecture, not what this study adds to the body of science. Please revise.***

Thanks for this. Given that we have now included a statement on the impact assessment in the “What did the researchers do and find?” section of this author summary (see your point 10 below), we have retained the point regarding improved targeting, albeit reworded, as it follows on as a potential policy implication from the point on the impact assessment in the “What did the researchers do and find?” section of the author summary (line 89 of marked-up copy, line 78 of clean copy):

“Targeted policies to increase the use of statins among people of African/ African Caribbean or South Asian ethnicity with type 2 diabetes could substantially reduce the excess burden of future cardiovascular events in these groups.”

We can of course remove it if the reviewer still finds it objectionable.

***3. Page 4, Introduction - "governed by the NICE guidelines". This is ambiguous. Is it recommended for all patients with T2D? In what strength? For example, American Diabetes Association recommends moderate intensity statins to all persons with diabetes over age 40 years.***

Thanks for this useful point. We have now clarified (in brief) the NICE guidelines in the introduction with the following sentence (line 105 of marked-up copy, line 95 of clean copy):

“NICE guidelines have varied by time period regarding thresholds for statin prescription; the 2006 guideline designated type 2 diabetes a secondary prevention state and thus advocated universal prescription^9^, the 2008 guideline recommended statin use in all ≥40 years old or with a 10 year ASCVD risk of ≥20% or with ≥1 other ASCVD risk factor^8^, whilst the 2014 guideline advised high-intensity statin prescription (e.g. atorvastatin 20mg) simply for all with a 10 year ASCVD risk of ≥10%^10^ (diabetes at this point being a multiplier in the recommended ASCVD risk equation).”

***4. Page 5, first paragraph. How were the guidelines different between versions in terms of statin recommendations. Table 1 has this information. A brief summary of the changes in statin recommendations in the introduction will be very helpful. I think this is critically important for your readers to understand the baseline for the time to statin initiation you used in the study. I am also curious about whether combining the three periods in one analysis is valid. Because the recommendations changed over time, for example, all people aged 40 y or older or ASCVD 10-year risk >= 10% in NICE 2008, how was your inclusion criteria differed from NICE 2006 period or NICE 2014 period? There should be more justification about combining the three periods. Sensitivity analyses on this issue would be very helpful.***

See above regarding your first point on the differences between the different versions of the guidelines.

Re. justification, we have added the following sentence to the “study population” part of the methods section on this (line 166 of marked-up copy, line 152 of clean copy):

“The intention of examining statin initiation by time-varying eligibility was to enhance the validity of the study by selecting a denominator sample for each period which reflected appropriate prescribing at the time.”

In answer to the reviewer’s query, anyone diagnosed with type 2 diabetes during the 2006-2008 period would be eligible for statin prescription and thus included in the study. If someone was diagnosed with type 2 diabetes in the 2008-2014 period, they would be eligible for statin prescription, and thus inclusion, if they were ≥40 years old or had a 10 year ASCVD risk of ≥20% or had ≥1 other ASCVD risk factor. If someone was diagnosed with type 2 diabetes in the 2014-2019 period, they would be eligible for statin prescription, and thus inclusion, if they had an ASCVD risk of ≥10%. In line with this, we have altered the following sentence in the “study population” part of the methods section (additions in bold font, line 154 of marked-up copy, line 141 of clean copy):

“Following this, we applied time-period dependent criteria to select the population in whom statins were indicated, using: NICE’s 2006 guideline^9^ (for people with index dates between 1st January 2006 and 31st May 2008**, all of whom were eligible for statin prescription, and thus inclusion**), 2008 guideline^8^ (for people with index dates between 1st June 2008 and 31st July 2014**, who were eligible for statin prescription, and thus inclusion, if they were ≥40 years old or had a 10 year ASCVD risk of ≥20% or ≥1 other ASCVD risk factor**) or 2014 guideline^10^ (for people with index dates between 1st August 2014 and 30th June 2019**, who were eligible for statin prescription, and thus inclusion, if they had a 10 year ASCVD risk of ≥10%**) (see table 1).”

We had already performed a sub-group analysis by time period, which we would like to draw the reviewer’s attention to (see figure 2c) and paragraph 4 of the results for interpretation of these findings (line 366 of marked-up copy, line 343 of clean copy):

“Similarly, ethnic differences appeared less marked for the latter 5 years of the study, though there was no statistical evidence of interaction by calendar period (ethnicity x latter time period interaction p=0.24 for South Asian people and p=0.51 for African/ African Caribbean people); these findings should be interpreted with caution as confidence intervals were very wide due to low power, and do in fact include the larger differences present for the prior periods.”

***5. Page 6. Before Covariates, I think you need a section on how your events were defined and how you computed the time to event. It seems that your baseline was changing over time depending on the NICE guidelines. Your time to event calculation needs to be well justified.***

Events were defined as the first ever statin prescription after the index date. We have moved this to a new section entitled “Outcome” in the results part of the paper The key message here is that follow-up started at the point when the individual became eligible for statin treatment, irrespective of the time period, an approach which we believe significantly enhances the validity of appropriate prescribing. The paragraph now read as follows (line 170 of marked-up copy, line 158 of clean copy):

“**Outcome**

Statin initiation was designated by a first ever statin prescription date on or after the index date (i.e. the date when follow-up started). In all cases (i.e. for each of the three time periods pertaining to 2006, 2008 and 2014 NICE guidelines), the index date was the date when the person became eligible for statin treatment, according to the relevant guideline in use at the time (see above). By using the first date of eligibility for statin treatment as the index date (irrespective of what factors governed that eligibility), we aimed to produce a focused outcome of appropriate prescribing. This enabled a fair comparison of the three ethnic groups across all time periods of the proportion of those who should have received a statin who actually did so; important as proportions entering the study from each ethnic group varied with guideline-related time periods.”

***6. Page 6, "For HbA1c and BMI, we also used ..." This sentence is redundant.***

Thanks for spotting this repetition – it has been removed.

***7. Page 6, last paragraph. How were people with harmful side effects of statins handled? What was the percentage of people with non-statin cholesterol-lowering medications? No excluding these patients from the analysis may have confounded the study results.***

Thanks for these points.

Re. harmful side effects: as stated in the “covariates” part of the methods section of the initial version of the manuscript “for those who did not receive a statin throughout follow-up, presence of a recorded reason for statin declinature was noted.” This was then used in a sensitivity analysis which excluded the people with a recorded reason for statin declinature (see supplementary figure 2e); results were unaltered. In response to the reviewer’s query, we have now added the following detail to the “covariates” part of the methods section (line 229 of marked-up copy, line 215 of clean copy):

“…. (this variable conflated the presence of one or more of the following codes in the health record: statin contraindicated (3%), statin declined (80%), statin not tolerated (21%), statin purchased over the counter (1%).)”

Re. the percentage of people with non-statin cholesterol-lowering medications: we would like to draw the reviewer’s attention to the following sentence in the initial version of the manuscript, and hope you’ll agree the small number of individuals this concern pertains to means that bias for this reason is unlikely (line 180 of marked-up copy, line 168 of clean copy):

“Other lipid-lowering medications were not considered, since they are generally second-line drugs (e.g. ezetimibe, for which there were very small numbers of incident prescriptions in people not on statins: n=38, n<5, n<5 for people of European, South Asian and African/ African Caribbean ethnicity respectively) or their use falls outside of all NICE guidelines pertinent to the study, and thus they are rarely initiated in primary care.”

***8. Page 7, top of the page. What was the missing rates for the ethnicity variable? If the rate is high, the complete case analysis may be biased. Also, as the authors described it, if missing values were not MAR, complete case analysis is even more troubling. Can you present analysis based on all records by assigning missing values in a separate category whenever possible? For example, missing ethnicity in the "unknown" category.***

Thanks for these comments. We have now added two tables (baseline characteristics and comparison of hazards ratios with the European ethnicity group) to the supporting information regarding people with incident type 2 diabetes after 2006 who were eligible for statin prescription, see excerpts below:

**Table S4. Baseline characteristics of people with incident type 2 diabetes after 2006 with no prior statin use or ASCVD, by ethnicity, including missing ethnicity (complete case analysis).** Data are n (age-standardised %), age-adjusted means±SD or median (IQR).

|  | **European ethnicity** | **South Asian ethnicity** | **African/ African Caribbean**  **ethnicity** | **Missing ethnicity** |
| --- | --- | --- | --- | --- |
| **N** | 27,511 (48) | 2,386 (4) | 1,142 (2) | 25,847 (45) |
| **Ethnic sub-group** | British: 26,238 (95)  Irish: 257 (1)  Other/ not stated white: 1,016 (4) | Indian: 932 (39)  Pakistani: 628 (26)  Bangladeshi: 213 (9)  Other/ not stated South Asian: 612(26) | Caribbean: 420 (37)  African: 553 (48)  Other/ not stated black: 170 (15) | - |
| **Age, yrs** | 59±12 | 50±11 | 52±11 | 59±12 |
| **Age group**  ≤45 years  >45 to ≤65 years  >65 years | 4,009 (15)  15,505 (56)  7,997 (29) | 888 (37)  1,276 (53)  222 (9) | 307 (27)  679 (59)  156 (14) | 3,959 (15)  14,362 (56)  7,526 (29) |
| **Gender**  Male  Female | 15,249 (53)  12,262 (47) | 1,307 (53)  1,079 (47) | 589 (51)  553 (49) | 14,172 (53)  11,675 (47) |
| **Practice location**  Non-London  London | 24,861 (91)  2,650 (9) | 1,467 (66)  919 (34) | 440 (44)  702 (56) | 24,733 (96)  1,114 (4) |
| **Country**  England  Scotland  Wales  Northern Ireland | 19,205 (68)  5,464 (20)  2,540 (10)  302 (2) | 2,163 (90)  128(4)  93(5)  2 (0.01) | 1,093 (95)  25 (2)  21 (3)  3 (0.01) | 15,688 (60)  2,615 (9)  5.725 (22)  1,819 (8) |
| **Deprivation, practice IMD quintile**  1 (least deprived)  2  3  4  5 (most deprived) | 3,766 (12)  4,713 (15)  5,130 (18)  6,118 (22)  7,784 (32) | 203 (7)  287 (11)  505 (19)  700 (26)  691 (37) | 46 (3)  108 (10)  182 (15)  360 (30)  446 (42) | 4,854 (17)  3,985 (15)  5,311 (20)  5,351 (21)  6,346 (27) |
| **Smoking**  Never  Ex  Current | 9,829 (38)  12,721 (39)  4,961 (23) | 1,510 (61)  567 (23)  309 (16) | 665 (58)  350 (29)  127 (13) | 10,379 (42)  11,206 (37)  4,262 (22) |
| **Number of consultations in previous year:**  **Median (IQR)**  **Quartiles**  1 (0 to 3)  2 (4 to 7)  3 (8 to 12)  4 (13 +) | 7 (4-12)  5,901 (21)  8,540 (29)  6,702 (24)  6,368 (25) | 6 (3-10)  607 (27)  772 (29)  577 (29)  430 (16) | 6 (3-10)  309 (24)  373 (28)  250 (19)  210 (29) | 6 (3-11)  5,674 (22)  6,723 (25)  7,240 (28)  6,210 (26) |
| **Total: HDL cholesterol** | 4.96±1.59 | 4.67±1.45 | 4.21±1.43 | 4.93±1.52 |
| **Non-HDL cholesterol, mmol/l** | 4.38±1.18 | 4.16±1.04 | 3.97±1.12 | 4.36±1.19 |
| **HbA_1c_:**  %  mmol/mol | 7.8±2.1  61±23 | 7.6±2.0  59±23 | 7.8±2.3  60±25 | 7.9±2.1  62±24 |
| **BMI, kg/m^2^**  **Underweight/ Normal**  **Overweight**  **Obese** | 34±7  2,262 (9)  7,074 (21)  18,175 (71) | 28±6  183 (12)  679 (25)  1,524 (63) | 31±6  34 (5)  205 (17)  903 (78) | 33±7  2,279 (8)  6,735 (22)  16,833 (70) |
| **CKD Read code** | 1,058 (4) | 41 (3) | 50 (6) | 1, 334 (5) |
| **Cancer** | 1,902 (6) | 48 (2) | 56 (6) | 1,794 (5) |
| **Asthma/ COPD** | 4,175 (17) | 307 (17) | 131 (12) | 5,721 (24) |
| **Serious mental illness** | 1,405 (7) | 74 (4) | 63 (7) | 1,181 (6) |
| **Number of different medications prescribed in previous year:**  **Median (IQR)**  **Quartiles**  1 (0 to 2 medications)  2 (3 to 5 medications)  3 (6 to 9 medications)  4 (10 + medications) | 6 (3-10)  5,527 (20)  7,607 (27)  6,929 (25)  7,448 (28) | 5 (2-9)  605 (22)  620 (22)  552 (29)  609 (28) | 5 (2-9)  287 (23)  333 (25)  267 (21)  255 (31) | 5(3-10)  7,970 (31)  6,761 (26)  6,943 (27)  4,173 (17) |
| **Antihypertensive use** | 12,477 (36) | 620 (29) | 516 (41) | 12,339 (37) |
| **Recorded reason for declinature (if statin not prescribed)** | 1,181/8,708 (10) | 66/897 (6) | 30/537 (4) | 960 (9) |
| **Exception reported from diabetes Quality Outcomes Framework** | 1,186 (6) | 102 (7) | 54 (5) | 2,899 (12) |
| **Time period of index date, by NICE guideline**  1^st^ Jan 2006 to 31^st^ May 2008  1^st^ Jun 2008 to 31^st^ Jul 2014  1^st^ Aug 2014 to 30^th^ Jun 2019 | 5,583 (20)  17,849 (65)  4,079 (15) | 339 (14)  1,791 (75)  256 (11) | 167 (15)  856 (75)  119 (10) | 7,435 (29)  16,913 (65)  1,499 (6) |
| **N (%) initiating statin** | 18,803 (68) | 1,489 (62) | 605 (53) | 17,317 (67) |

**Table S5. Associations between ethnicity and guideline-indicated statin initiation after type 2 diabetes diagnosis. Data are HRs (95% CI), from multi-level models accounting for intra-practice clustering.**

| **Model factors** | **European ethnicity** | **Missing ethnicity** |
| --- | --- | --- |
| Age + gender | 1 | 0.99 (0.94,1.03) |
| Age + gender + deprivation | 1 | 0.99 (0.95,1.03) |
| Age + gender + smoking | 1 | 0.99 (0.95,1.03) |
| Age + gender + healthcare usage | 1 | 0.99 (0.95,1.03) |
| Age + gender + TC/ HDL | 1 | 0.99 (0.94,1.03) |
| Age + gender + BMI | 1 | 0.99 (0.94,1.03) |
| Age + gender + comorbidity | 1 | 0.99 (0.95,1.03) |
| Age + gender + polypharmacy + antihypertensive use | 1 | 0.99 (0.95,1.03) |
| All above covariates | 1 | 1.00 (0.95,1.04) |

Additionally, we have added the following to the results (line 316 of marked-up copy, line 294 of clean copy):

“People with missing ethnicity (n=25,847) were excluded from the study population, but comparisons with the European ethnic group are presented in tables S3 and S4.”

Table S4 shows the baseline characteristics of the population with missing ethnicity are similar to those of the European ethnic group. Notably, 12% (vs. 5-7% in the other groups) were exception-reported from the UK Quality Outcomes Framework (the mechanism by which primary care physicians are incentivised to record chronic disease parameters and achieve targets), which is likely to be related to lower recording rates of routine data such as ethnicity in this population. Further, the missing ethnicity group were much less to live in London (4% vs. 9% in the European ethnicity non-missing group). Most of the UK population outside London are of European ethnicity so ethnicity recording may be lower.

Two key situations in which selection bias due to missing ethnicity could have biased our results away from the null are if the people with missing ethnicity constituted: i) many people of South Asian or African/ African Caribbean ethnicity being over-prescribed statins relative to the South Asian or African/ African Caribbean groups in the main analysis, or ii) many people of European ethnicity being under-prescribed statins relative to the European group in the main analysis. The baseline characteristics of the missing ethnicity population are very similar to the European ethnicity group, making i) unlikely. Further, if we assume most of this population were of European ethnicity, as their characteristics indicate, we would therefore expect their rates of statin prescription to be similar to those of people of European ethnicity, and this is indeed the case (table S5). However, we do not wish to include this exploratory comparison of hazard ratios for the missing ethnicity population in the main body of the paper, and we advise a cautious interpretation of it, since the use of a separate missing category is not generally considered a valid method for dealing with missing data. It is not underpinned by statistical theory and can lead to unpredictable results (Carpenter et al 2021).

Regarding your concerns about the use of complete case analysis when data are not missing at random (here and in point 11, below), White and Carlin (White IR et al 2010), showed that if missingness is conditionally independent of the outcome, a complete case analysis will generally be unbiased, even if data are not missing at random (see also <http://thestatsgeek.com/wp-content/uploads/2016/08/Jonathan-Bartlett-28-06-2013.pdf>). This assumption is untestable because one cannot condition on the variable with missing data, but reassuringly, the crude proportion initiating statin treatment did not differ by missingness status (67% for the people with missing ethnicity vs. 68% in people of European ethnicity), therefore we believe it unlikely that our estimates using the complete case analysis will be biased. Furthermore, we think that the complete case assumption (conditional independence between missingness and outcome) is more plausible than the “missing at random” assumption needed for multiple imputation, since missingness of ethnicity in primary care data is likely to be associated with ethnicity itself. As such, we have added the following to the discussion (line 520 of marked-up copy, line 470 of clean copy):

“This study used only data from people with a recorded ethnicity of interest; we believe the complete case assumption (conditional independence between missingness and outcome) is more plausible than the “missing at random” assumption needed for multiple imputation, since missingness of ethnicity in primary care data is likely to be associated with ethnicity itself.”

***9. Page 7, middle of page. "Multi-level Cox ..." There is not justification for using multi-level model or any discussion about how the model was used. What was the cluster/group variable? Was it a random intercept model or something else?***

Thanks for this. We used fixed-effects Cox models with a sandwich estimator to produce robust standard errors accounting for clustering. We have elaborated on the reasons for doing this in the following sentence (line 268 of marked-up copy, line 244 of clean copy):

“Prescribing behaviour is similar for doctors working in the same practice^24^ (i.e. clustering of prescribing patterns), resulting in individuals attending the same practice not being independent of each other with respect to statin initiation, which may in term lead to underestimation of standard errors, i.e. excess type 1 error. To counteract this, we fitted models using robust standard errors, with practice as the cluster variable.”

***10. Page 11, predicted reduction in ASCVD risk is very helpful. Why not use this information for a bullet point in the "What this study adds"?***

Thank you, we have now added the following bullet-point to the “What did the researchers do and find?” section of the author summary (line 82 of marked-up copy, line 70 of clean copy):

“We estimated that equalising statin prescribing rates across these three ethnic groups would prevent up to 12,600 atherosclerotic cardiovascular disease events (such as heart attacks and strokes) in people with type 2 diabetes.”

We have also added the following to the abstract regarding this:

“…and the number of ASCVD events prevented by equalising prescribing rates across ethnic groups was estimated.” (line 33 of marked-up copy, line 22 of clean copy)

“We estimated that up to 12, 600 ASCVD events could be prevented in people with diabetes by equalising statin prescribing across ethnic groups.” (line 33 of marked-up copy, line 46 of clean copy)

***11. Page 12, bottom of the second paragraph. I do not agree with what was asserted here. Comparison of complete case sample with the entire sample in terms of covariates does not guarantee that the complete case analysis is not biased.***

Thanks for this point, we agree this comparison does not guarantee that the complete case analysis is valid. We have now removed any implication that the comparison of covariates was to check the validity of the complete case analysis, and re-worded as follows (line 258 of marked-up copy, line 237 of clean copy):

“Additionally, characteristics, where available, were compared between the complete case analysis sample and the sample for whom some missing data was present**, to explore important differences between those with and without missing data.**”

As per response to point 9, we have added further text to justify the use of complete case analysis over other methods, and further supplementary tables to provide reassurance about this approach.

***12. Page 12. Can you move the key strengths paragraph somewhere to the bottom of the discussion?***

Yes, we have now moved this to the penultimate paragraph of the discussion.

**REFERENCES**

Carpenter JR, Smuk M. Missing data: A statistical framework for practice. *Biometrical Journal* 2021. doi: 10.1002/bimj.202000196; 1-33.

Chapman et al. Ethnic variations in lipid-lowering in response to a statin (EVIREST): a substudy of the Anglo-Scandinavian Cardiac Outcomes Trial (ASCOT). *Ethn Dis* 2011;21(2):150-7.

Kim et al. Use and Interpretation of Propensity Scores in Aging Research: A Guide for Clinical Researchers. *J Am Geriatr Soc* 2016; 64(10): 2065–2073.

Mathur et al. Completeness and usability of ethnicity data in UK-based primary care and hospital databases. *J Public Health (Oxf*) 2014 Dec;36(4):684-92.

Perneger T. What’s wrong with Bonferroni adjustments? *BMJ* 1998; 316: 1231-8.

Wasserstein et al. Moving to a World Beyond “p < 0.05”. *The American Statistician* 2019; 73 (supp1):1-19.

Wasserstein et al 2019. The ASA Statement on p-Values: Context, Process, and Purpose. [Full article: The ASA Statement on p-Values: Context, Process, and Purpose (tandfonline.com)](https://amstat.tandfonline.com/doi/full/10.1080/00031305.2016.1154108#.YHvw_p-SmUl)

White IR, Carlin JB. Bias and efficiency of multiple imputation compared with complete-case analysis for missing covariate values. *Stat Med* 2010 Dec 10;29(28):2920-31.
