# Supplementary material for: Ethnic differences in guideline-indicated statin initiation for people with type 2 diabetes in UK primary care, 2006–2019: A cohort study
Source: PLoS Med. 2021 Jun 29;18(6):e1003672. doi: 10.1371/journal.pmed.1003672 (PMC8241069; doi:10.1371/journal.pmed.1003672)
Supplement: Attachment — Submitted filename: Response_to_reviewers_#2_May2021.docx [file pmed.1003672.s014.docx]

***PLOS Medicine
Sent: 14 May 2021 20:15
To: Eastwood, Sophie <***[***sophie.eastwood@ucl.ac.uk***](mailto:sophie.eastwood@ucl.ac.uk)***>
Subject: PMEDICINE-D-21-00613R2 Decision - [EMID:e431217e85bac71a]

Dear Dr. Eastwood,

Thank you very much for re-submitting your manuscript "Ethnic differences in guideline-indicated statin initiation for people with type 2 diabetes: a retrospective cohort study using the Clinical Practice Research Datalink, 2006-2019." (PMEDICINE-D-21-00613R2) for review by PLOS Medicine.

I have discussed the paper with my colleagues and the academic editor and it was also seen again by two reviewers. I am pleased to say that provided the remaining editorial and production issues are dealt with we are planning to accept the paper for publication in the journal.

The remaining issues that need to be addressed are listed at the end of this email. Any accompanying reviewer attachments can be seen via the link below. Please take these into account before resubmitting your manuscript:


***Please note while forming your response, if your article is accepted, you may have the opportunity to make the peer review history publicly available. The record will include editor decision letters (with reviews) and your responses to reviewer comments. If eligible, we will contact you to opt in or out.***

In revising the manuscript for further consideration here, please ensure you address the specific points made by each reviewer and the editors. In your rebuttal letter you should indicate your response to the reviewers' and editors' comments and the changes you have made in the manuscript. Please submit a clean version of the paper as the main article file. A version with changes marked must also be uploaded as a marked up manuscript file.

Please also check the guidelines for revised papers at*** [***https://eur01.safelinks.protection.outlook.com/?url=http%3A%2F%2Fjournals.plos.org%2Fplosmedicine%2Fs%2Frevising-your-manuscript&amp;data=04%7C01%7C%7C61490278b5ed40c6b3f308d9170c8501%7C1faf88fea9984c5b93c9210a11d9a5c2%7C0%7C0%7C637566164817459954%7CUnknown%7CTWFpbGZsb3d8eyJWIjoiMC4wLjAwMDAiLCJQIjoiV2luMzIiLCJBTiI6Ik1haWwiLCJXVCI6Mn0%3D%7C1000&amp;sdata=fKT5xL%2BLipqlFRidoHtSXqSCFYqhBmNgz%2Fxxi%2FdC5vo%3D&amp;reserved=0***](https://protect-eu.mimecast.com/s/dePUCG80qCBvQR3C070hQ?domain=eur01.safelinks.protection.outlook.com) ***for any that apply to your paper. If you haven't already, we ask that you provide a short, non-technical Author Summary of your research to make findings accessible to a wide audience that includes both scientists and non-scientists. The Author Summary should immediately follow the Abstract in your revised manuscript. This text is subject to editorial change and should be distinct from the scientific abstract.

We expect to receive your revised manuscript within 1 week. Please email us (***[***plosmedicine@plos.org***](mailto:plosmedicine@plos.org)***) if you have any questions or concerns.

We ask every co-author listed on the manuscript to fill in a contributing author statement. If any of the co-authors have not filled in the statement, we will remind them to do so when the paper is revised. If all statements are not completed in a timely fashion this could hold up the re-review process. Should there be a problem getting one of your co-authors to fill in a statement we will be in contact. YOU MUST NOT ADD OR REMOVE AUTHORS UNLESS YOU HAVE ALERTED THE EDITOR HANDLING THE MANUSCRIPT TO THE CHANGE AND THEY SPECIFICALLY HAVE AGREED TO IT.

Please ensure that the paper adheres to the PLOS Data Availability Policy (see*** [***https://eur01.safelinks.protection.outlook.com/?url=http%3A%2F%2Fjournals.plos.org%2Fplosmedicine%2Fs%2Fdata-availability&amp;data=04%7C01%7C%7C61490278b5ed40c6b3f308d9170c8501%7C1faf88fea9984c5b93c9210a11d9a5c2%7C0%7C0%7C637566164817459954%7CUnknown%7CTWFpbGZsb3d8eyJWIjoiMC4wLjAwMDAiLCJQIjoiV2luMzIiLCJBTiI6Ik1haWwiLCJXVCI6Mn0%3D%7C1000&amp;sdata=5dPNBqtEwHsxjN4i29idOFNfUlwmvfZIf5pXFGFQGU0%3D&amp;reserved=0***](https://protect-eu.mimecast.com/s/vbvyCJQBwUBNDv9CpBgob?domain=eur01.safelinks.protection.outlook.com)***), which requires that all data underlying the study's findings be provided in a repository or as Supporting Information. For data residing with a third party, authors are required to provide instructions with contact information for obtaining the data. PLOS journals do not allow statements supported by "data not shown" or "unpublished results." For such statements, authors must provide supporting data or cite public sources that include it.

To enhance the reproducibility of your results, we recommend that you deposit your laboratory protocols in protocols.io, where a protocol can be assigned its own identifier (DOI) such that it can be cited independently in the future. Additionally, PLOS ONE offers an option to publish peer-reviewed clinical study protocols. Read more information on sharing protocols at*** [***https://eur01.safelinks.protection.outlook.com/?url=https%3A%2F%2Fplos.org%2Fprotocols%3Futm_medium%3Deditorial-email%26utm_source%3Dauthorletters%26utm_campaign%3Dprotocols&amp;data=04%7C01%7C%7C61490278b5ed40c6b3f308d9170c8501%7C1faf88fea9984c5b93c9210a11d9a5c2%7C0%7C0%7C637566164817459954%7CUnknown%7CTWFpbGZsb3d8eyJWIjoiMC4wLjAwMDAiLCJQIjoiV2luMzIiLCJBTiI6Ik1haWwiLCJXVCI6Mn0%3D%7C1000&amp;sdata=AXH%2BTmxqLXO0mxKbddyUOprOtF0xZyglkAZBUIFnrOU%3D&amp;reserved=0***](https://protect-eu.mimecast.com/s/4P0qCKQDxUDVGN7Sns71r?domain=eur01.safelinks.protection.outlook.com) ***Please review your reference list to ensure that it is complete and correct. If you have cited papers that have been retracted, please include the rationale for doing so in the manuscript text, or remove these references and replace them with relevant current references. Any changes to the reference list should be mentioned in the rebuttal letter that accompanies your revised manuscript.***

Thank you, we have checked the reference list, it was satisfactory therefore no changes have been made. ***Please note, when your manuscript is accepted, an uncorrected proof of your manuscript will be published online ahead of the final version, unless you've already opted out via the online submission form. If, for any reason, you do not want an earlier version of your manuscript published online or are unsure if you have already indicated as such, please let the journal staff know immediately at*** [***plosmedicine@plos.org***](mailto:plosmedicine@plos.org)***.

If you have any questions in the meantime, please contact me or the journal staff on*** [***plosmedicine@plos.org***](mailto:plosmedicine@plos.org)***.  

We look forward to receiving the revised manuscript by May 21 2021 11:59PM.   

Sincerely,

Beryne Odeny,
Associate Editor 
PLOS Medicine
plosmedicine.org

------------------------------------------------------------
Requests from Editors:

1. Please revise your title to include the country/setting***

Thank you, we have now changed the title from:

“Ethnic differences in guideline-indicated statin initiation for people with type 2 diabetes: a retrospective cohort study using the Clinical Practice Research Datalink, 2006-2019.”

to:

“Ethnic differences in guideline-indicated statin initiation for people with type 2 diabetes **in UK primary care**: a retrospective cohort study using the Clinical Practice Research Datalink, 2006-2019.”

***2. Please trim your author summary bullet points***

Thanks, we have done this, the author summary now read as follows (lines 48-83 on the marked-up copy):

“Why was this study done?

- People of South Asian and African/ African Caribbean ethnicity living in the UK are more likely to have type 2 diabetes than people of European ethnicity, and have higher rates of the cardiovascular complications of diabetes, e.g. heart attacks and strokes.
- Lowering blood cholesterol with statin treatment reduces cardiovascular complications for people with type 2 diabetes, but previous studies suggest ethnic differences exist in statin prescribing for people with diabetes.
- However, no study has sought explanations for identified ethnic differences, or accounted for changes in prescribing guidelines, so we aimed to provide a timely, nationally representative assessment of ethnic differences in guideline-indicated statin prescribing in type 2 diabetes.

What did the researchers do and find?

- We identified people with newly diagnosed type 2 diabetes in primary care who were eligible for statin treatment for primary prevention of cardiovascular disease, then compared rates of statin initiation for people of European, South Asian and African/ African Caribbean ethnicity.
- People of African/ African Caribbean ethnicity with type 2 diabetes were 24% less likely to receive guideline-indicated statin treatment than people of European ethnicity, and people of South Asian ethnicity were 9% less likely.
- Ethnic differences remained after allowing for differences in cholesterol levels, other cardiovascular risk factors, demographic factors, deprivation, healthcare usage, comorbidity and polypharmacy.
- We estimated that equalising statin prescribing rates across these three ethnic groups would prevent up to 12,600 heart attacks and strokes over the lifetimes of people currently affected by type 2 diabetes in the UK.

What do these findings mean?

- People with type 2 diabetes of African/ African Caribbean and South Asian ethnicities are less likely to be prescribed a statin for primary prevention of cardiovascular disease in the UK than people of European ethnicity.
- Policies to increase statin use use among people of African/ African Caribbean and South Asian ethnicity with type 2 diabetes could substantially reduce the excess burden of cardiovascular events in these groups.

***3. Please add the following statement, or similar, to the Methods: "******This study is reported as per the Strengthening the Reporting of Observational Studies in Epidemiology (STROBE) guideline (S1 Checklist)."***

Thanks, we have done this (lines 285-6 on the marked-up copy).

***4. Please provide the meaning of abbreviations in tables (Table S3, S4, S5 e.g., TC, HDL, BMI, CKD, COPD, NICE, HbA1c***

Thanks, we have now expanded all abbreviations in the footnotes of tables:1-2, S1-5 and figures S1-S2. These changes are tracked on the marked-up copy of the manuscript but have been simply added to the supplementary tables and figures.

***5. Please provide 95% CIs and p values for all estimates in the main text and tables.***

Thanks, we have now provided 95% Ci and p values for all estimates in the text and the main figures.

***6. Thank you for providing your STROBE checklist. Please replace the page numbers with paragraph numbers per section (e.g. "Methods, paragraph 1"), since the page numbers of the final published paper may be different from the page numbers in the current manuscript.***

This has been done.

***7. Please use the "Vancouver" style for reference formatting and see our website for other reference guidelines*** [***https://eur01.safelinks.protection.outlook.com/?url=https%3A%2F%2Fjournals.plos.org%2Fplosmedicine%2Fs%2Fsubmission-guidelines%23loc-references&amp;data=04%7C01%7C%7C61490278b5ed40c6b3f308d9170c8501%7C1faf88fea9984c5b93c9210a11d9a5c2%7C0%7C0%7C637566164817469947%7CUnknown%7CTWFpbGZsb3d8eyJWIjoiMC4wLjAwMDAiLCJQIjoiV2luMzIiLCJBTiI6Ik1haWwiLCJXVCI6Mn0%3D%7C1000&amp;sdata=CYguzZfp8gNBjGHHiCBNVPQcneGRpOrghu3EAFUXhVk%3D&amp;reserved=0***](https://protect-eu.mimecast.com/s/dhAICLJEyfk6ZrBt1TJE4?domain=eur01.safelinks.protection.outlook.com) ***. Please ensure that weblinks are current and accessible***

Thanks, this has been done.

***Comments from Reviewers:

Reviewer #1: The authors have addressed my concerns and I now recommend publication.

Peter Flom


Reviewer #2: The authors have properly addressed the questions raised by this reviewer


Reviewer #3: This revised version satisfactorily addressed all of my comments. I do not have any more concerns.


Any attachments provided with reviews can be seen via the following link:

__________________________________________________
In compliance with data protection regulations, you may request that we remove your personal registration details at any time. (Use the following URL:*** [***https://eur01.safelinks.protection.outlook.com/?url=https%3A%2F%2Fwww.editorialmanager.com%2Fpmedicine%2Flogin.asp%3Fa%3Dr&amp;data=04%7C01%7C%7C61490278b5ed40c6b3f308d9170c8501%7C1faf88fea9984c5b93c9210a11d9a5c2%7C0%7C0%7C637566164817469947%7CUnknown%7CTWFpbGZsb3d8eyJWIjoiMC4wLjAwMDAiLCJQIjoiV2luMzIiLCJBTiI6Ik1haWwiLCJXVCI6Mn0%3D%7C1000&amp;sdata=fahaJV3qmconaMWGLhzqwh2HG%2BVInVd7R%2F3IGNYeuXQ%3D&amp;reserved=0***](https://protect-eu.mimecast.com/s/R1juCM1Gzc2NA1BCRp_K6?domain=eur01.safelinks.protection.outlook.com)***). Please contact the publication office if you have any questions.***
