# Supplementary material for: Ethnic differences in guideline-indicated statin initiation for people with type 2 diabetes in UK primary care, 2006–2019: A cohort study
Source: PLoS Med. 2021 Jun 29;18(6):e1003672. doi: 10.1371/journal.pmed.1003672 (PMC8241069; doi:10.1371/journal.pmed.1003672)
Supplement: Attachment — Submitted filename: Response_to_reviewers_#24_May2021.docx [file pmed.1003672.s015.docx]

***Dear Dr. Eastwood,***

***Thank you very much for re-submitting your manuscript "Ethnic differences in guideline-indicated statin initiation for people with type 2 diabetes in UK primary care: a retrospective cohort study using the Clinical Practice Research Datalink, 2006-2019." (PMEDICINE-D-21-00613R3) for review by PLOS Medicine.***

***I have discussed the paper with my colleagues and the academic editor and it was also seen again by three reviewers. I am pleased to say that provided the remaining editorial and production issues are dealt with we are planning to accept the paper for publication in the journal.***

***The remaining issues that need to be addressed are listed at the end of this email. Any accompanying reviewer attachments can be seen via the link below. Please take these into account before resubmitting your manuscript:***

******Please note while forming your response, if your article is accepted, you may have the opportunity to make the peer review history publicly available. The record will include editor decision letters (with reviews) and your responses to reviewer comments. If eligible, we will contact you to opt in or out.******

***In revising the manuscript for further consideration here, please ensure you address the specific points made by each reviewer and the editors. In your rebuttal letter you should indicate your response to the reviewers' and editors' comments and the changes you have made in the manuscript. Please submit a clean version of the paper as the main article file. A version with changes marked must also be uploaded as a marked up manuscript file.***

***Please also check the guidelines for revised papers at*** [***https://eur01.safelinks.protection.outlook.com/?url=http%3A%2F%2Fjournals.plos.org%2Fplosmedicine%2Fs%2Frevising-your-manuscript&amp;data=04%7C01%7C%7C5b4da034320b44f6658108d91b9ff48a%7C1faf88fea9984c5b93c9210a11d9a5c2%7C0%7C0%7C637571198080265964%7CUnknown%7CTWFpbGZsb3d8eyJWIjoiMC4wLjAwMDAiLCJQIjoiV2luMzIiLCJBTiI6Ik1haWwiLCJXVCI6Mn0%3D%7C1000&amp;sdata=gWGLH%2Fh%2FV1n7NAQ%2F7XxMvmZyOScNu%2Bj0xS7%2BI7x2olI%3D&amp;reserved=0***](https://eur01.safelinks.protection.outlook.com/?url=http%3A%2F%2Fjournals.plos.org%2Fplosmedicine%2Fs%2Frevising-your-manuscript&amp;data=04%7C01%7C%7C5b4da034320b44f6658108d91b9ff48a%7C1faf88fea9984c5b93c9210a11d9a5c2%7C0%7C0%7C637571198080265964%7CUnknown%7CTWFpbGZsb3d8eyJWIjoiMC4wLjAwMDAiLCJQIjoiV2luMzIiLCJBTiI6Ik1haWwiLCJXVCI6Mn0%3D%7C1000&amp;sdata=gWGLH%2Fh%2FV1n7NAQ%2F7XxMvmZyOScNu%2Bj0xS7%2BI7x2olI%3D&amp;reserved=0) ***for any that apply to your paper. If you haven't already, we ask that you provide a short, non-technical Author Summary of your research to make findings accessible to a wide audience that includes both scientists and non-scientists. The Author Summary should immediately follow the Abstract in your revised manuscript. This text is subject to editorial change and should be distinct from the scientific abstract.***

***We expect to receive your revised manuscript within 1 week. Please email us (***[***plosmedicine@plos.org***](mailto:plosmedicine@plos.org)***) if you have any questions or concerns.***

***We ask every co-author listed on the manuscript to fill in a contributing author statement. If any of the co-authors have not filled in the statement, we will remind them to do so when the paper is revised. If all statements are not completed in a timely fashion this could hold up the re-review process. Should there be a problem getting one of your co-authors to fill in a statement we will be in contact. YOU MUST NOT ADD OR REMOVE AUTHORS UNLESS YOU HAVE ALERTED THE EDITOR HANDLING THE MANUSCRIPT TO THE CHANGE AND THEY SPECIFICALLY HAVE AGREED TO IT.***

***Please ensure that the paper adheres to the PLOS Data Availability Policy (see*** [***https://eur01.safelinks.protection.outlook.com/?url=http%3A%2F%2Fjournals.plos.org%2Fplosmedicine%2Fs%2Fdata-availability&amp;data=04%7C01%7C%7C5b4da034320b44f6658108d91b9ff48a%7C1faf88fea9984c5b93c9210a11d9a5c2%7C0%7C0%7C637571198080265964%7CUnknown%7CTWFpbGZsb3d8eyJWIjoiMC4wLjAwMDAiLCJQIjoiV2luMzIiLCJBTiI6Ik1haWwiLCJXVCI6Mn0%3D%7C1000&amp;sdata=oL8JZZWmL8%2B3E7bYRQ5SnrUlLFHp1%2FGeY%2BH6vAmqd94%3D&amp;reserved=0***](https://eur01.safelinks.protection.outlook.com/?url=http%3A%2F%2Fjournals.plos.org%2Fplosmedicine%2Fs%2Fdata-availability&amp;data=04%7C01%7C%7C5b4da034320b44f6658108d91b9ff48a%7C1faf88fea9984c5b93c9210a11d9a5c2%7C0%7C0%7C637571198080265964%7CUnknown%7CTWFpbGZsb3d8eyJWIjoiMC4wLjAwMDAiLCJQIjoiV2luMzIiLCJBTiI6Ik1haWwiLCJXVCI6Mn0%3D%7C1000&amp;sdata=oL8JZZWmL8%2B3E7bYRQ5SnrUlLFHp1%2FGeY%2BH6vAmqd94%3D&amp;reserved=0)***), which requires that all data underlying the study's findings be provided in a repository or as Supporting Information. For data residing with a third party, authors are required to provide instructions with contact information for obtaining the data. PLOS journals do not allow statements supported by "data not shown" or "unpublished results." For such statements, authors must provide supporting data or cite public sources that include it.***

***To enhance the reproducibility of your results, we recommend that you deposit your laboratory protocols in protocols.io, where a protocol can be assigned its own identifier (DOI) such that it can be cited independently in the future. Additionally, PLOS ONE offers an option to publish peer-reviewed clinical study protocols. Read more information on sharing protocols at*** [***https://eur01.safelinks.protection.outlook.com/?url=https%3A%2F%2Fplos.org%2Fprotocols%3Futm_medium%3Deditorial-email%26utm_source%3Dauthorletters%26utm_campaign%3Dprotocols&amp;data=04%7C01%7C%7C5b4da034320b44f6658108d91b9ff48a%7C1faf88fea9984c5b93c9210a11d9a5c2%7C0%7C0%7C637571198080265964%7CUnknown%7CTWFpbGZsb3d8eyJWIjoiMC4wLjAwMDAiLCJQIjoiV2luMzIiLCJBTiI6Ik1haWwiLCJXVCI6Mn0%3D%7C1000&amp;sdata=V3QY5jbGwrWRQauCM9X6WXE3WlUOEWiOKlMhhK7u%2B30%3D&amp;reserved=0***](https://eur01.safelinks.protection.outlook.com/?url=https%3A%2F%2Fplos.org%2Fprotocols%3Futm_medium%3Deditorial-email%26utm_source%3Dauthorletters%26utm_campaign%3Dprotocols&amp;data=04%7C01%7C%7C5b4da034320b44f6658108d91b9ff48a%7C1faf88fea9984c5b93c9210a11d9a5c2%7C0%7C0%7C637571198080265964%7CUnknown%7CTWFpbGZsb3d8eyJWIjoiMC4wLjAwMDAiLCJQIjoiV2luMzIiLCJBTiI6Ik1haWwiLCJXVCI6Mn0%3D%7C1000&amp;sdata=V3QY5jbGwrWRQauCM9X6WXE3WlUOEWiOKlMhhK7u%2B30%3D&amp;reserved=0)

***Please review your reference list to ensure that it is complete and correct. If you have cited papers that have been retracted, please include the rationale for doing so in the manuscript text, or remove these references and replace them with relevant current references. Any changes to the reference list should be mentioned in the rebuttal letter that accompanies your revised manuscript.***

***Please note, when your manuscript is accepted, an uncorrected proof of your manuscript will be published online ahead of the final version, unless you've already opted out via the online submission form. If, for any reason, you do not want an earlier version of your manuscript published online or are unsure if you have already indicated as such, please let the journal staff know immediately at*** [***plosmedicine@plos.org***](mailto:plosmedicine@plos.org)***.***

***If you have any questions in the meantime, please contact me or the journal staff on*** [***plosmedicine@plos.org***](mailto:plosmedicine@plos.org)***.***

***We look forward to receiving the revised manuscript by May 27 2021 11:59PM.***

***Sincerely,***

***Beryne Odeny,***

***Associate Editor***

***PLOS Medicine***

***plosmedicine.org***

***------------------------------------------------------------***

***Requests from Editors:***

***Thank you for your revisions. Please make this final set of revisions before acceptance:***

1. ***Please trim the title and modify it to, “Ethnic differences in guideline-indicated statin initiation for people with type 2 diabetes in UK primary care, 2006-2019: A cohort study”***

Thanks, this has been done.

***2.	Please provide the meaning of the abbreviation "HR" in Figure S4***

Thanks, this has been done.

***3.	Please correct reference #31 and remove excess text after the citation***

***4.	Please edit #40 and remove the paragraph on “***[***https://eur01.safelinks.protection.outlook.com/?url=http%3A%2F%2Fwww.icmje.org%2Fcoi_disclosure.pdf&amp;data=04%7C01%7C%7C5b4da034320b44f6658108d91b9ff48a%7C1faf88fea9984c5b93c9210a11d9a5c2%7C0%7C0%7C637571198080265964%7CUnknown%7CTWFpbGZsb3d8eyJWIjoiMC4wLjAwMDAiLCJQIjoiV2luMzIiLCJBTiI6Ik1haWwiLCJXVCI6Mn0%3D%7C1000&amp;sdata=VJmDcnNFyZSPBIr5kfa2ouuE87d1NJk8nqnW6CY89uU%3D&amp;reserved=0 and declare:***](https://eur01.safelinks.protection.outlook.com/?url=http%3A%2F%2Fwww.icmje.org%2Fcoi_disclosure.pdf&amp;data=04%7C01%7C%7C5b4da034320b44f6658108d91b9ff48a%7C1faf88fea9984c5b93c9210a11d9a5c2%7C0%7C0%7C637571198080265964%7CUnknown%7CTWFpbGZsb3d8eyJWIjoiMC4wLjAwMDAiLCJQIjoiV2luMzIiLCJBTiI6Ik1haWwiLCJXVCI6Mn0%3D%7C1000&amp;sdata=VJmDcnNFyZSPBIr5kfa2ouuE87d1NJk8nqnW6CY89uU%3D&amp;reserved=0%20and%20declare:)***”***

Apologies for this oversight, the excess text after references #31 and #40 has now been removed.

***Comments from Reviewers:***

***Any attachments provided with reviews can be seen via the following link:***

***__________________________________________________***

***In compliance with data protection regulations, you may request that we remove your personal registration details at any time. (Use the following URL:*** [***https://eur01.safelinks.protection.outlook.com/?url=https%3A%2F%2Fwww.editorialmanager.com%2Fpmedicine%2Flogin.asp%3Fa%3Dr&amp;data=04%7C01%7C%7C5b4da034320b44f6658108d91b9ff48a%7C1faf88fea9984c5b93c9210a11d9a5c2%7C0%7C0%7C637571198080265964%7CUnknown%7CTWFpbGZsb3d8eyJWIjoiMC4wLjAwMDAiLCJQIjoiV2luMzIiLCJBTiI6Ik1haWwiLCJXVCI6Mn0%3D%7C1000&amp;sdata=xREPmzXiLLUKrm1G9JkTH1AVwdjHHj9QTA1vekQkzWk%3D&amp;reserved=0***](https://eur01.safelinks.protection.outlook.com/?url=https%3A%2F%2Fwww.editorialmanager.com%2Fpmedicine%2Flogin.asp%3Fa%3Dr&amp;data=04%7C01%7C%7C5b4da034320b44f6658108d91b9ff48a%7C1faf88fea9984c5b93c9210a11d9a5c2%7C0%7C0%7C637571198080265964%7CUnknown%7CTWFpbGZsb3d8eyJWIjoiMC4wLjAwMDAiLCJQIjoiV2luMzIiLCJBTiI6Ik1haWwiLCJXVCI6Mn0%3D%7C1000&amp;sdata=xREPmzXiLLUKrm1G9JkTH1AVwdjHHj9QTA1vekQkzWk%3D&amp;reserved=0)***). Please contact the publication office if you have any questions.***
